# Silicon/2D-material photodetectors: from near-infrared to mid-infrared

**DOI:** 10.1038/s41377-021-00551-4

**Published:** 2021-06-09

**Authors:** Chaoyue Liu, Jingshu Guo, Laiwen Yu, Jiang Li, Ming Zhang, Huan Li, Yaocheng Shi, Daoxin Dai

**Affiliations:** 1 State Key Laboratory for Modern Optical Instrumentation, Zhejiang Provincial Key Laboratory for Sensing Technologies, College of Optical Science and Engineering, International Research Center for Advanced Photonics, Zhejiang University, Zijingang Campus, Hangzhou, 310058 China; 2 Ningbo Research Institute, Zhejiang University, Ningbo, 315100 China

**Keywords:** Silicon photonics, Optical properties and devices, Optoelectronic devices and components

## Abstract

Two-dimensional materials (2DMs) have been used widely in constructing photodetectors (PDs) because of their advantages in flexible integration and ultrabroad operation wavelength range. Specifically, 2DM PDs on silicon have attracted much attention because silicon microelectronics and silicon photonics have been developed successfully for many applications. 2DM PDs meet the imperious demand of silicon photonics on low-cost, high-performance, and broadband photodetection. In this work, a review is given for the recent progresses of Si/2DM PDs working in the wavelength band from near-infrared to mid-infrared, which are attractive for many applications. The operation mechanisms and the device configurations are summarized in the first part. The waveguide-integrated PDs and the surface-illuminated PDs are then reviewed in details, respectively. The discussion and outlook for 2DM PDs on silicon are finally given.

## Introduction

Two-dimensional materials (2DMs) are the layered materials in which the atoms of different layers interact though van der waals (vdW) forces instead of chemical bonds^1^. Nowadays, the 2DM family has been growing rapidly and becomes a rich new-material system, including semi-metal 2DMs^2^, semiconductor 2DMs^3^, and insulator 2DMs^4^. 2DMs are found to have some unique advantages for constructing photodetectors (PDs)^5–7^, which are the key components in various optoelectronic systems for optical communications, optical imaging, and optical sensing. Currently, various 2DMs have been demonstrated to enable the photodetection for ultrabroad wavelength bands from visible light to THz wave^8^. More importantly, 2DMs can be integrated flexibly with various material platforms, such as silicon and silicon compounds^9,10^, III–V materials^11^, as well as flexible materials^7,12^. In addition, 2DM PDs are free of dangling bonds which usually cause the surface recombination and the increment of dark current^13^. Among various 2DM PDs reported in the past years, the Si/2DM PDs have drawn much attention particularly because of the following two reasons^9,10^. First, silicon provides an excellent platform for realizing PD read-out circuits based on microelectronics, enabling optoelectronic integration to be realized potentially. Second, silicon photonics has also been developed successfully to provide high-performance, high-intensity, and low-cost photonic integrated circuits due to its complementary-metal-oxide-semiconductor (CMOS)-compatibility^14,15^. As is well known, silicon photonics has been a key technology for many important applications in the near-infrared (NIR, e.g., 1.31/1.55 μm) and mid-infrared (MIR) wavelength bands, such as optical communications/interconnects^14,16^, laser radar^17^, lab-on-chip for sensing^18^, optical computing^19^, and on-chip quantum photonics^20^. It is desired to achieve PDs on silicon working in broad operation wavelength bands, which are determined by the bandgap of the photodetection material^21^. As for the commonly used bulk semiconductor materials, silicon^22^ and SiGe^23^ can work in the wavelength band below 1.07 and 1.6 μm, respectively. While it is possible to realize PDs at the wavelength up to the MIR-band by using III–V materials^24^ and HgCdTe^25^, their heterogeneous integration on silicon is usually difficult due to lattice mismatch. Fortunately, the emergence of 2DMs provides a new option to realize high-performance and low-cost PDs on silicon for broad wavelength bands.

In this paper, we review the recent progresses of silicon/2DM PDs particularly working in the windows from the NIR-band of 1.31/1.55 μm to the MIR-band of 2–6 μm, which are very attractive for silicon photonics^26^. It is widely known that the big family of 2DMs includes several categories, such as single-element 2DMs, transition metal dichalcogenides (TMDCs), posttransition metal chalcogenides, tetradymites, and metal halides^27,28^. For example, single-element 2DMs mainly include group IV materials (e.g., graphene (G), silicene, germanene), group V materials (e.g., phosphorene, arsenene, antimonene), and group VI material (e.g., tellurene (Te)). Similar to bulk semiconductors, 2DMs usually have their bandgaps determining the wavelength band for photodetection, as summarized in refs. ^3,6^. Interestingly, the bandgaps of 2DMs can be modified by band engineering^3^, such as strain engineering^12^, vertical electric field gating^29^, and chemical doping^3^. For the NIR/MIR photodetection, the commonly used 2DMs include graphene with a zero bandgap^30^, TMDCs with a bandgap of 1–2 eV^31^, and black phosphorus (BP) with a 0.3–2 eV bandgap^32^. In addition, some newly developed 2DMs are also available in potential, such as tellurene with a bandgap of 0.35–1.265 eV^33^, GeP with a bandgap of 0.51–1.68 eV^34^, and PtSe_2_ with a bandgap of 0–1.2 eV^35^.

Here this review article is organized as follows. In “Fundamentals: mechanisms and structures” section, the fundamentals about 2DM PDs are discussed. In “Waveguide-integrated silicon-2DM PDs” and “Surface-illuminated Si/2DM PDs” sections, waveguide-integrated PDs and surface-illuminated PDs are summarized and reviewed, respectively. In “Perspective and outlook” section, we discuss the future development and the potential applications for 2DM PDs. Finally, a short conclusion is made in “Conclusions” section.

## Fundamentals: mechanisms and structures

In this section, the fundamentals of 2DM PDs are discussed and reviewed, including the working mechanisms and the device structures. Currently, there have been a number of review articles on 2DM PDs^1,5,36^. Here we aim to give a comprehensive discussion for Si/2DM PDs by clarifying several puzzles and distinguishing those similar mechanisms. In addition, a systematic classification of those 2DM PDs reported is also given in terms of the device structures, in which way the connection between the mechanisms and the device configurations is established.

### Working mechanisms

**Fig. 1:**
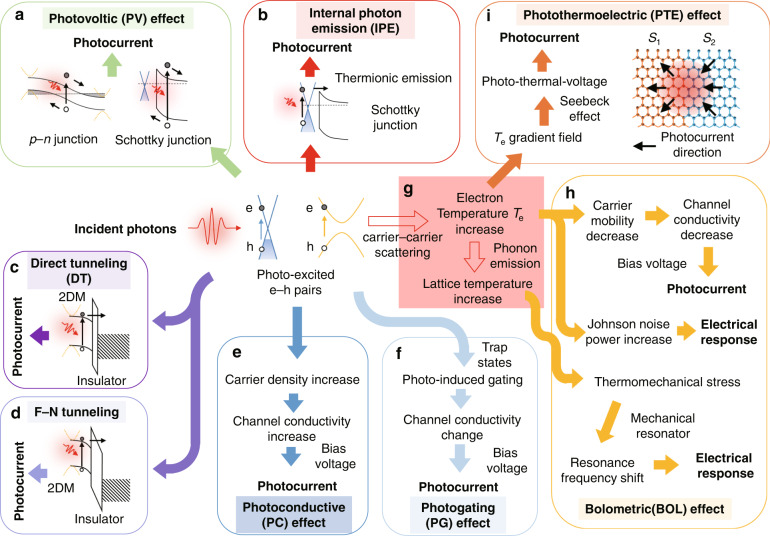
Summary of the working mechanisms of 2DM PDs. **a**–**f** Photon-type mechanisms: **a** The photovoltaic (PV) effect. The photo-excited *e*-*h* pairs driven by the built-in electric fields contribute to the photocurrents. **b** The internal photon emission (IPE) effect. For example, in a G-Si Schottky junction, the photo-excited hot carriers in graphene emitted over the Schottky barrier contribute to the photocurrent. **c** The direct tunneling (DT). **d** The Fowler-Nordheim (F-N) tunneling. **e** The photoconductive (PC) effect. The carrier density increment leads to the change of channel conductivity in a phototransistor. **f** The photo-gating (PG) effect. The photo-induced gating leads to the change of the channel conductivity in a phototransistor. **g** The thermal relaxation process in 2DMs. The carrier–carrier scattering results in the increment of the electron temperature *T*_e_, and then the optical/acoustic phonon emissions lead to the increment of lattice temperature *T*_L_. **h**, **i** Thermal-type mechanisms: **h** The bolometric (BOL) effect. The increment of *T*_e_ or *T*_L_ can be extracted by different read-outs. **i** The photo-thermoelectric (PTE) effect. Here Seebeck effect plays a major role. In the present example, *S*_1_ and *S*_2_ are respectively the positive and negative Seebeck coefficients.

As the field of 2DM PDs has been growing rapidly, the related technical routes have not been unified yet. The reported photodetection mechanisms for 2DM PDs include the photovoltaic (PV) effect^5,21,28,36,37^, the internal photon emission (IPE)^38,39^, the direct tunneling (DT)^37^, the Fowler-Nordheim (F-N) tunneling^37^, the photoconductive (PC) effect^5,6,21,36,37^, the photo-gating (PG) effect^5,6,36^, the bolometric (BOL) effect^5,6^, and the photo-thermoelectric (PTE) effect^5,6^. Most of these mechanisms have been applied or investigated very well in PDs based on traditional bulk materials, such as photoconductors, bolometers, or thermocouples. Figure 1 is a schematic diagram showing how the incident photons lead to electrical responses when working with different mechanisms. Basically speaking, when the incident photons are absorbed in the active region, the photo-excited electron-hole (*e*-*h*) pairs generate. The PDs can be classified into two types^2^, i.e., the photon-type and thermal-type, depending on whether the thermal relaxation process takes part in the photoelectric conversion process.

#### Photon-type PDs

For the photon-type PDs, the photoresponse is extracted by collecting the photo-excited carriers or detecting the photo-induced change of the channel conductivity. The former is available when working with the PV effect, IPE, DT, or F-N tunneling^37^, while the latter corresponds to the PC-type mechanisms (such as the PC effect and the PG effect) as well as the thermal-type BOL effect discussed below^5,6,36^.

For the PV effect, which is a kind of fundamental mechanism for bulk semiconductor photodiodes, the photo-excited *e*-*h* pairs are usually driven by the built-in electric fields formed in *p*-*n* junctions^5^ or Schottky junctions^38^, and then contribute to the photocurrents. A related concept is the impact ionization, which occurs when the photo-excited carriers are accelerated by a strong electric field and then lead to the carrier multiplications. It is the fundamental mechanism of avalanche photodetectors (APDs), and currently there have been several 2DM APDs reported^40^.

The IPE is the major working mechanism of the traditional Schottky PDs with the metal-semiconductor configuration, and is also known as the thermionic emission^39^. In IPE-based 2DM PDs, the photo-excited hot carriers with sufficient energy may be emitted over the Schottky barrier, and then contribute to the photocurrent^38^, as shown in Fig. 1b. As is well known, conventional Schottky PDs usually have a low internal quantum efficiency of <1%^37^, since only a small part of hot electrons generated from the light absorption in metal can be emitted when their normal components of momentum correspond to the kinetic energy overcoming the Schottky barrier^39^.

The DT and F-N tunneling are known as the operation mechanisms in traditional metal-insulator-semiconductor/metal tunneling diodes^37^. Now these two mechanisms have also been applied in 2DM PDs by introducing a 2DM-insulator configuration^41,42^, as shown in Fig. 1c, d. The DT occurs when the insulator layer is very thin (e.g., a sub-5-nm-thick SiO_2_ thin film^37^). For the F-N tunneling, the photo-excited carriers can transit through the triangle-shaped barrier of the insulator layer, which usually needs a high-bias voltage. For these tunneling PDs, the insulator layer can help reduce the dark current significantly to nanoampere scale^41^.

For the PC-type mechanisms, the incident photons cause the change of the channel electrical conductivities *σ*, and the photocurrents can then be extracted by applying a bias voltage. The electrical conductivity *σ* for 2DMs is given as *σ* = *neμ*, where *n*, *e*, and *μ* are respectively the carrier density, the unit charge, and the carrier mobility. For both PC and PG effects, the conductivity *σ* changes due to the change of the photo-induced carrier density *n*. However, there are some obvious differences between them. For the PC effect, both the carrier density *n* and the conductivity *σ* increase when light illuminates. Correspondingly, the total current *I*_tot_ under illumination is higher than the dark current *I*_dark_ when the bias voltage is applied. In this case, the photocurrent given by *I*_ph_ = *I*_tot_ − *I*_dark_ has the same direction as the applied bias voltage, as shown in Fig. 1e. In contrast, the key point in the PG effect is the trap states which may originate from the defects in 2DMs^43^ (e.g., vacancies, dislocations^44^), the 2DM/bulk-material junctions^45^, as well as the 2DM–2DM junctions^46^. For the PG effect, the electrons or holes of the photo-excited *e*-*h* pairs are trapped by the trap states with a long carrier life time *τ*_L_, during which the other polarity carriers can transit through the channel between the drain- and source-electrodes many times. Since the carrier life time *τ*_L_ is much longer than the carrier transit time *τ*_tr_, there is usually a PC gain up to 5–10^11^ (given by *Gain* = *τ*_L_/*τ*_tr_)^6^ and thus the PG photoresponse is as high as 10–10^10^ A W^−1,^^5–7^. In phototransistors with the source-, drain- and gate-electrodes, the trapped carriers causing the shift of the neutral-point gate-voltage can be treated as an extra gate voltage applied by the incident photons^36^. As a result, the direction of the photocurrent is decided by not only the applied bias voltage but also the original doping level of the channel 2DM. The process of the PG effect is also shown in Fig. 1f.

#### Thermal-type PDs

As illustrated in Fig. 1g, the thermal relaxation process in 2DMs is briefly described by a well-known two-temperature system^47–49^. After the photo-excited *e*-*h* pairs generate under illumination, the electron heating occurs by the carrier–carrier scattering, leading to the increment of the electron temperature *T*_e_. After then, the energy transfers from the electronic system to the phonon system by the electron-lattice cooling through optical/acoustic phonon emissions, making the lattice temperature *T*_L_ increase. Such processes are usually very fast in some 2DMs. For example, in graphene, the electron-heating and electron-cooling processes happen in the time scales of ~50 fs and ~1 ps, respectively^47^. Benefiting from such ultrafast thermal relaxation dynamics, graphene bolometers can work with the bandwidths over tens of GHz, which are very different from their bulk-material counterparts with slow responses in the time scale of millisecond^2,21^.

For the BOL effect, the photo-induced increase of the electron temperature *T*_e_ can be transferred to the electric response via different readouts. For example, three typical bolometer readouts have been employed for graphene bolometers^50^, as shown in Fig. 1h. The *first* one is the thermal-resistance readout^51^. As the electron temperature *T*_e_ increases under illumination, the carrier mobility *μ* decreases according to the relationship *μ*~*T*_e_^−γ^, where the coefficient *γ* is expected to be 1 for graphene in the acoustic phonon limited condition^49^. Accordingly, the 2DM electrical conductivity *σ* decreases. In this case, the photocurrent has an opposite direction to the applied bias voltage. The BOL responsivity is decided by the temperature-dependent conductivity ∆*σ*/∆*T*_e_, which is large at high doping level and low temperature^52^. The *second* one is Johnson noise thermometry readout^53^. As the electron temperature *T*_e_ increases, the Johnson noise power increases and can be extracted by using microwave circuits. The *third* one is the thermomechanics readout^54^. As the electron temperature *T*_L_ increases, the thermomechanical stress arises and leads to the resonance frequency shift of a mechanical resonator, which can be measured by applying a frequency-sweeping alternating voltage. In addition, graphene-superconductor junctions^55^ and superconducting tunnel junctions^56^ have also been available as possible alternatives. Generally speaking, for graphene bolometers, low-temperature operation is helpful to achieve competitive performances^50^.

For the PTE effect, which is the dominated mechanism for lateral *p*-*n* configured graphene PDs^57^, the electron temperature *T*_e_ increases with light illumination and the local photo-thermal-voltage generates through the Seebeck effect (see Fig. 1i). A non-zero channel- integral photo-thermal-voltage arises when either the Seebeck coefficient distribution or the electron temperature distribution is asymmetric. The photo-thermal voltage can drive the photocurrent without a bias voltage. The detailed processes for the PTE effect have been introduced in many works^1,5,6,28,36^. Even though the PDs based on the PTE effect can work fast, the responsivity is usually not high unless there are special structures for optical enhancement^58^. Besides, one should note that the bias voltage-insensitive PTE photoresponse usually becomes negligible compared to other photoconductive photoresponses in the PDs when bias voltages are applied^58^.

### Device configurations of 2DM PDs

**Fig. 2:**
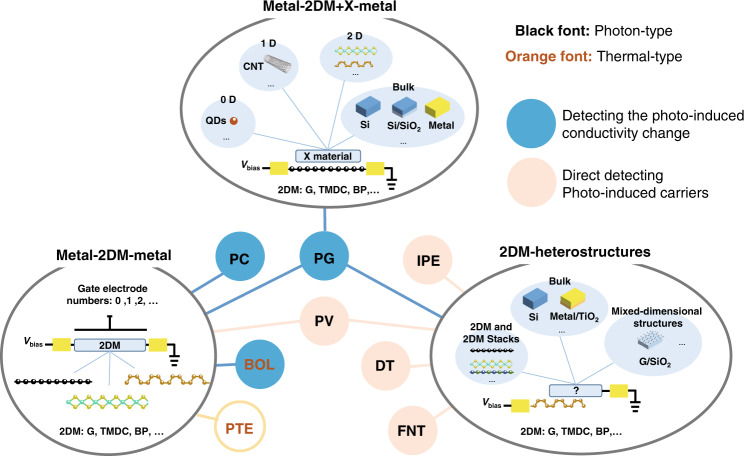
The relation between the working mechanisms and the configurations of the 2DM PDs. Both metal-2DM-metal and metal-2DM+X-metal configurations have a phototransistor structure featuring a 2DM channel. The former has a pure 2DM channel, while the latter has a 2DM channel contacted with another specific material “X”, such as zero-dimensional (0D) quantum dots, 1D carbon nanotubes (CNTs), 2DMs, and even bulk materials. In the 2DM-heterostructure configuration, the electrodes are connected to different materials. PV photovoltaic, IPE internal photon emission, DT direct tunneling, F-N tunneling Fowler-Nordheim (F-N) tunneling, PC photoconductive, PG Photo-gating, BOL bolometric, PTE photo-thermoelectric, QDs quantum dots, CNT carbon nanotubes.

Since the first 2DM PD was demonstrated with a simple metal-graphene-metal (M-G-M) configuration in 2009^59^, lots of 2DM PDs have been proposed and demonstrated^1,2,5,6,28,36^, which is attributed partially to the flexibility of 2DMs. In this part, the device configurations for 2DM PDs are summarized and discussed. More importantly, the connection between the photodetection mechanisms and the device configurations is revealed with the configurations classified to three categories, i.e., the metal-2DM-metal type, the metal-2DM+X-metal type, and the 2DM-heterostructure type, as shown in Fig. 2.

As the most fundamental configuration, the metal-2DM-metal type usually has one or two gate electrodes for manipulating the 2DM doping properties, as shown in the left-bottom part of Fig. 2. Even for such a simple PD, there are usually multiple photodetection mechanisms involved. For example, for graphene phototransistors operating at a near-zero/zero-bias voltage, the PTE effect is the dominant contribution to the photoresponse in the laterally asymmetric channel^60^, the lateral *p*-*n* junction^57,61^, as well as the metal-graphene interface^58,62^. When the applied bias voltage increases, the PC-type effects with much higher responsivities than the PTE effect become dominant^58,63^. In addition, the PC effect and the BOL effect with contrary photocurrents compete, while the doping level in graphene is the key^58,63^. When the graphene doping level is lower, the longer carrier life time makes the PC effect stronger, meanwhile the BOL effect is weaker since the carrier mobility is less sensitive to temperature in this case^52^, and thus the PC effect dominates, and vice versa^58,63^.

In contrast, TMDC- and BP-PDs have different mechanisms from their graphene counterparts. In TMDC or BP *p*-*n* junctions formed by two separated gate electrodes, the photocurrents originating from the PV effect were observed^64,65^. For a regular single-gate phototransistor, it has been reported that all the PC-, PG-, BOL-, and PTE-effects partially contribute to photodetection. In many TMDC or BP phototransistors, there existed some structural defects and thus some trap states at the band tails were introduced. Such devices usually have photoresponses dominated by the PG effect^43,66–69^. On the other hand, in some phototransistors with lightly doped TMDC or BP, the PC effect^43,70^ or the PV effect in the PC mode^67,71,72^ happens, similar to their graphene counterparts. In contrast, the BOL effect was found to be dominant for those phototransistors based on heavily doped BP^71^. Furthermore, the PTE effect and the BOL effect were found to contribute dominantly to the low-bias (<0.5 V) and high-bias photoresponses of BP phototransistors, respectively^49^. According to ref. ^73^, the photoresponse at the BP-metal interface is usually dominated by the PV effect or the PTE effect, depending on the band bending. When the band bending is significant, the PV effect is the dominant. Otherwise, the dominant is the PTE effect.

Figure 2 also shows the PDs based on the metal-2DM+X-metal configuration, which consists of a 2DM channel contacted with another specific material “X”, such as zero-dimensional (0D) quantum dots^74,75^, 1D carbon nanotubes (CNTs)^76^, 2DMs^46,77^, and even bulk materials^45,78–81^. Light absorption may occur in the 2DM channel region^78,80,81^ or the X-material region^45,46,74,76,79^. Typically, the specific material X contacting the 2DM channel plays the role of introducing trap states. In this case, it is usually the PG effect dominating the photoresponse with very high responsivity (e.g., 10–10^10^ A W^−1^)^45,46,74^^,^^76–79^^,^^81^.

When using the 2DM-heterostructure configuration (see Fig. 2), the signal and ground electrodes are respectively connected to the 2DM region and another material(s) region in contact, in which way a 2DM-heterostructure is formed. As shown in Fig. 2, the material(s) in contact with the 2DM can be two-, three-, or mixed-dimensional material(s). In order to realize high-performance PDs, various 2DM-heterostructures have been developed with band engineering and carrier-dynamics manipulations, despite that the fabrication difficulty increases. As demonstrated, the PV effect has been often observed in 2DM-heterostructures of e.g., vertically^82–84^ or laterally overlapped^85^ G-TMDC-G, TMDC-G-TMDC^86^, TMDC-TMDC^87,88^, G-TMDC^89,90^, PTMDC-TMDC^91^, and BP-TMDC^92–95^. In these PDs, light is often absorbed by the non-zero bandgap 2DMs (e.g., TMDC, BP), and then the photo-excited carriers are separated along the bended band which can be manipulated by applying a gate voltage. In order to understand the carrier dynamics in the 2DM-heterostructures, one should usually take the quantum effect into consideration^87,96^. More recently, Gao et al. reported an APD based on an InSe-BP heterojunction, in which carrier-dynamics multiplication was realized with the ballistic avalanche phenomena^97^. The PV effect also happens in 2DM/bulk-material heterostructures, and particularly the G-Si type has attracted the most attention. In G-Si heterostructures, the photo-excited carriers are separated along the bending bands formed in the Schottky junction when light is mainly absorbed in silicon. In contrast, the IPE effect becomes the dominant when light is mainly absorbed by graphene, in which case the hot carriers with sufficient energy are emitted over the G-Si Schottky barrier and then contribute to the photocurrent^98,99^. Similar processes also occur in G-TMDC-G^100^ and G-hBN-G junctions (hBN is hexagonal boron nitride)^101^. For the G-hBN-G junctions operating with high-bias voltages, the photoresponse may also come from the tunneling effects, namely the DT and F-N tunneling^41^. The photoresponse based on the tunneling effects was also achieved in some other 2DM-insultor-2DM/bulk-material configurations, e.g., G-hBN-MoS_2_^42,102^.

In addition, recently Lee et al. reported a broken-gap SnSe_2_-MoTe_2_ heterojunction, which forms an Ezaki diode. In this case, the photoresponse is contributed from both the PV effect and the F-N tunneling^91^. Besides, the PG effect-based photoresponses were also observed in 2DM-heterostructure PDs, such as BP-MoS_2_ heterostructure PDs^92^ and laterally overlapped TMDC-BP-TMDC bipolar phototransistors^93^. In these PDs, there may be trap states originating from the defect in materials or heterostructure interfaces, resulting in the PG effect-based photoresponse. More recently, a graphene-insulator-metal PD was demonstrated with graphene covered by colloidal quantum dots (CQDs), showing an interesting mixed-photoresponse mechanism^103^. In this PD, the charge trapping upon light absorption in the CQDs layer induces a shift of the chemical potential of graphene (which is analogous to the PG effect). Then the photocurrent generates from the change of the current flowing across the graphene-insulator-metal structure, through the F-N tunneling effect and/or the IPE effect.

## Waveguide-integrated silicon-2DM PDs

As is well known, a 2DM layer usually has quite limited absorption for normal-incident light. For example, the absorption ratio is about 2.3% for single-layer graphene. Fortunately, it is possible to strongly enhance the interaction between a 2DM layer and the optical modal field propagating along an optical waveguide by extending the interaction length^104^.

With the great enhancement of light-matter interaction, the waveguide-integrated silicon-2DM PDs have attracted much attention for the potential applications in various functional photonic integrated circuits for e.g., optical communications and interconnects^105^. In this section, recent progresses of the popular waveguide-integrated silicon-2DM PDs are reviewed and summarized, including the metal-2DM-metal PDs in “Metal-2DM-metal PDs” section, as well as the 2DM-heterostructure PDs in “2DM-heterostructure PDs” section.

### Metal-2DM-metal PDs

**Fig. 3:**
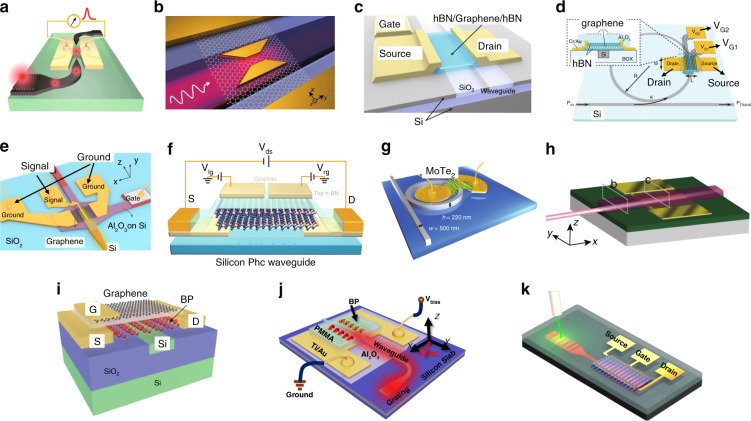
The waveguide-integrated Si/2DM PDs with metal-2DM-metal configurations. **a** A graphene plasmonic PD working with the PV effect. **b** A plasmonically enhanced graphene PD working on the BOL effect. **c** A horizontally asymmetric graphene PD with one gate electrode based on the PTE effect. **d** A microring resonator-integrated two-gate graphene PD based on the PTE effect. **e** The Si-G hybrid plasmonic waveguide PDs operating at 1.55 and 2 μm. **f** A two-gate MoTe_2_ PD operating at 1.16 μm based on the PV effect. **g** A strain-engineered MoTe_2_ PD integrated on a microring resonator operating at 1.55 μm. **h** A PtSe_2_ PD operating at 1.55 μm. **i** A black-phosphorus PD operating at 1.55 μm with 3 GHz bandwidth. **j** A black-phosphorus PD operating at 2 μm. **k** A PG effect-based black phosphorus PD operating at the wavelength band of 3.68–4.03 μm. Figures reproduced with permissions from: **a** ref. ^108^, ^©^2020 De Gruyter, Berlin/Boston, under Creative Commons Attribution 4.0 International license (CC BY 4.0, https://creativecommons.org/licenses/by/4.0/); **b** ref. ^119^, ^©^2018 American Chemical Society; **c** ref. ^60^, ^©^2015 American Chemical Society; **d** ref. ^115^, under Creative Commons Attribution 4.0 International license (CC BY 4.0, https://creativecommons.org/licenses/by/4.0/); **e** ref. ^58^, ^©^2020 Springer Nature Limited, under Creative Commons Attribution 4.0 International license (CC BY 4.0, https://creativecommons.org/licenses/by/4.0/); **f** ref. ^64^^©^2017 Springer Nature Limited. **g** ref. ^70^, ^©^2020 Springer Nature Limited; **h** ref. ^121^, ^©^2020 American Chemical Society; **i** ref. ^71^, ^©^2015 Springer Nature Limited; **j** ref. ^72^, ^©^2019 John Wiley & Sons, Inc. **k** ref. ^68^, ^©^2018 American Chemical Society. Further permissions related to the figures should be directed to the copyright holders.

Figure 3 shows the representative metal-2DM-metal PDs with various materials as well as different mechanisms. Among them, it can be seen that the M-G-M PDs^58,60,106–119^ are very popular because the fabrication process is relatively easy. For M-G-M PDs, the PV effect is hardly observed because the life time of the photo-generated carriers in graphene is very short (~2 ps^120^). When the channel length is reduced to as short as ~100 nm, the PV effect becomes dominant for the photoresponse, which happens when using a metal-air-metal plasmonic waveguide, as shown in Fig. 3a. In this case, an ultra-short channel (~120 nm) and 19-μm-long PD was demonstrated with a responsivity of 360 mA W^−^^1^ at 2.2 V bias voltage as well as a bandwidth of >110 GHz. In contrast, the BOL effect is widely observed in the M-G-M PDs when a bias voltage is applied. For example, Ma et al. presented a 6-μm-long M-G-M PD integrated with bowtie-shaped metallic nanostructures, as shown in Fig. 3b, exhibiting a high external responsivity of 0.5 A W^−^^1^ under −0.4 V bias voltage and a frequency response exceeding 110 GHz.

On the other hand, for M-G-M PDs, typically the resistance is 10^2^–10^3^ Ω, and correspondingly the dark current might reach the level of ~mA even when the bias voltage is below 1 V. In order to reduce the dark current, it is preferred to develop PDs working under near-zero bias with the PTE effect. As demonstrated previously, the PTE photoresponse can be generated by introducing an asymmetric distribution of electron temperature *T*_e_^58,60^ or an asymmetric distribution of chemical doping in 2DMs^112^. Figure 3c shows a representative example of the former case. In such a one-gate configuration, the laterally asymmetric light absorption leads to an asymmetric distribution of electron temperature *T*_e_. The PD was measured with a bandwidth of 42 GHz and a responsivity of 78 mA W^−1^ at zero bias, which is impressive compared to its counterparts at zero bias.

For the case with an asymmetric distribution of chemical doping in 2DMs, the lateral *p*-*n* junction structure constructed with two gate electrodes is a popular design. In such PDs, both the light absorption and the electron temperature *T*_e_ distribute symmetrically. However, the Seebeck coefficients of the left and right half-parts have opposite signs. As a result, the PTE-voltages for the left and right half-parts can be accumulated instead of canceled. In order to enhance the light-graphene interaction, one can integrate the lateral *p*-*n* junction structure with a nano-slot waveguide^112^, a slow-light photonic-crystal waveguide^113^, a plasmonic waveguide^114^, as well as a microring resonator shown in Fig. 3d^115^. In ref. ^115^, the light absorption ratio for the ~6 µm-long single-layer graphene sheet is >90%, and the demonstrated PD has a high zero-bias response of 90 V W^−1^ and a bandwidth of 12 GHz. Recently, silicon-graphene hybrid plasmonic waveguide PDs working on 1.55 µm and beyond were demonstrated, as shown in Fig. 3e. Under a non-zero-bias voltage, the BOL effect and the PC effect were found to dominate the photoresponses in highly and lightly doped graphene, respectively. The demonstrated PD operating at 2 μm has a responsivity of 70 mA W^−1^ at −0.3 V bias voltage and a bandwidth of >20 GHz. When operating at 1.55 μm, the PD has a responsivity of 400 mA W^−1^ at −0.3 V bias voltage and a bandwidth of over 40 GHz. In short, even though M-G-M PDs have shown advantages in high speed, it is still much desired to achieve low dark current, low thermal noise, and high responsivity simultaneously.

As alternatives, the 2DMs with non-zero bandgaps (e.g., TMDC and BP) have also been utilized for realizing metal-2DM-metal PDs with lower dark currents than their graphene counterparts. As shown in Fig. 3f, Bie et al. presented a metal-MoTe_2_-metal PD with a lateral *p*-*n* junction structure, in which the PV effect dominantly contributes to the photoresponse. The responsivity is 4.8 mA W^−1^ at zero bias and the bandwidth is 200 MHz when operating at the wavelength of 1.16 μm (which is slightly shorter than the intrinsic absorption edge wavelength ~1.24 μm for MoTe_2_). Recently, the MoTe_2_ bandgap was modulated successfully through the strain engineering, and a microring resonator-integrated MoTe_2_ PD was demonstrated for the wavelength band of 1.55 μm. The PD exhibited a responsivity of about 0.5 A W^−1^ at −2 V bias, a low dark current of ~13 nA, a noise equivalent power (*NEP*) of 90 pW Hz^−1/2^ and a bandwidth of 35 MHz. More recently, PtSe_2_ has attracted intensive attention as a type of wide-sense TMDC 2DM with high chemical stability in air as well as thick-dependent non-zero-bandgap of 0.1–1.2 eV, which enables a wide operation wavelength range. As shown in Fig. 3h, Wang et al. demonstrated a Si_3_N_4_-waveguide-integrated PtSe_2_ PD based on the bound-states-in-continuum scheme. This PD has a responsivity of ~12 mA W^−1^ at 8 V bias voltage with a dark current of 317 nA and a large bandwidth of 35 GHz^121^.

BP has also been extensively studied for realizing PDs, and waveguide-integrated BP PDs have been demonstrated with the PV effect, as shown in Fig. 3i^39^. The demonstrated PD with a 11.5-nm-thick BP sheet has an extrinsic responsivity of 19 mA W^−1^, a low dark current of 220 nA, and a large bandwidth of 3 GHz at 1550 nm. A 3 Gbit s^−1^ data transmission was also performed with a low optical power of 1.2 mW. Later, silicon-BP hybrid waveguide PDs were realized for the wavelength band of 2 μm, as illustrated in Fig. 3j^71^. These PDs performed responsivities of 21–307 mA W^−1^ at 0.4 V bias voltage and bandwidths of 0.5–1.33 GHz, meanwhile the data transmission with a bit rate of 4.0 Gbit s^−1^ was demonstrated successfully. In Fig. 3k, Huang et al. reported waveguide-integrated BP PDs operating at 3.68–4.03 μm, in which the PG effect plays a major role and results in high-gain photoresponses^68^. When operating at a bias of 1 V, the BP PDs have responsivities of 23 A W^−1^ and 2 A W^−1^ for the wavelengths of 3.68 μm and 4 μm, respectively. Meanwhile, the *NEP*s were measured to be ~ 0.01–1 nW Hz^−1/2^ at room temperature. Compared to the M-G-M PDs, the metal-TMDC-metal (M-TMDC-M) and metal-BP-metal (M-BP-M) PDs have smaller bandwidths, much lower dark currents, and comparable responsivities unless the PG effect dominates.

### 2DM-heterostructure PDs

**Fig. 4:**
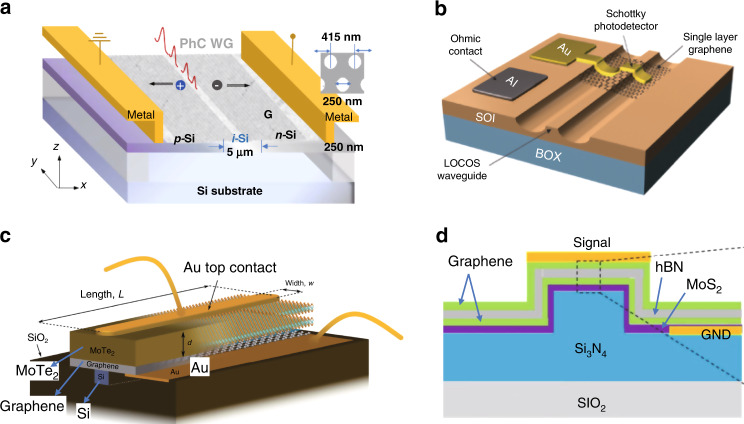
The waveguide-integrated Si/2DM PDs with heterostructure configurations. **a** A high-speed G-Si PD with p-i-n doping distributions for both Si and graphene. **b** A Si-G plasmonic Schottky photodetector. **c** A MoTe_2_-G heterostructure PD. **d** A G-hBN-G heterostructure PD. Figures reproduced with permissions from: **a** ref. ^122^, ^©^2018 Springer Nature Limited, under Creative Commons Attribution 4.0 International license (CC BY 4.0, https://creativecommons.org/licenses/by/4.0/); **b** ref. ^99^, ^©^2016 American Chemical Society; **c** ref. ^90^, ^©^2020 Springer Nature Limited; **d** ref. ^123^; ^©^2019 The Optical Society, under Creative Commons Attribution 4.0 International license (CC BY 4.0, https://creativecommons.org/licenses/by/4.0/). Further permissions related to the figures should be directed to the copyright holders.

In recent years, waveguide-integrated 2D-Heterostructure PDs have attracted intensive attention and several representative examples are shown in Fig. 4. The G-Si heterostructure is one of the most widely used configurations, and most G-Si PDs have limited bandwidths (<MHz) due to the long transit time in silicon. Fortunately, it is possible to achieve very fast PDs with the help of silicon doping engineering. For example, Li et al. demonstrated a high-speed waveguide- integrated G-Si *p*-*i*-*n* photodiode by utilizing the IPE effect, as depicted in Fig. 4a, in which fast carrier transit in silicon is enabled by silicon doping engineering. When operating at 1550 nm, the fabricated PD exhibited a relatively low responsivity of 11 mA W^−1^ under zero bias and >50 GHz RF-limited bandwidth. In particular, the G-Si Schottky junction helps suppress the thermal noise, while the dark current becomes zero for zero-bias operation. Therefore, more than 50 dB converted signal-to-noise ratio (SNR) was achieved even at 40 GHz^122^. In order to achieve an improved responsivity, a metal-G-Si PD was reported with an interesting configuration, in which a graphene sheet was introduced to work with a conventional metal-Si Schottky PD, as shown in Fig. 4b. When this PD operating at 1 V reverse bias, the responsivity is about 85 mA W^−1^, which is one order of magnitude higher than metal-Si Schottky PDs^99^.

The 2DM-heterostructures have also been integrated with silicon photonic waveguides for on-chip photodetection. As shown in Fig. 4c, a 33-μm-long waveguide-integrated MoTe_2_-G heterostructure PD was demonstrated with an external responsivity of 0.2 A W^−1^ under −3V bias voltage at 1300 nm^90^. Here the vertically stacked structure design minimizes the path length of the carrier transit in TMDCs, which contributes to achieve a bandwidth as high as ~24 GHz. For this PD, the normalized photocurrent to the dark current ratio (*NPDR*) is 100–1000 mW^−1^ under a bias voltage of −0.5–0 V^90^. More recently, a Si_3_N_4_ waveguide-integrated G-hBN-G heterostructure tunneling photodiode was demonstrated with a responsivity of ~0.24 A W^−1^ at ~1550 nm, as shown in Fig. 4d. This PD has a bandwidth of 28 GHz and a good *NPDR* of 10^4^–10^5^ mW^−1^ under 0.5–10 V bias voltage, benefiting from the dark current suppression by the tunneling barrier^123^. It can be seen that the PDs with 2DM-heterostructures have great potentials for realizing high sensitivity and broad bandwidth simultaneously.

### Summary for waveguide-integrated 2DM PDs on silicon

Table 1 gives a summary for the performances of waveguide-integrated 2DM PDs on silicon. It can be seen that currently most works focus on the wavelength band of 1550 nm. Among them, the M-G-M PDs provide an interesting option enabling bandwidths even as high as 110 GHz^108,119^. On the other hand, the *p*-*n* graphene homojunction based on the PTE effect is an attractive option because zero-bias operation can be achieved without any dark current shot noise. The state-of-the-art zero-bias responsivity has reached 90 V W^−1^, enabling high sensitivity comparable to the counterpart based on the mature semiconductor technology^115^. In contrast, the PDs based on the BOL effect can realize high responsivity at moderately low-bias voltages as well as low input optical power, for which unfortunately the photodetection linearity still needs much improvement^58,119^. For the M-TMDC-M and M-BP-M PDs based on the PC or PV effects, the responsivities are similar while the bandwidths are small when compared to M-G-M PDs. For those PDs based on the PG effect, the responsivities can be up to ~10 A W^−1^ in cost of small bandwidths^69^. Currently, the 2DM-heterostructure configuration was recognized as one of the most promising options because the device responsivity are comparable to that of the metal-2DM-metal PDs (~10–10^2^ mA W^−1^), meanwhile the noise can be suppressed greatly with the help of the junction structures^89,90,98,99,122,123^.

**Table 1:** Summary of waveguide-integrated Si/2DM PDs

Structure	*λ*	Mechanism	Responsivity	|Bias voltage|	Bandwidth^a^	Refs.
M-G-M	~1.35 μm	PV	0.2 A W^−1^	0.5 V	–	^106^
		BOL	0.67 A W^−1^			
M-G-M	~1.55 μm	PV	7–50^b^ mA W^−1^	0 V	3–110 GHz	^107–111^
			57–108 mA W^−1^	1 V		
			360 mA W^−^^1^	2.2 V		
M-G-M	~1.55 μm	PTE	35–78 mA W^−1^	0 V	12–67 GHz	^60,112–117^
			3.5–90 V W^−1^			
M-G-M	~1.55 μm	BOL	90–500 mA W^−1^	0.3–0.4 V	40–110 GHz	^58,119^
M-G-M	~2 μm	BOL	45–70 mA W^−1^	0.3 V	20 GHz	^58^
M-G-M	~3.8 μm	No stated	2.2 mA W^−1^	1 V	–	^160^
M-MoTe_2_-M	~1.16 μm	PV	4.8 mA W^−1^	0 V	200 MHz	^64^
M-MoTe_2_-M	~1.55 μm	PC	468 mA W^−1^	2 V	35 MHz	^70^
M-PtSe_2_-M	~1.55 μm	PC	12 mA W^−1^	8 V	35 GHz	^121^
M-BP-M	~1.55 μm	PV	135–657 mA W^−1^	0.4–2 V	3 GHz	^71^
M-BP-M	~1.55 μm	PC	6.25 A W^−1^	0.7 V	150 MHz	^161^
M-BP-M	3.68 μm	PG	0.7–23 A W^−1^	1 V	–	^68^
	4 μm		0.5–2 A W^−1^			
M-BP-M	~3.825 μm	PG	0.1–11.31 A W^−1^	0.5 V	550 Hz	^69^
M-BP-M	2 μm	PV	0.026–0.307 A W^−1^	0.4 V	0.5–1.33 GHz	^72^
MoTe_2_-G	~1.31 μm	PV; PC	23–400 mA W^−1^	3 V	0.5 GHz	^89^
MoTe_2_-G	1.26–1.34 μm	PV; PC	~ 7–150 mA W^−1^	0.6 V	12–46 GHz	^90^
G-hBN-G	~1.55 μm	DT; F-N tunneling	240 mA W^−1^	10 V	28 GHz	^123^
G-Si	2.75 μm	IPE	0.13 A W^−1^	1.5 V	–	^98^
Au-G-Si	~1.55 μm	IPE	85 mA W^−1^	1 V	–	^99^
G-Si	~1.55 μm	IPE	11 mA W^−1^	0 V	>50 GHz	^122^

*PV* photovoltaic, *IPE* internal photon emission, *DT* direct tunneling, *F-N* *tunneling*, Fowler-Nordheim (F-N) tunneling, *PC* photoconductive, *PG* Photo-gating, *BOL* bolometric, *PTE* photo-thermoelectric.

^a^The measured bandwidths may be setup limited.

^b^In ref. ^107^, the graphene has two or three layers.

**Fig. 5:**
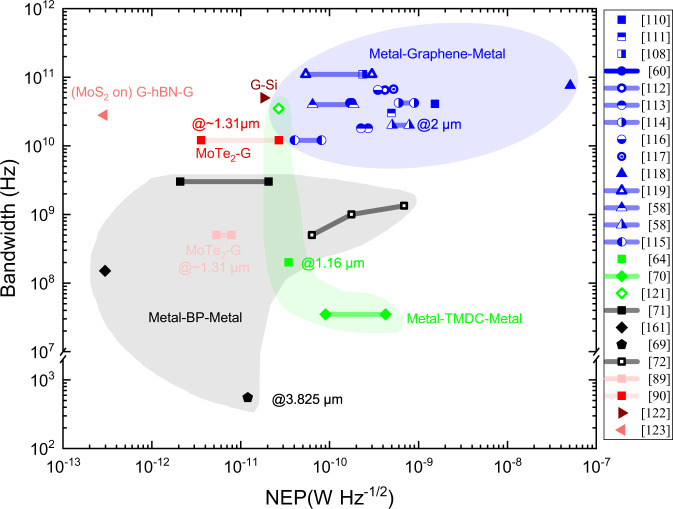
Performance summary for the waveguide-integrated Si/2DM PDs. ■: photovoltaic (PV) effect, ●: photo-thermoelectric (PTE) effect, ▲: bolometric (BOL) effect, ♦: photoconductive (PC) effect, ⬟: Photo-gating (PG) effect, ◄: tunneling effects, ►: internal photon emission.(IPE) effect; blue: metal-graphene-metal (M-G-M); green: metal-TMDC-metal; black: metal-BP-metal; red: 2DM-heterostructure

In order to further give a comprehensive comparison on the PD performances, a summary map is given in Fig. 5, where the *x*-axis and *y*-axis, respectively, denote the *NEP* and the bandwidth. Here the *NEP* is calculated by considering the dark current shot noise and the thermal noise, as discussed in Supplementary Note 1.3. As depicted in Fig. 5, the M-G-M PDs have unique advantages in the scenarios when ultra-large bandwidths (~100 GHz) are required. However, the PD sensitivity is still quite limited. In contrast, better *NEP* can usually be achieved for M-TMDC-M or M-BP-M PDs. A promising option is using 2DM-heterostructures, which might enable a large bandwidth of ~50 GHz and a high sensitivity of ~10^−13^–10^−11^ W Hz^−1/2^.

## Surface-illuminated Si/2DM PDs

In this section, we give a review for the recent progresses of surface-illuminated Si/2DM PDs, which have potentially important applications in free-space optical communication and optical imaging. Here we focus on the devices working at NIR and MIR wavelengths, including the metal-2DM-metal PDs and metal-2DM+X-metal PDs in “Metal-2DM-metal and metal-2DM+X-metal PDs” section as well as the 2DM-heterostructure PDs in “2DM-heterostructure PDs” section. Furthermore, “PDs and arrays for image sensing” section gives a summary for the reported image-sensor array chips^124,125^ and proof-of-concept image systems^126,127^ based on surface-illuminated 2DM PDs. Finally, a comprehensive summary for these PDs is given in “Summary of surface-illuminated 2DM PDs on silicon” section.

### Metal-2DM-metal and metal-2DM+X-metal PDs

**Fig. 6:**
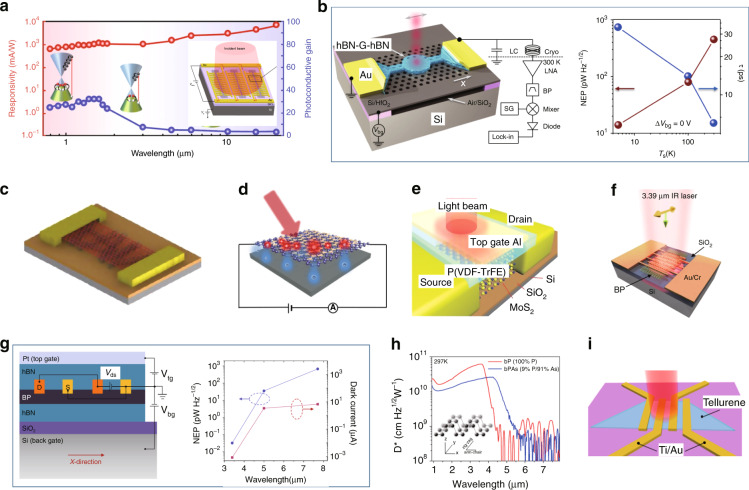
The surface-illuminated Si/2DM PDs with metal-2DM-metal configuration. **a** A wide-band plasmonic enhanced graphene PD and the measured responsivity/photoconductive-gain. **b** A cavity-coupled graphene bolometer with Johnson noise read-out. Left: the 3D schematic. Right: the *NEP* and thermal relaxation time of hot electrons as a function of lattice temperature. **c** A metal-graphene+X-metal configuration PD, for which X is carbon nanotube. **d** A short-wave infrared graphene PD with a plasmonic enhanced structure on channel. **e** A ferroelectric polarization gating MoS_2_ photodetector with an operation wavelength extended to 1.55 μm. **f** A mid-infrared black-phosphorus PD with a high gain. **g** A mid-infrared black-phosphorus PD with an operation wavelength extended to 7.7 μm by applying a vertical electric field. Left: the 3D schematic. Right: the *NEP* and dark current at different wavelengths. **h** The specific detectivities of the mid-infrared black phosphorus PD and black-PAs-alloy PD as a function of wavelength. **i** A short-wave infrared tellurene PD. Figures reproduced with permissions from: **a** ref. ^128^, ^©^2018 Springer Nature Limited, under Creative Commons Attribution 4.0 International license (CC BY 4.0, https://creativecommons.org/licenses/by/4.0/); **b** ref. ^53^, ^©^2018 Springer Nature Limited; **c** ref. ^76^, ^©^2015 Springer Nature Limited, under Creative Commons Attribution 4.0 International license (CC BY 4.0, https://creativecommons.org/licenses/by/4.0/); **d** ref. ^78^, ^©^2017 American Chemical Society; **e** ref. ^81^. ^©^2015 John Wiley & Sons, Inc. **f** ref. ^67^, ^©^2016 American Chemical Society; **g** ref. ^29^, ^©^2017 Springer Nature Limited, under Creative Commons Attribution 4.0 International license (CC BY 4.0, https://creativecommons.org/licenses/by/4.0/); **h** ref. ^129^, ^©^2017 American Chemical Society. **i** ref. ^131^, ^©^2019 American Chemical Society. Further permissions related to the figures should be directed to the copyright holders.

Figure 6 shows several representative surface-illuminated metal-2DM-metal and metal-2DM+X-metal PDs. As seen in Fig. 6a, a graphene PD using engineered nanostructures based on gold-patched graphene nano-strips was demonstrated with high PC gain, benefiting from the reduction of photocarrier transport time through the electrodes. This device enables broadband photodetection from the visible to the infrared regime. The responsivities were measured to be 0.6 A W^−1^ at 0.8 μm and 11.5 A W^−1^ at 20 μm, while a large bandwidth of over 50 GHz was measured at 0.8 μm. Unfortunately, the photocurrent saturates when the input power increases to only ~10 μW (at 0.8 μm), which may be attributed to the limited active region areas and the density of states in graphene^128^.

Figure 6b shows a hot-electron graphene bolometer based on Johnson noise read-out at 1531 nm^53^. As is well known, the enhancement of the light-2DM interaction is really important for surface-illuminated PDs due to the ultra-thin light absorption layer. As shown in Fig. 6b, a suspended silicon photonic-crystal cavity enables 45% light absorption in graphene at the resonance wavelength. This device has a NEP of ~10 pW Hz^−1/2^ and fast thermal relaxation less than 35 ps at 5 K (see Fig. 6b), which means that the bolometer has a large intrinsic bandwidth. In practice, the bandwidth is usually limited by the readout electronics.

Recently CNTs were introduced for realizing a metal-graphene+CNT-metal PD working at 400–1550 nm, as shown in Fig. 6c. This PD based on the PG effect has a peak responsivity of over 100 AW^−1^, benefiting from high gain which however leads to a slow response time (~100 μs)^76^. Figure 6d shows another PD with the same type of configuration and the same mechanism (i.e., the PG effect). In this configuration, the graphene channel is sandwiched by Au-nanoparticles and the silicon substrate, while the plasmonic effect helps realize photon trapping and enhance light absorption in graphene. With such a design, this PD performed a high responsivity of 83 A W^−1^ at 1550 nm and a response time of less 600 ns, which is outstanding among the PDs based on the PG effect^78^. As an alternative, the surface-illuminated M-TMDC-M PDs are also very popular^6^ and most of them were demonstrated for visible light regarding the material bandgaps. On the other hand, it is still possible to extend the working wavelength range by utilizing some special approaches. For example, a ferroelectric-polymer-film-gated triple-layer MoS_2_ PD was demonstrated to work at 1550 nm, utilizing the ferroelectric polarization tuning for the bandgap of few-layer MoS_2_ (see Fig. 6e)^81^.

BP is another promising candidate for NIR and MIR photodetection. As depicted in Fig. 6f, a M-BP-M PD at 3.39 μm was demonstrated. In this work, the dominated mechanisms are respectively the PV effect and the PG effect for the cases of intrinsic BP and moderately doped BP^67^. A BP PD can even work at wavelength as long as 7.7 μm by introducing a vertical electric field due to Stark effect with help of top and bottom gates (see Fig. 6g)^29^. For this PD, the *NEP*s under a bias voltage of 1.2 V are 0.03, 35, and 672 pW Hz^−1/2^ for the wavelengths of 3.4, 5, and 7.7 μm, respectively, when operating with the corresponding optimal gate voltages. The corresponding dark currents are 8.6 × 10^−4^, 3.42, and 6.75 μA, as shown in Fig. 6g. The operation wavelengths of BP PDs can also be extended by chemical doping. For example, Amani et al. demonstrated two gate-photoconductors based on BP and black-PAs-alloy (b-PAs). The cut-off wavelength can be extended from 3.9 to 4.6 μm when replacing BP by b-PAs, as shown in Fig. 6h. These two PDs have peak specific detectivities of 6 × 10^10^ cm Hz^1/2^ W^−1^ and 2.4 × 10^10^ cm Hz^1/2^ W^−1^ at room temperature, respectively^129^. For another b-PAs phototransistor demonstrated in ref. ^130^, the detection wavelength was extended to 8.05 μm. The 2DM tellurene has also been applied as a new photodetection material^33,131^. In ref. ^33^, a Fabry-Perot (F-P) cavity-integrated metal-Te-metal PD was demonstrated at 1–3.4 μm, showing an optimized room-temperature specific detectivity of 2 × 10^9^ Jones at 1.7 μm. Later, another metal-Te-metal PD (shown in Fig. 6i) was reported with a peak extrinsic responsivity of 383 A W^−1^, 19.2, and 18.9 mA W^−1^ at the wavelengths of 520 nm, 1.55, and 3.39 μm, respectively. In particular, this PD has a bandwidth of 37 MHz when operating at 1.55 μm^131^.

### 2DM-heterostructure PDs

**Fig. 7:**
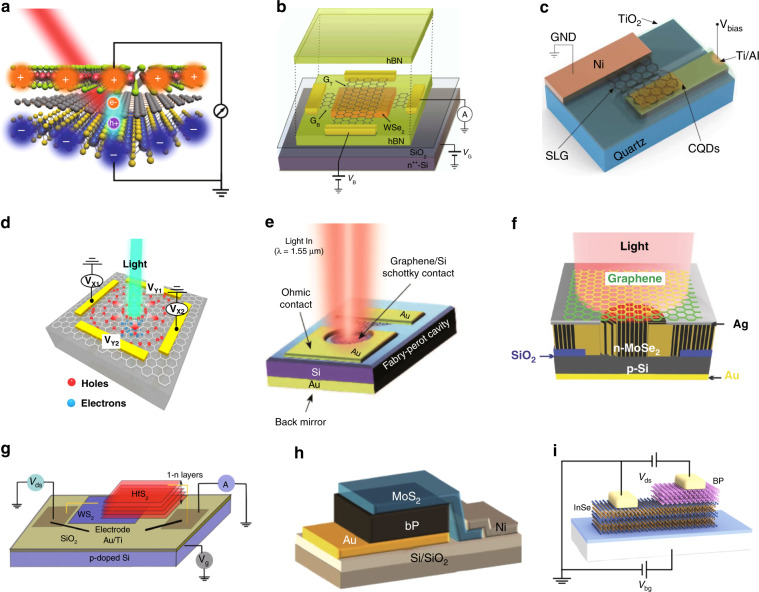
Surface-illuminated Si/2DM PDs with heterostructure configurations. **a** A MoS_2_-G-WSe_2_ PD. **b** A G-WSe_2_-G PD with the IPE effect .**c** A colloidal quantum dot-graphene (CQD-G) hybrid PD with tunneling layer. **d** A G-Si heterostructure position-sensitive PD operating at near-infrared wavelengths. **e** A G-Si PD operating at 1.55 μm. **f** A G/vertical-MoSe_2_/Si heterojunction PD. **g** A mid-infrared WS_2_-HfS_2_ heterostructure PD based on interlayer excitons. **h** A mid-infrared BP-MoS_2_ heterostructure PD. **i** A mid-infrared BP-InSe avalanche photodetector. Figures reproduced with permissions from: **a** ref. ^86^, ^©^2016 American Chemical Society; **b** ref. ^100^, ^©^2016 Springer Nature Limited, under Creative Commons Attribution 4.0 International license (CC BY 4.0, https://creativecommons.org/licenses/by/4.0/); **c** ref. ^103^, ^©^2020 American Chemical Society; **d** ref. ^135^, ^©^2018 The Optical Society, under Creative Commons Attribution 4.0 International license (CC BY 4.0, https://creativecommons.org/licenses/by/4.0/); **e** ref. ^136^, ^©^2017 American Chemical Society; **f** ref. ^139^, ^©^2016 John Wiley & Sons, Inc, under Creative Commons Attribution 4.0 International license (CC BY 4.0, https://creativecommons.org/licenses/by/4.0/); **g** ref. ^13^^©^2020 Springer Nature Limited. **h** ref. ^94^, ^©^2018 Springer Nature Limited; **i** ref. ^97^, ^©^2019 Springer Nature Limited. Further permissions related to the figures should be directed to the copyright holders.

Various 2DM-heterostructure PDs have been developed in recent years. As depicted in Fig. 7a, Long et al. presented a PD based on the PV effect by using the MoS_2_-G-WSe_2_ heterostructure^86^. This vdW heterostructure PD enables the photodetection in a broadband wavelength range from 400 to 2400 nm. Its specific detectivity is 10^9^–10^10^ Jones at the NIR and MIR wavelength bands, while the bandwidth is ~6.5 kHz estimated from the time-dependent photoresponse at 637 nm. Figure 7b shows a G-WSe_2_-G heterostructure PD^100^. When this PD operates beyond the absorption band for WSe_2_, light absorption in graphene is the key and the responsivity based on the IPE effect is 0.12 mA W^−1^, corresponding to an IQE of 2%. Meanwhile, the IPE effect is very fast with a charge injection time of ~47 ps^100^. The photoresponse can also be very fast in the time scale of picosecond when light absorption in WSe_2_ is the dominant at the wavelength band of <950 nm^84^. Recently, a CQD-G heterostructure PD was reported, as shown in Fig. 7c. For this PD, light is absorbed by the CQDs on graphene. Then the charge trapping in the CQDs induces a shift of the chemical potential in graphene, resulting in the change of the current flowing across the G-insulator-metal structure. In such a PD, the dark current is usually several μA or even sub-μA, which is much lower than that of conventional metal-2DM-metal PDs based on the PG effect. Meanwhile, the PD has a high gain leading to a high responsivity of 70 A W^−1^ at 1625 nm as well as excellent response linearity for the input power density less than 1.1 W m^−2^^103^.

As another popular configuration, the G-Si heterostructure has also been exploited widely for realizing surface-illuminated PDs for visible^132–135^, NIR^135–137^, and MIR^138^ light. As shown in Fig. 7d, a position-sensitive PD was demonstrated with a G-Si heterostructure, where graphene plays the role of the photon-absorbing and charge-separation layer^135^. This PD has excellent position sensitivity to weak light at nanowatt level, nanosecond-scale high response speed, as well as low response nonlinearity in the NIR region. As is well known, the PD response usually can be enhanced by introducing an optical cavity^136^, for instance, the F-P cavity in Fig. 7e. In this case, light is absorbed in graphene and the photocurrent can be generated with an improved external responsivity of ~20 mA W^−1^ through the IPE effect. As shown in Fig. 7f, a more complicated heterojunction PD with a G/vertical-MoSe_2_/Si structure was demonstrated at the wavelength band of 350–1310 nm^139^. The presence of graphene enables the photodetection at the wavelength beyond the absorption edges of MoSe_2_ and Si. Such a PD has a strong built-in electric field and a short transmit time, guaranteeing a fast response.

More recently, the TMDC-based heterostructures have become very popular for realizing PDs. Note that the TMDCs usually have absorption edges around ~1.24 μm or below. However, the operation wavelengths of the heterostructures can be modified. For example, Lukman et al. demonstrated a WS_2_-HfS_2_ heterostructure MIR PD, as shown in Fig. 7g. Here, the interlayer excitons absorption is strongly enhanced due to the unique band alignment and the orbital hybridization, which thus contributes to the peak responsivity of 9.5 × 10^2^ A W^−1^ at the wavelength of 4.3 μm^13^. In addition, a multi-operation-mode PD with a MoTe_2_-VO_2_ heterostructure on sapphire was demonstrated^140^. It works based on the PV effect in the wavelength range from 450 nm to 2 μm, while the dark current is as low as ~0.2 pA and the response time is about 17 μs. Furthermore, this PD can also work for the wavelength range from 2 to >10 μm when operating with the BOL effect.

As a 2DM with a relatively narrow bandgap, BP has also been combined with TMDCs to form 2DM-heterostructure PDs^92–95^. For example, a BP-MoS_2_ heterostructure PD (shown in Fig. 7h) was reported with a response time of 3.7 μs as well as a high room-temperature specific detectivity of 1.1 × 10^10^ Jones under zero bias at 3.8 μm^94^. Another BP-MoS_2_ heterostructure PD using an F-P cavity for light absorption enhancement was demonstrated with a specific detectivity of 1.7 × 10^9^ Jones at 3.0 μm and a fast response time of less than 3 ns^95^. More recently, a sub-mean-free-path-scaled vertical BP-InSe heterostructure was reported to realize a ballistic APD enabling low threshold (<1 V) and sensitive MIR photodetection at 4 μm^97^.

### PDs and arrays for image sensing

**Fig. 8:**
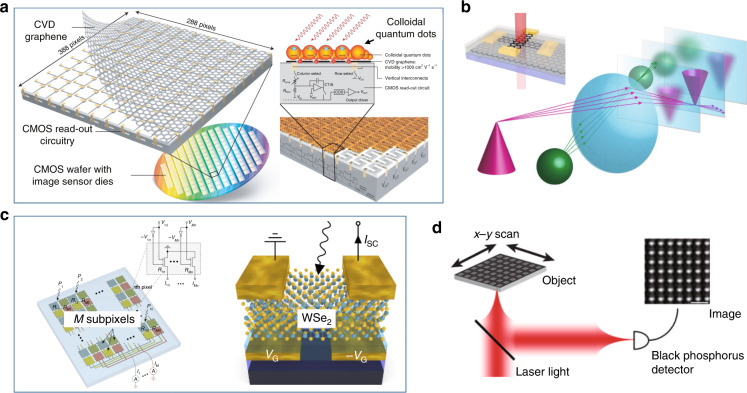
The image sensors based on Si/2DM PDs. **a** An image sensor array based on graphene-CMOS integration, covering ultraviolet, visible and infrared light. Left: the schematic of the graphene transfer process on wafer. Right: the schematic of the graphene/colloidal quantum dot PDs integrated with CMOS read-outs. **b** Concept of a focal stack light-field imaging system using graphene transparent PDs (inset). **c** An artificial neural network image-sensor based on a reconfigurable WSe_2_ PD array. Left: the schematic of the PD array. Right: the schematic of the single-element WSe_2_ PD. **d** A multispectral imaging system based on a black phosphorus PD. Figures reproduced with permissions from: **a** ref. ^124^, ^©^2017 Springer Nature Limited; **b** ref. ^126^, ^©^2020 Springer Nature Limited; **c** ref. ^125^, ^©^2020 Springer Nature Limited; **d** ref. ^127^, ^©^2014 American Chemical Society. Further permissions related to the figures should be directed to the copyright holders.

Surface-illuminated 2DM PDs have been developed further to realize image-sensor-array chips and the proof-of-concept image systems. As shown in Fig. 8a, the demonstrated image sensor consists of a 388 × 288 G-CQD PD array, which can be used for high-sensitivity digital cameras with a broad wavelength range of 300–2000 nm^124^. Each G-CQD PD element given in Fig. 8a shows a high responsivity of >10^7^ A W^−1^ and a high-specific detectivity of >10^12^ Jones. Compared to commercial imaging systems, such an image-sensor array enables the operation with high frame-rates as well as high detection sensitivity even at very wide operation wavelength bands.

Transparent graphene PDs are also useful for a ranging and light-field imaging system, as shown in Fig. 8b^126^. A proof-of-concept single-pixel focal stack light-field camera was developed for depth ranging. A neural network photoelectric image-sensor array with a two-gate metal-WSe_2_-metal PD element was also proposed and developed, as shown in Fig. 8c. The image sensor can realize the acquisition and processing of optical signals simultaneously without converting optical images into digital information formats, enabling real-time image acquisition in the scale of nanoseconds. The image sensor was developed further to realize two types of artificial neural networks, i.e., the image classifier (supervised learning) and image encoder (unsupervised learning)^125^. In ref. ^127^, a multispectral imaging system was demonstrated by deploying a BP PD as a point-like PD in a confocal microscope setup, as shown in Fig. 8d. The BP PD can acquire high-visibility images with submicron resolutions for both visible (e.g., *λ* = 532 nm) and infrared (e.g., *λ* = 1550 nm) regimes, and the corresponding responsivities are 20 and 5 mA W^−1^, respectively.

### Summary of surface-illuminated 2DM PDs on silicon

Table 2 gives a summary for the reported surface-illuminated 2DM PDs on silicon, including the FOMs of the responsivity, the bandwidth/response-time, and the specific detectivity. One can see that the PD performances are usually wavelength-sensitive (e.g., in refs. ^29,134^). Specifically, the linear dynamic ranges are usually not given for those 2DM PDs reported previously. Instead, the responsivities are often given with the measured input power or the power range when possible to partially reflect the linearity properties. Among them, the earliest surface-illuminated M-G-M PDs demonstrated in refs. ^59,141^. have insufficient sensitivity (e.g., 10^5^ Jones in ref. ^141^) due to the limited light absorptions and the intrinsic noise. The M-G-M bolometers reported recently can achieve high theoretical bandwidths and improved specific detectivities at low temperature (e.g., ~1 × 10^7^ Jones^53^). A M-G-M PD with an ultra-short graphene channel achieved a high PC gain and a specific detectivity of 1.5–15 × 10^8^ Jones only on the condition of low input optical power^128^. This feature is similar to those PDs based on the PG effect except the much higher bandwidth of 50 GHz at the wavelength of 0.8 μm. As shown in “Metal-2DM-metal PDs” and “Metal-2DM-metal and metal-2DM+X-metal PDs” sections, the PG effect is observed rarely in M-G-M PDs, except the PD with graphene quantum dot-like arrays^142^, in which the electron trapping centers were introduced and the bandgap in graphene was created through band-structure engineering. In contrast, there are a few M-BP-M PDs reported with the PG effect^67,143,144^. As shown in Table 2, the surface-illuminated M-BP-M PDs have specific detectivities in scale of 10^5^–10^10^ Jones, while the bandwidth is usually below MHz. More recently, the M-Te-M and M-PtSe_2_-M PDs were also demonstrated with high sensitivity and high speed in potential^33,35,131^. For example, the PD reported in ref. ^35^ has a specific detectivity of about 1.2 × 10^7^ Jones and a bandwidth of ~17 GHz.

**Table 2:** Performances of surface-illuminated Si/2DM PDs at the NIR and MIR range.

Configuration	Year	Structure	*λ* (μm)	Mechanism	Responsivity @ input power (*λ*)	|Bias|	Bandwidth/Response time^a^	D* (Jones) @T^b^	Refs.
Metal-2DM-metal	2009	M-G-M	~1.55	PV	0.5 mA W^−1^	–	>40 GHz	–	^59^
	2010	M-G-M	~1.55	PV	6.1 mA W^−1^@0.4 V	–	16 GHz	~1.08 × 10^5c^	^141^
	2018	M-G-M	0.8–20	PV PC	0.6–0.075 A W^−1^ @2.5–50 μW (0.8 μm)	0.02 V	50 GHz (0.8 μm)	~1.5–15 × 10^8^ (3–20 μm)^*^	^128^
					11.5 A W^−1^ @2.5 μW (20 μm)				
	2013	M-GQDs-M	0.532–10.3	PG	0.2–1.25 A W^−1^ (0.53 μm)	0.02 V	–	–	^142^
	2012	M-G(bilayer)-M	0.658–10.6	BOL	2 × 10^5^ V W^−1^ (10.6 μm)	–	>1 GHz (1.03 μm)	~3.03 × 10^10^ (10.6 μm) @5 K^*^	^51d^
	2018	M-G-M	1.531	BOL	–	–	30 ps @5 K (read out-limited)	~ 3.5 × 10^7^@5 K	^53^
	2020	M-G-M	3.4–12	BOL	1.4–5.1 mA W^−1^	0.5 V	47 MHz	~7.22 × 10^4^–2.65 × 10^5*^	^162^
	2017	M-BP-M	2.5–3.7	PV; PC	160 mA W^−^^1^ @25 μW 22 mA W^−1^ @785 μW	0.2 V	>0.88 MHz	–	^163^
	2017	M-bPAs-M	2–8	PV	180–20.3 mA W^−1^ @ 0.07–44.3 μW (3.66 μm)	0 V	~0.65 kHz (4.03 μm) ~11.4 kHz (1.55 μm)	>1.06 × 10^8^ (2–8 μm)	^130^
	2017	M-BP-M	3.4	PC	518 mA W^−1^ @40 μW, 77 K	1.2 V	>>10 kHz (1.3 GHz estimated)	~2.67 × 10^10*^	^29^
			5		30 mA W^−1^ @50 μW, 77 K			~2.29 × 10^7*^	
			7.7		2.2 mA W^−1^ @100 μW, 77 K			~1.19 × 10^6*^	
	2017	M-BP-M M-bPAs-M	1–4.6	PC	~11 A W^−1^ @ RT (3.6 μm)	0.5 V	117 kHz (0.98 μm)	1 × 10^10^–6 × 10^10^ @1V	^129^
	2016	M-BP-M	3.39	PG	82 A W^−1^ @1.6 nW 0.9 A W^−1^ @30 μW	0.5 V	1.1–2.2 kHz	~1.2 × 10^8*^	^67^
	2018	M-BP-M	0.514–1.8	PG	5 × 10^3^–6 × 10^4^A W^−1^ @1.6 W cm^−2^, 70 K	2 V	~35 kHz (0.632 μm)	∼2.1 × 10^10^ (0.632 μm)	^143^
	2018	M-BP-M	1.55	PG	230 A W^−1^ @11 nW	1 V	~73 Hz	–	^144^
	2018	M-bAsP-M	3.4	PG PTE PV	190 mA W^−1^	1 V	–	~2.86 × 10^7*^	^164^
			5		16 mA W^−1^			~2.16 × 10^6*^	
			7.7		1.2 mA W^−1^			~1.86 × 10^5*^	
	2018	M-Te-M	1.4–2.4	PG	27 A W^−1^ @78 K (1.7 μm) 16 A W^−1^ @297 K (1.7 μm)	5 V	–	2.9 × 10^9^ @ RT 2.6 × 10^11^@ 78 K	^33^
	2019	M-Te-M	0.52	PG	383 A W^−1^ @1.6 nW	1 V	~1 kHz @ 0.95 nW	–	^131^
			1.55	PV	~19.2 mA W^−1^ @0~30 μW		37 MHz @39–250 μW		
			3.39	PG	~18.9 mA W^−1^ @0–30 μW		35 Hz @30 μW		
	2019	M-ReS_2_-M	0.8−1.2	BOL	380–350 A W^−1^	0.1 V	~117 Hz	~1.3 × 10^10^	^165^
	2020	M-PtSe_2_-M	0.765–1.55	PC PV	0.19 mA W^−1^ (1.55 μm)	5 V	4.5–17 GHz	~1.2 × 10^7^ (1.55 μm)*	^35^
Metal-2DM+X-metal	2014	M-G+Ta_2_O_5_+G-M	1.2	PG	20 A W^−1^	1 V	–	–	^166^
			2.4		0.45 A W^−1^				
	2015	M-G+CNT-M	0.405–1.55	PG	20 A W^−1^ @0.3 μW (1.55 μm)	0.5 V	~3.5 kHz (0.65 μm)	–	^76^
	2017	M-G+SiQDs-M	0.375–1.87	PG	1.2–22 × 10^8^ A W^−1^ @0.2 μW cm^−2^	1 V	sub-Hz scale	~10^13^ @RT	^75^
			2.5–3.9		0.22–44.9 A W^−1^ @375 mW cm^−2^			~10^5^ @77 K	
	2017	M-BP+G-M	1.55	PG	1300 A W^−1^ @ 11 nW 210 A W^−1^ @ 211 nW	1 V	~88 Hz	–	^46^
	2017	M-Au+G+Si-M	~1.55	PG	83 A W^−1^ @0.3 μW	10 V	~ 580 kHz	~10^8^	^78^
2DM- heterostructure	2016	G-WSe_2_-G	~1.55	IPE	0.12 mA W^−1^	0.6 V	–	–	^100^
	2016	WSe_2_-G-MoS_2_	0.4–2.4	PV	0.1–1 A W^−^^1^ (1.3–2.4 μm)	1 V	~ 7 kHz (0.53–0.94 μm)	2 × 10^9^–2 × 10^10^	^86^
	2018	G-GaSe-G	0.73	IPE	10 mA W^−1^	1 V	3.9 Hz	~5.76 × 10^7c^	^167^
			1.33		3 mA W^−1^		2.2 Hz	~1.73 × 10^7c^	
			1.55		0.05 mA W^−1^		1.5 Hz	~2.9 × 10^5c^	
	2019	G-hBN-G	~0.532	IPE F-N tunneling	13 μA W^−1^	Few volts	–	5 × 10^14^	^101^
			~1.55		70 nA W^−1^			–	
	2017	G-Si	~1.55	IPE	~20 mA W^−1^	10 V	–	5.1 × 10^7^	^136^
	2018	G-Si	2	IPE	0.16 mA W^−1^	0 V	–	2.56 × 10^7^	^138^
	2019	G nanowalls- Au-Si	1.55	IPE	21 mA W^−1^ @0.19 μW	1 V	~0.95 kHz	1.6 × 10^9^	^168^
			3.5		0.44 μA W^−^^1^	0 V	–	–	
	2020	CQDs+G-TiO_2_-Ti	1.625	PG	70 A W^−1^	0.5 V	1.1 kHz	~8.1 × 10^7* e^	^103^
	2020	WS_2_-HfS_2_	4.3–10	ILE	~92.4–3510 A W^−1^ @0.5 nW	1.5 V	100–200 Hz	7 × 10^12^ (7 μm)	^13^
	2016	BP-MoS_2_	~0.532	PV PG	22.3 A W^−1^ @1 nW	3 V	–	3.1 × 10^11^	^92^
			1.55		153.4 mA W^1^ @1 nW		~23.3 kHz	2.13 × 10^9^	
	2017	WS_2_-BP-MoS_2_	~0.532	PV PG	6.32 A W^−1^ @13.5 nW	3 V	–	1.01 × 10^9^	^93^
			1.55		1.12 A W^−1^ @13.5 nW			1.74 × 10^8^	
	2017	BP-MoS_2_	2–8	PV	115.4–216.1 mA W^−1^ (2.36–4.29 μm)	0 V	–	>4.9 × 10^9^ (3–5 μm)	^130^
	2018	BP-MoS_2_	1.6–4	PV	0.1–0.9 A W^−1^	0 V	~100 kHz	1.1 × 10^10^ (3.8 μm)	^94^
	2020	BP-MoS_2_	2–4	PV	0.11 A W^−1^ (3 μm)	0 V	~0.1–1 GHz	1.7 × 10^9^ (3.0 μm)	^95^

*GQDs* graphene quantum dot-like arrays, *CQDs* colloidal quantum dots, *SiQDs* Si quantum dots, *PV* photovoltaic, *IPE* internal photon emission, *DT* direct tunneling, *F-N tunneling* Fowler-Nordheim tunneling, *PC* photoconductive, *PG* photo-gating, *BOL* bolometric, *PTE* photo-thermoelectric, *ILE* interlayer exciton, *RT* room temperature.

^a^The measured values are counted.

^b^The data marked with asterisk (*) are extracted by using the provided NEP and the device active region area.

^c^Extracted by the measured data considering the shot noise and the thermal noise.

^d^The responsivity and the related specific detectivity D* may be overestimated because the optical absorption was ignored here.

^e^Extracted from the measured value *NEP* = 1.8 × 10^−11^ W at the modulation frequency of 30 Hz with a device active area of ~210 μm^2^.

As discussed in Section 2.2, the M-2DM+X-M PDs are mainly based on the PG effect due to the presence of trap states. These PDs usually have a high-specific detectivity at very low input optical power (e.g., 10^13^ Jones at ~0.2 μW cm^−2^ in ref. ^75^), while the bandwidth is usually in kHz scale, as shown in Table 2. In addition, their responsivities degrade seriously as the optical power increases due to the saturation of trap states^46,75^. In other words, it is necessary to further improve the linearity performance in order to satisfy the demands in many scenarios.

In contrast, 2DM-heterostructure PDs were developed until recently because of the fabrication process complexity. It is possible to achieve high sensitivity due to the dark current suppression. For example, there are several PDs reported with high-specific detectivity of over 10^10^ Jones^13,86,92,94,101^. However, they mostly have limited bandwidths e.g., less than MHz currently. Among these reported PDs, the BP-MoS_2_ heterostructure PD in ref. ^95^ has excellent performances overall, i.e., a bandwidth of ~0.1–1 GHz and a specific detectivity of 1.7 × 10^9^ Jones at 3.0 μm.

## Perspective and outlook

**Fig. 9:**
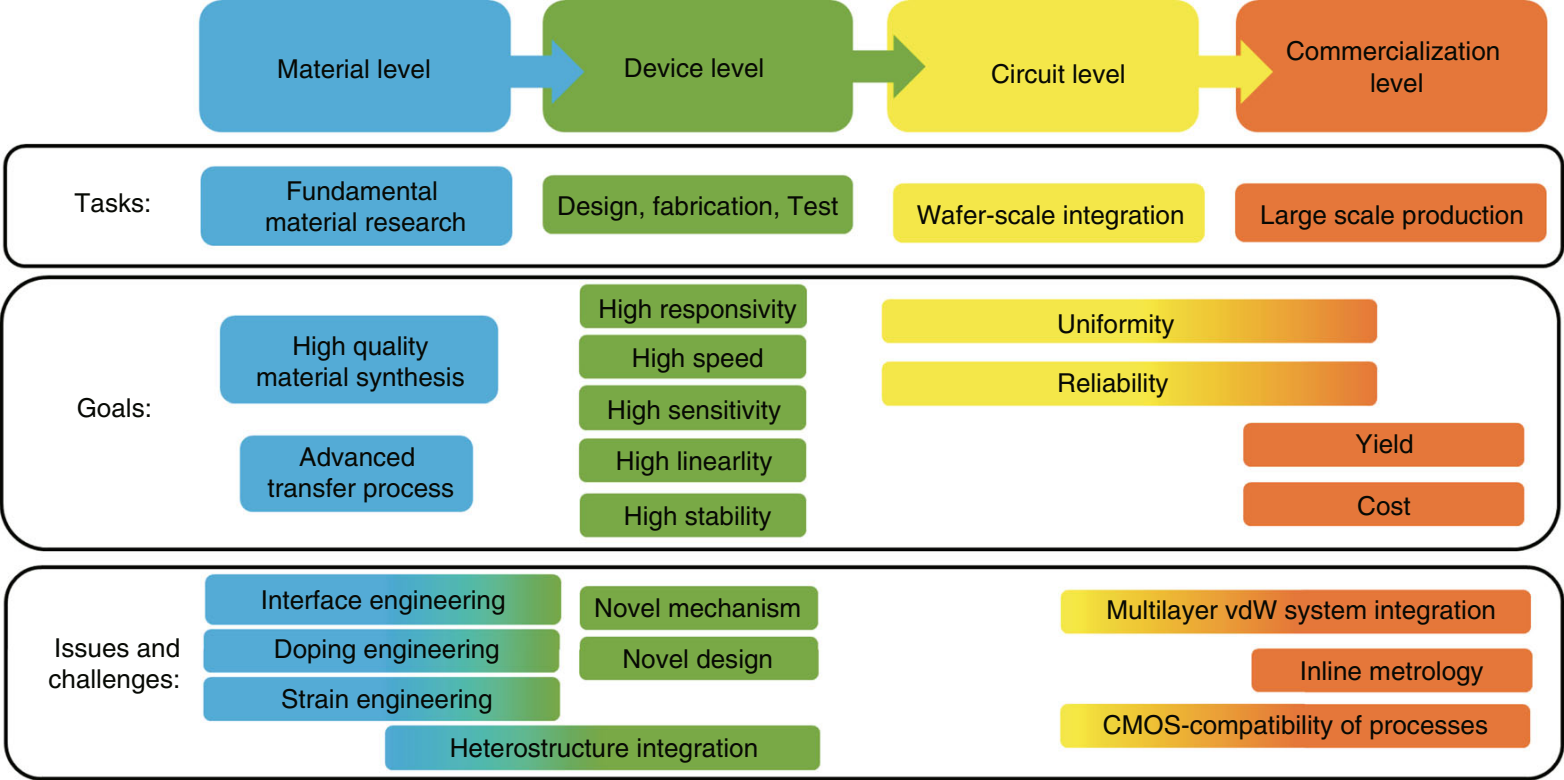
Development of Si/2DM PDs. The tasks, goals, and issues and challenges are summarized from the perspective of the material level, the device level, the circuit level, as well as the commercialization level.

Figure 9 gives the perspective of Si/2DM PDs according to four levels of material, device, circuit, and commercialization. The researches at the material level aim at the synthesis and the transfer process of 2DMs, providing the material foundation for the development of the devices. Great efforts have been devoted to explore and synthesize new 2DMs suitable for photodetection, i.e., with high mobilities as well as engineered bandgaps covering the NIR and MIR wavelength bands. As discussed above, various 2DMs have been demonstrated in the past years. Definitely, it is also important to develop high-quality transfer process for 2DMs. As is well known, the wet transfer process for CVD-grown 2DM films is one of the most popular options because it enables large-area transfer and thus is even useful for wafer-scale fabrication. For the wet transfer process, a polymer thin film is usually used as the assistant layer. Unfortunately, the polymer-removing process often induces the 2DM quality degradation^9^. To solve this problem, several direct transfer processes have been developed^145–147^. Another major transfer process is the mechanical exfoliation from vdW crystals, which can provide near-perfect high-quality 2DMs. In this way, however, only small-sized 2DM thin film with random shapes can be achieved and transferred if there is no special technique. Fortunately, it is shown that large-area mechanical exfoliation of graphene with controlled thickness can be achieved, as demonstrated recently in ref. ^148^, showing high potential for the device development in the future. In order to develop high-performance Si/2DM PDs, more efforts at the material level are desired for the interface engineering, including contact engineering^149^, doping engineering, and strain engineering^12^, as shown in Fig. 9.

As summarized in “Waveguide-integrated silicon-2DM PDs” and “Surface-illuminated Si/2DM PDs” sections, it is still very challenging to achieve Si/2DM PDs with high performances as well as high stability, which is the major goal for researches working for devices. More specifically, there is usually a trade-off between the response speed and the detection sensitivity. For some applications, such as optical imaging^150,151^ and spectrometry^152^, high sensitivity is extremely important, while moderate (or even low) response speed is acceptable (e.g., kHz-MHz). In this case, the PDs with the PG effect might be useful since their detection sensitivity could be very high for low optical power. Definitely one should note that the higher the PC gain is, the slower the response speed will be. Besides, it is still important to increase the saturated optical power for the PDs to achieve sufficient linear dynamic range, which might be enabled by introducing some novel device configurations and read-out structures. Similar problem is also faced by the BOL effect-based PDs^58,119^. As shown in Fig. 9, high linearity is an important requirement which should be paid more attention for the future development of 2DM PDs.

In the applications, such as next-generation optical interconnects^105^ and THz photonics^153^, it is usually desired to have PDs with ultra-large bandwidths of ~10^2^ GHz and beyond. In these scenarios, M-G-M PDs have shown unique advantages due to the ultrafast carrier dynamics in graphene. However, the SNR should be improved significantly. A potential solution is to use special device structures with extremely strong enhancement of light absorption, e.g., plasmonic structures^154^. In particular, waveguide-integrated 2DM-heterostructure PDs on silicon have been recognized as a promising candidate with potentially decent performances in responsivity, bandwidth, and sensitivity^122,123^. However, there remain several big challenges. First, the structural complexity of the 2DM-heterostructure PDs makes the fabrication more difficult compared to the M-2DM-M PDs. More efforts should be made to achieve reliable high-quality fabrication^155^. Furthermore, more fundamental work is desired for the carrier dynamics in various 2DM heterojunctions.

At the circuit level, it is expected to develop wafer-scale photonic integrated circuits based on 2DM PDs. In this case, it is required to realize high-performance PDs with high uniformity and high reliability. Currently, there have been several works demonstrated at the level of circuits, such as the optoelectronic integrated circuits with Si/2DM PDs discussed in “PDs and arrays for image sensing” section^124,125^. 2DM PDs have also been fabricated in wafer-scale, including the graphene PDs in ref. ^118^, the few-layer GaTe PDs in ref. ^156^, which are ready for large-scale integration on silicon. More recently, Giambra et al. presented the full process flow of wafer-scale integration of graphene-based photonic devices with improved uniformity^157^.

To the best of our knowledge, Si/2DM PDs have not been commercialized for applications yet. It is expected to happen in the following years with the great efforts from the groups working on materials, devices and circuits. Definitely, the yield and the cost are the keys. It is desired to develop the system-level multilayer vdW integration method^158^, the inline metrology technology^9^, as well as the quasi-CMOS-compatible process^9,105,159^. As predicted in ref. ^9^, Si/2DM PDs based on the back end of line CMOS-compatible technology might be brought to market in 2022, which will be very attractive and helpful for the development of silicon photonics as well as 2DM photonics.

## Conclusions

In this paper, we have given a review on recent progresses of Si/2DM PDs for the applications in the NIR/MIR wavelength bands. The operation mechanisms and the configurations for 2DM PDs have been summarized and discussed, including the waveguide-integrated and surface-illuminated devices. It can be seen that great progresses have been achieved, including ~100 GHz high-bandwidth M-G-M PDs, ultra-high-sensitivity PG effect-based PDs, and 2DM-heterostructure PDs with balanced bandwidths and sensitivities. Several representative wafer-scale image sensors based on Si/2DM PDs and the arrays have also been demonstrated. However, it is still very challenging to realize overall high-performance PDs in wafer-scale. Nevertheless, the combination of 2DMs and silicon microelectronics/photonics provides a promising technical route to realize high-performance and low-cost PDs, which might play an important role as the fundamental element in next-generation optoelectronic integrated chips.

## Supplementary information

Supplementary Information

Copyright permission files
