# Supplementary material for: Silicon/2D-material photodetectors: from near-infrared to mid-infrared
Source: Light Sci Appl. 2021 Jun 9;10:123. doi: 10.1038/s41377-021-00551-4 (PMC8190178; doi:10.1038/s41377-021-00551-4)
Supplement: Supplementary file 1 — Supplementary Information [file 41377_2021_551_MOESM1_ESM.docx]

**Supplementary Information for**

**Silicon/2D-material photodetectors: from near-infrared to mid-infrared**

Chaoyue Liu^1,3^, Jingshu Guo^1,3^, Laiwen Yu^1^, Jiang Li^1^, Ming Zhang^1,2^, Huan Li^1^, Yaocheng Shi^1,2^, and Daoxin Dai^1,2^ *

*1. State Key Laboratory for Modern Optical Instrumentation, Zhejiang Provincial Key Laboratory for Sensing Technologies, College of Optical Science and Engineering,* *International Research Center for Advanced Photonics, Zhejiang University, Zijingang Campus, Hangzhou, 310058, China.*

*2. Ningbo Research Institute, Zhejiang University, Ningbo 315100, China.*

*3. These authors contribute equally.*

*Corresponding author e-mail: dxdai@zju.edu.cn.

**Supplementary Note 1. Figures of merits of two-dimensional material photodetectors**

In order to evaluate the device performances^1,2^, in this part we introduce the Figures of merits (FOMs) related to the responsivity, the response speed, the sensitivity, and the linearity of the photodetectors (PDs). More details about the definitions of the FOMs of PDs as well as two-dimensional material (2DM) PDs can be found in refs. ^3,4,-5^ and refs. ^6,7,-8^, respectively.

**1.1 Responsivity-related FOMs**

The responsivity *R* is defined as the ratio between the photoresponse signal (i.e., the photocurrent *I*_ph_ in Ampere or the photovoltage *V*_ph_ in Volt) and the incident light power *P*_in_ in Watt, i.e., *R* = *I*_ph_/*P*_in_ in A W^-1^ or *V*_ph_/*P*_in_ in V W^-1^. The former expression (*I*_ph_/*P*_in_) is widely used in the PDs based on the PV effect, the tunneling effects, and the IPE effect. For these effects, the photoresponse comes from the direct detection of the photo-excited carriers. The latter definition (*V*_ph_/*P*_in_) suits for various photoconductors^3^ based on the PC-type mechanisms including the PC-, PG-, and BOL-effects. For those PDs with the PTE effect, the responsivity *V*_ph_/*P*_in_ in V W^-1^ is often used since the photoresponse can be denoted by the photovoltage.

There are several concepts related to the responsivity. One is the external quantum efficiency (EQE), which describes the ratio between the number of photo-excited *e*-*h* pairs contributing to photoresponse and the incident photon number in unit time. Accordingly, one has *EQE*=*Rhc*/(*eλ*), where *h* is Planck constant, *c* is the speed ​​of light in vacuum, *λ* is the light wavelength, and *e* is unit charge. The internal quantum efficiency (*IQE*), which describes the photocarrier transmission efficiency of a PD, is related with the *EQE* by *EQE*=*IQE*·*η*_abs_, where *η*_abs_ is the light absorption efficiency in the PD.

As is well known, waveguide-integrated PDs can flexibly extend the length of the light absorption region, which usually enables sufficiently high *η*_abs_. In contrast, for normal-incident PDs, the light absorption is usually weak because the absorption layer has atomic layer thickness. As a result, the light-2DM interaction enhancement becomes very important and there have been reported with some specific structures such as F-P cavities^9,10^, photonic-crystal microcavities^11^, as well as plasmonic structures^12^. For the PDs with gain (*Gain*) such as avalanche gain or photoconductive gain, the *EQE* is then given by *EQE*·*Gain*=*Rhc*/(*eλ*)^13^.

Currently, many 2DM PDs based on the PG effect have been reported with high responsivities of 10-10^10^ A W^-1^ due to the high photoconductive gain^6,13,14^, while the other types of PDs usually have responsivities less than 1 A W^-16,13,14^. Even though high responsivity is usually helpful for realizing sensitive PDs, it is the sensitivity rather than the responsivity used for determining the minimal detectable optical power. In practice, the responsivity usually varies as a function of the bias voltage. More specifically, for photo-conductive PDs, the responsivity is usually proportional to the bias voltage. However, the dark current also increases greatly with the bias voltage, and thus the shot noise increases notably. For PDs with a large gain, there is some high generation-recombination noise.

In short, the sensitivity is more preferred than the responsivity in order to evaluate the low-power detection ability of PDs. Certainly, to achieve a high responsivity is also important for signal identification of the electric amplifier and readout circuits.

**1.2 Response-speed-related FOMs**

To evaluate the response speed of PDs, the 3-dB bandwidth *f*_3dB_ is one of the most widely used FOMs. The 3-dB bandwidth *f*_3dB_ is usually measured through the frequency-domain microwave vector network analyzer. Definitely the PD responses can also be tested in the time domain. There is an empirical formula to estimate the device bandwidth *f*_3dB_ from the measured rise time *t*_rise_. Here the rise time *t*_rise_ is defined as the time for the output signal rising from 10% to 90% of the maximum value, and the fall time *t*_fall_ is defined similarly. For example, one has *f*_3dB_ = 0.35/*t*_rise_ for square wave signals^15^. In contrast, for Gaussian optical pulses, the 3-dB bandwidth *f*_3dB_ is estimated as *f*_3dB_ =0.441*/t*_FWHM_ when the full width at half maximum *t*_FWHM_ for the output pulse is much larger than that of the input signal^16^.

In principle, the response speed of PDs is mainly limited by the following two factors. The *first* one is the speed of photoresponse generation. For those PDs with the PV-, tunneling-, or IPE- effect, the time for the photo-excited carriers to arrive at the electrodes may limit the PD bandwidth. For PDs with the PTE- or BOL-effects, the bandwidth might be limited by the time of the thermal relaxation process, which fortunately is usually very quick in 2DMs. The *second* one is the RC-bandwidth *f*_RC-3dB_ of the equivalent microwave electric circuit for a PD. For a simplified RC circuit with a resistance of *R*_0_ and a capacitance of *C*_0_, the RC-limited bandwidth is given by *f*_RC-3dB_=1*/*2π*τ*_RC_, where the RC time constant is given by *τ*_RC_=*R*_0_*C*_0_^3^.

In theory, most working mechanisms in 2DM PDs can provide fast photoresponses, while the 3-dB-bandwidth *f*_3dB_ is usually limited by the RC-bandwidth *f*_RC-3dB_. In recent years, high-speed PDs have been realized successfully with large bandwidths in GHz scale by utilizing various mechanisms, e.g., the PC effect^17,18^ and the PV effect in metal-2DM-metal configurations^19,20,-21^ and 2DM-heterostructures^22,23^, the BOL effect^17,24,25^, the PTE effect^26,27,28,29,30,31,-32^, the IPE effect^33^, and the tunneling effect^34^. In contrast, for PDs with the PG effect, the photoresponse is strongly dependent on the carrier trapping time related to the gain. The PDs with higher gain usually have slower responses. For example, the reported PDs with the PG effect usually have a 3-dB-bandwidth *f*_3dB_ in the scale of 0.1 Hz-100 kHz^6,13,14^.

**1.3 Sensitivity-related FOMs**

In practice, the signal-to-noise ratio (SNR) decides how sensitive a PD can be. There are several sensitivity-related FOMs. The *first* one is the dark current *I*_dark_, which is the current of a PD without illumination. The *second* is the normalized photocurrent to the dark current ratio (*NPDR*), which is defined as the optical power at which the photocurrent equals the dark current, i.e., *NPDR*=*I*_dark_/*R*, where *R* is the responsivity. For a deep evaluation for the PD sensitivity, the noise analysis should be involved. The noise in a PD is mainly from the thermal noise (Johnson–Nyquist noise) $\left\langle i_{nJ}^{2} \right\rangle^{1/2}$, the shot noise $\left\langle i_{ns}^{2} \right\rangle^{1/2}$, the 1/*f* noise $\left\langle i_{1/f}^{2} \right\rangle^{1/2}$, and the generation-recombination (g-r) noise $\left\langle i_{g-r}^{2} \right\rangle^{1/2}$. They are given as follows:

$\left\langle i_{nJ}^{2} \right\rangle=\frac{4k_{B}T\Delta f}{R_{0}}$ (Eq. S1a)

$\left\langle i_{ns}^{2} \right\rangle=2e(I_{d}+I_{ph})\Delta f$ (Eq. S1b)

$\left\langle i_{1/f}^{2} \right\rangle=k_{1}\frac{I^{b}\Delta f}{f^{a}}$ (Eq. S1c)

$\left\langle i_{g-r}^{2} \right\rangle=4eI_{ph}G_{ain}\Delta f$ (Eq. S1d)

where *k*_B_ is the Boltzmann constant, *T* is the temperature, △*f* is the measured bandwidth, and *R*_0_ is the resistance, *k*_1_, *a*, and *b* are several coefficients related to the fabrication process, the material properties, and the structures of the specific PD^3^. Note that the 1/*f* noise is usually non-negligible in the frequency range lower than 1 kHz, and the g-r noise is a special shot noise in the photoconductive-type PDs with the photoconductive gain^3^.

The total noise power is given by the sum of all noise terms. For a PD without gain, one has $\left\langle i_{n}^{2} \right\rangle$=$\left\langle i_{nJ}^{2} \right\rangle$+$\left\langle i_{ns}^{2} \right\rangle+\left\langle i_{1/f}^{2} \right\rangle$. The frequency-normalized noise equivalent power (*NEP*) is then given by $NEP=\frac{\left\langle i_{n}^{2} \right\rangle^{1/2}}{R{\Delta f}^{1/2}}$ (W Hz^-1/2^), which means the input optical power generating a unit SNR at 1Hz bandwidth and reflects the minimum detectable optical power of a PD.

In particular, for normal-incident PDs, the specific detectivity *D** is also used widely and one has $D^{*}=\frac{\sqrt{A}}{NEP}$ with unit of Jones (1 Jones= 1 cm Hz^1/2^ W^-1^), where *A* is the detector illumination area. The measured specific detectivity is usually preferred for the performance evaluation of a PD. For the theoretical estimation of the specific detectivity *D**, all the noise terms should be considered, including the 1/*f* noise term which is usually ignored^2^. Otherwise, the sensitivity might be overestimated. In addition, the specific detectivity *D** is a function of the wavelength, the input optical power, the modulation frequency, and the bandwidth^2^. It is important to state the specific conditions for the measured or calculated detectivity *D**. As demonstrated, the specific detectivity of the PDs with the PG effect might be very high (e.g., up to 10^14^ Jones^6,13,14^) at low input optical power due to the photoconductive gain. In contrast, the metal-2DM-metal PDs without gain usually do not have high sensitivity due to two reasons. First, the thermal noise is usually large because there is no junction in the channel and consequently the resistance is low (e.g. ~10^2^ Ω in metal-graphene-metal structures^24,25^). Second, the shot noise is high due to the large dark current when the bias voltage is applied. To solve these problems, a potential solution is using non-zero-bandgap 2DMs or applying a low (even zero) bias voltages^17^. Nevertheless, the SNR for these 2DM PDs still needs to be improved greatly.

**1.4 FOM for Linearity**

The linearity for the dependence of the photocurrent *I*_ph_ on the optical power *P*_in_ is an important metric for PDs^2^. The FOM of linear dynamic range (*LDR*) defined as *LDR*= 10lg(*P*_inmax_/*P*_inmin_) is often used to evaluate the linearity if the responsivity is constant within the power range from *P*_inmin_ to *P*_inmax_. Generally speaking, the PDs directly detecting photo-excited carriers are usually able to provide a good linearity. In contrast, for PDs with the PG effect, the trap states may get saturated at a moderate power *P*_in_ and the responsivity drops significantly when the power *P*_in_ increases further, which limits the operation range of the input power to the PDs.

References：

Konstantatos,G. Current status and technological prospect of photodetectors based on two-dimensional materials. *Nat. Commun.* **9**, 5266 (2018).

Fang, Y. J. *et al*. Accurate characterization of next-generation thin-film photodetectors. *Nat. Photon*. **13**, 1-4 (2019).

George, R. Detection of Light: from the Ultraviolet to the Submillimeter. *Cambridge University Press*, 2003.

Sze, S. M. *et al*. Physics of Semiconductor Devices (John Wiley and Sons, 1981).

Scales, C. and Berini P. Thin-film Schottky barrier photodetector models. *IEEE J. Quantum Electron.* **46**, 633-643 (2010).

Long, M. S. *et al*. Progress, challenges, and opportunities for 2D material based photodetectors. *Adv. Funct. Mater*. **29**, 1803807 (2019).

Xiong, Z. *et al*. Two-dimensional materials and hybrid systems for photodetection. *Synthesis, Modelling and Characterization of 2D Materials and their Heterostructures*. Elsevier, 325-349 (2020).

Buscema, M. *et al*. Photocurrent generation with two-dimensional van der Waals semiconductors. *Chem. Soc. Rev.* **44**, 3691-3718 (2015).

Amani, M. *et al*. Solution-synthesized high-mobility tellurium nanoflakes for short-wave infrared photodetectors. *ACS Nano* **12**, 7253-7263 (2018).

Yan, W. *et al*. Spectrally Selective Mid-Wave Infrared Detection Using Fabry-Pérot Cavity Enhanced Black Phosphorus 2D Photodiodes. *ACS Nano* **14**, 13645-13651 (2020).

Efetov, D. K. *et al*. Fast thermal relaxation in cavity-coupled graphene bolometers with a Johnson noise read-out. *Nat. Nanotechnol*. **13**, 797-801 (2018).

Yan, S. Q. *et al*. 2D materials integrated with metallic nanostructures: fundamentals and optoelectronic applications. *Nanophotonics, doi:10.1515/nanoph-2020-0074* (2020).

Koppens, F. H. L. *et al*. Photodetectors based on graphene, other two-dimensional materials and hybrid systems. *Nat. Nanotechnol.* **9**, 780-793 (2014).

Chen, X. Q. *et al*. Graphene hybrid structures for integrated and flexible optoelectronics. *Adv. Mater.* https://doi.org/10.1002/adma.201902039 (2019).

Bogatin, Eric. *Signal integrity: simplified* (Prentice Hall Professional, 2004).

Pospischil, A. *et al*. CMOS-compatible graphene photodetector covering all optical communication bands. *Nat. Photon.* **7**, 892-896 (2013).

Guo, J. S. *et al*. High-performance silicon-graphene hybrid plasmonic waveguide photodetectors beyond 1.55 μm. *Light-Sci. Appl.* **9**, 29 (2020).

Maiti, R. *et al.* Strain-engineered high-responsivity MoTe_2_ photodetector for silicon photonic integrated circuits. *Nat. Photon*. **14**, 578–584 (2020).

Youngblood, N. *et al*. Waveguide-integrated black phosphorus photodetector with high responsivity and low dark current. *Nat. Photon.* **9**, 247-252 (2015).

Yin, Y. L. *et al*. High-speed and high-responsivity hybrid silicon/black-phosphorus waveguide photodetectors at 2 μm, *Laser Photon. Rev.* **13**, 1900032 (2019).

Ding, Y. H. *et al*. Ultra-compact integrated graphene plasmonic photodetector with bandwidth above 110 GHz. *Nanophotonics* **9**, 317-325 (2020).

Ma, P. *et al*. Fast MoTe_2_ waveguide photodetector with high sensitivity at telecommunication wavelengths. *ACS Photonic* **5**, 1846–1852 (2018).

Flöry, N. *et al.* Waveguide-integrated van der Waals heterostructure photodetector at telecom wavelengths with high speed and high responsivity. *Nat. Nanotechnol.* **15,**118–124 (2020).

Schall, D. *et al*. Graphene photodetectors with a bandwidth> 76 GHz fabricated in a 6'' wafer process line. *J. Phys. D*: *Appl. Phys.* **50**, 124004 (2017).

Ma, P. *et al*. Plasmonically enhanced graphene photodetector featuring 100 Gbit/s data reception, high responsivity, and compact size. *ACS Photonics* **6**, 154-161 (2019).

Shiue, R. J. *et al*. High-responsivity graphene–boron nitride photodetector and autocorrelator in a silicon photonic integrated circuit. *Nano Lett.* **15**, 7288-7293 (2015).

Schuler, S. *et al*. Controlled generation of a p–n junction in a waveguide integrated graphene photodetector. *Nano Lett.* **16**, 7107-7112 (2016).

Schuler, S. *et al*. Graphene Photodetector integrated on a photonic crystal defect waveguide. *ACS Photonics* **5**, 4758-4763 (2018).

Muench, J. E. *et al*. Waveguide-integrated, plasmonic enhanced graphene photodetectors. *Nano Lett.* **19**, 7632-7644 (2019).

Schuler, S. et al. High-responsivity graphene photodetectors integrated on silicon microring resonators. Preprint at https://arxiv.org/abs/2007.03044 (2020).

Marconi, S. *et al*. Photo Thermal Effect Graphene Detector Featuring 105 Gbit s-1 NRZ and 120 Gbit s-1 PAM4 Direct Detection. Preprint at https://arxiv.org/abs/2006.01481 (2020).

Mieikis, V. *et al*. Ultrafast, zero-bias, graphene photodetectors with polymeric gate dielectric on passive photonic waveguides. *ACS Nano* **14**, 11190–11204 (2020).

Li, T. T. *et al*. Spatially controlled electrostatic doping in graphene *p*-*i*-*n* junction for hybrid silicon photodiode. *npj 2D Mater. Appl.* **2**, 36 (2018).

Gao, Y. *et al*. High-speed van der Waals heterostructure tunneling photodiodes integrated on silicon nitride waveguides. *Optica* **6**, 514-517 (2019).
