# Supplementary material for: Silicon/2D-material photodetectors: from near-infrared to mid-infrared
Source: Light Sci Appl. 2021 Jun 9;10:123. doi: 10.1038/s41377-021-00551-4 (PMC8190178; doi:10.1038/s41377-021-00551-4)

Here we provide the copyright permission files for the re-use figures of non-open access journals.

For Fig. 3b and Fig. 4b:


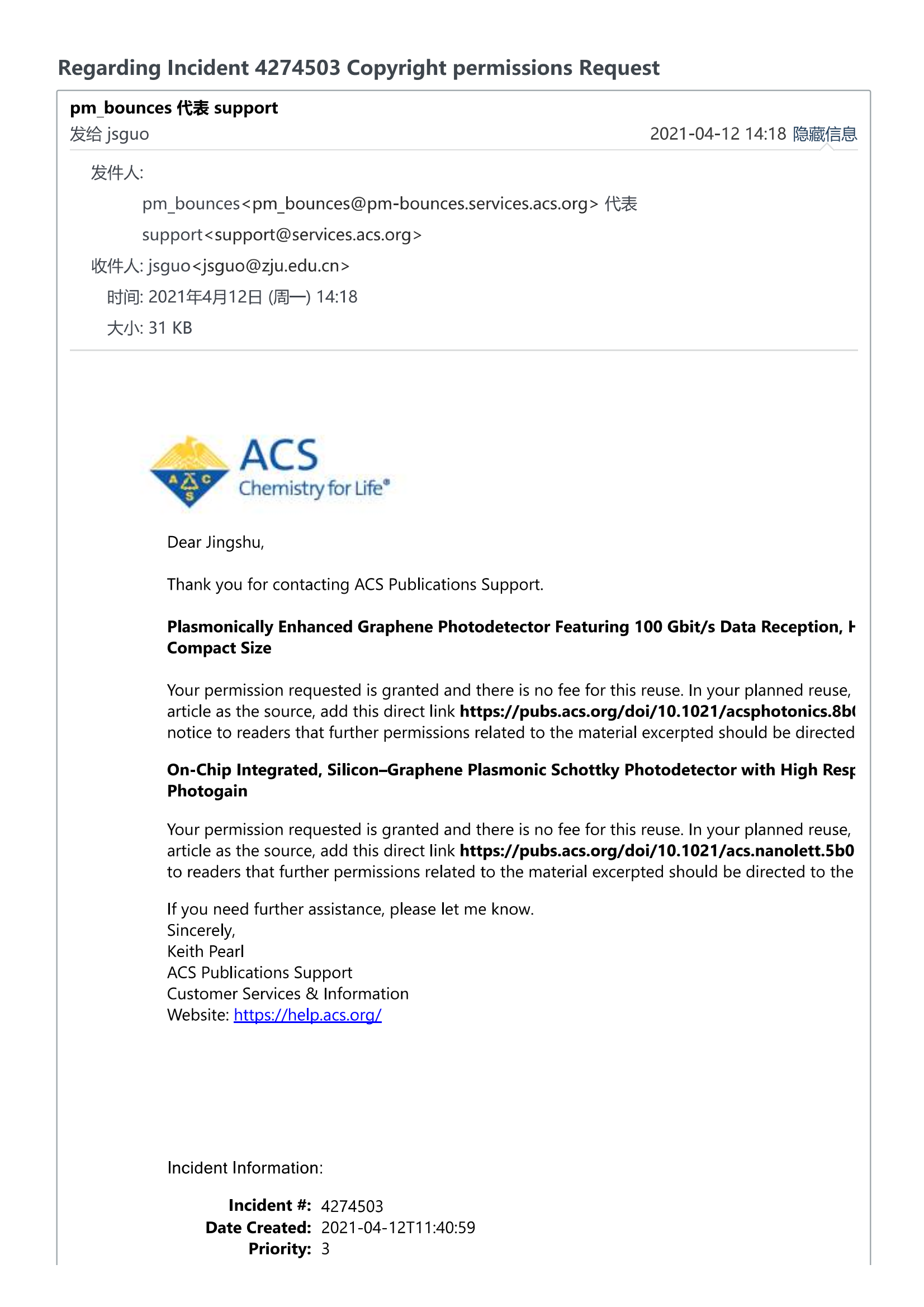

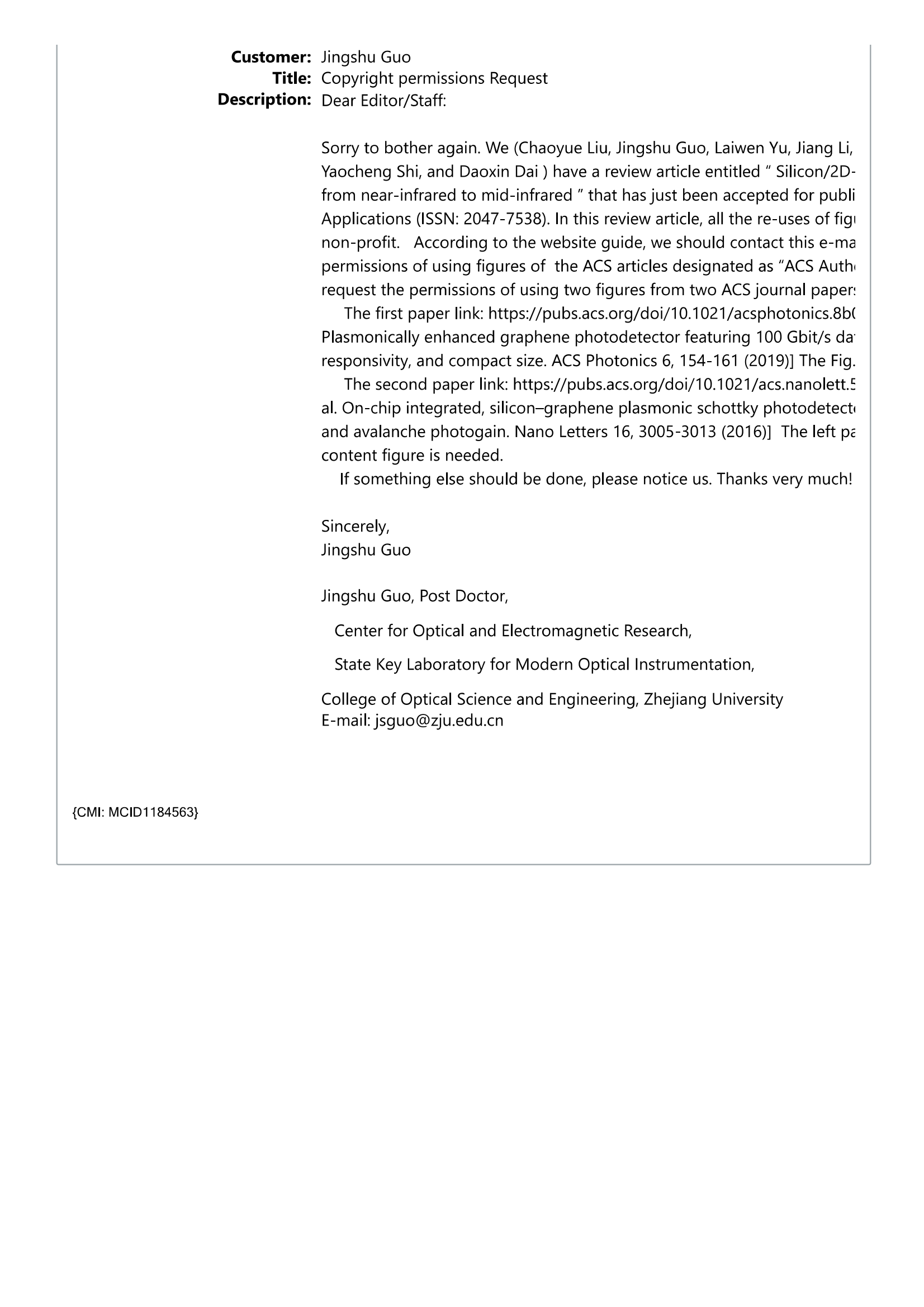


For Fig. 3c:


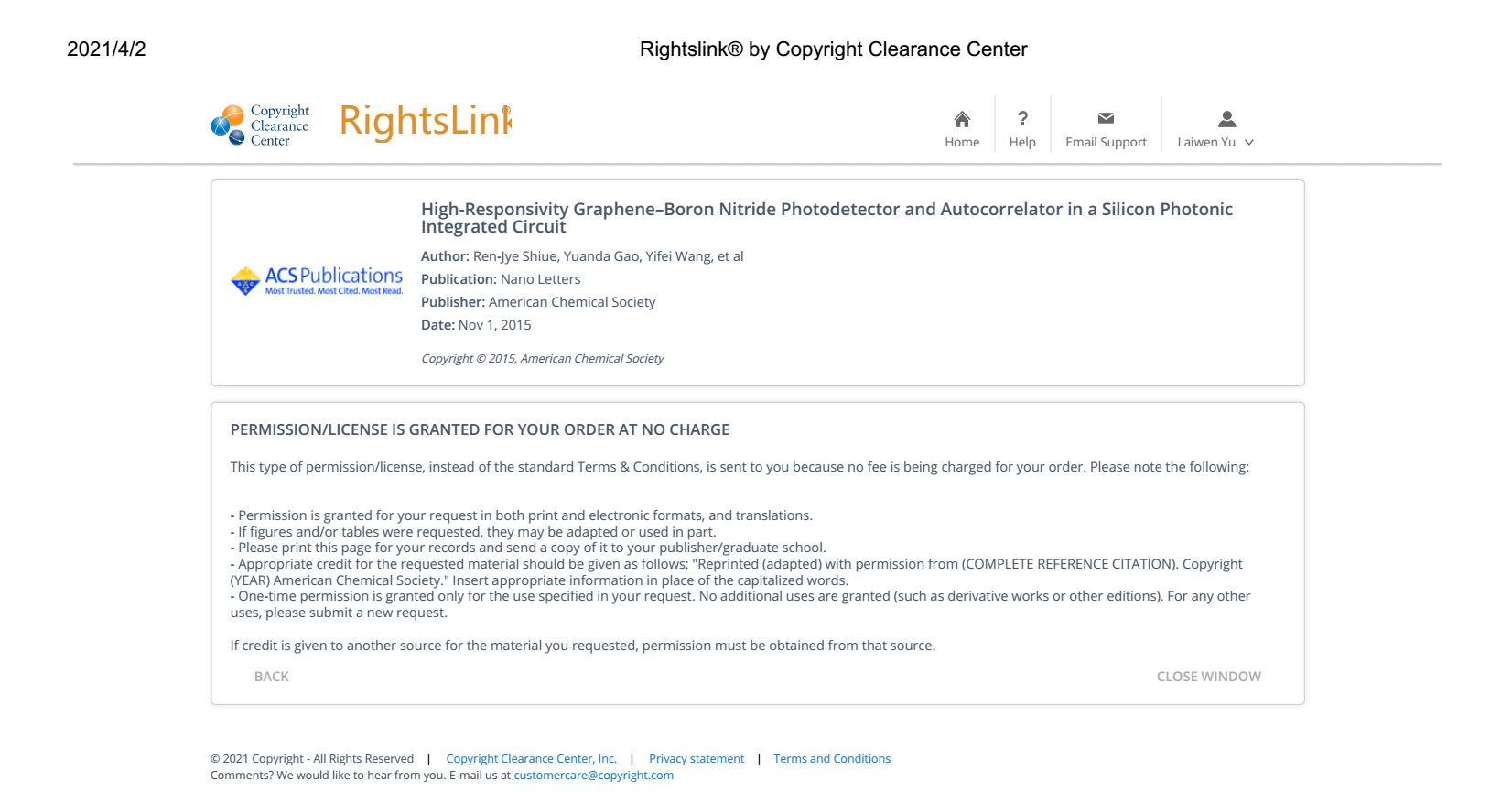


For Fig. 3f:


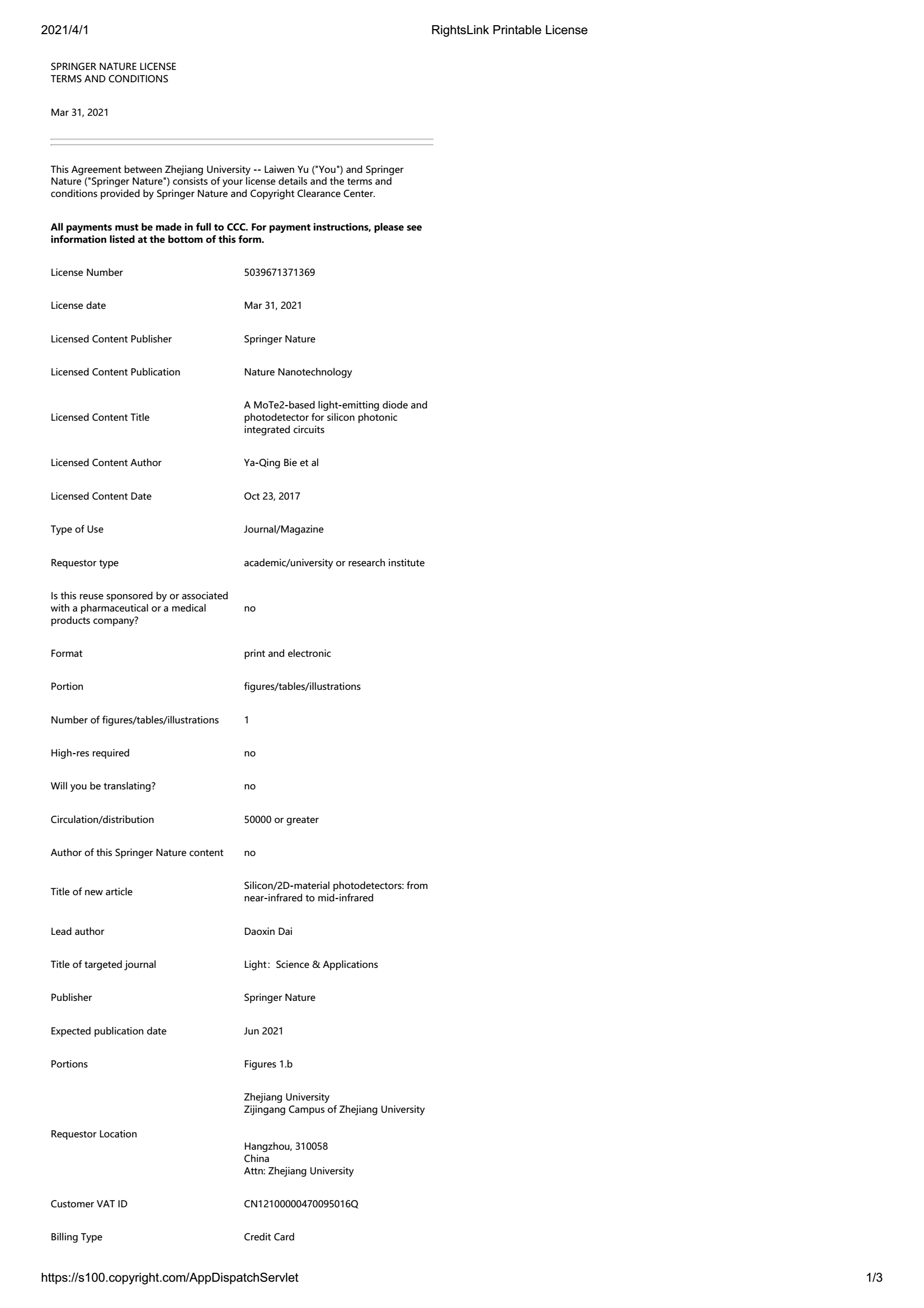

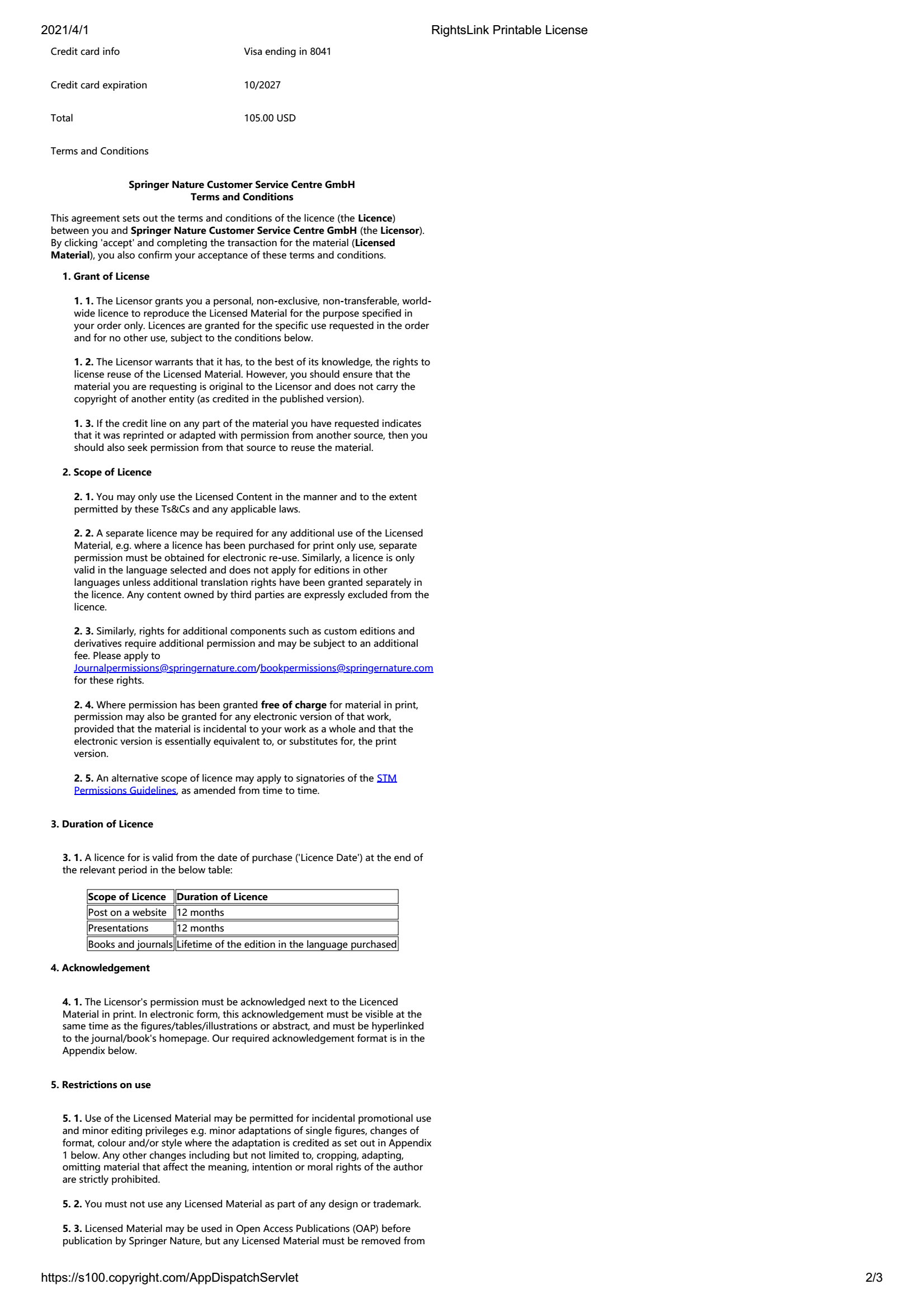


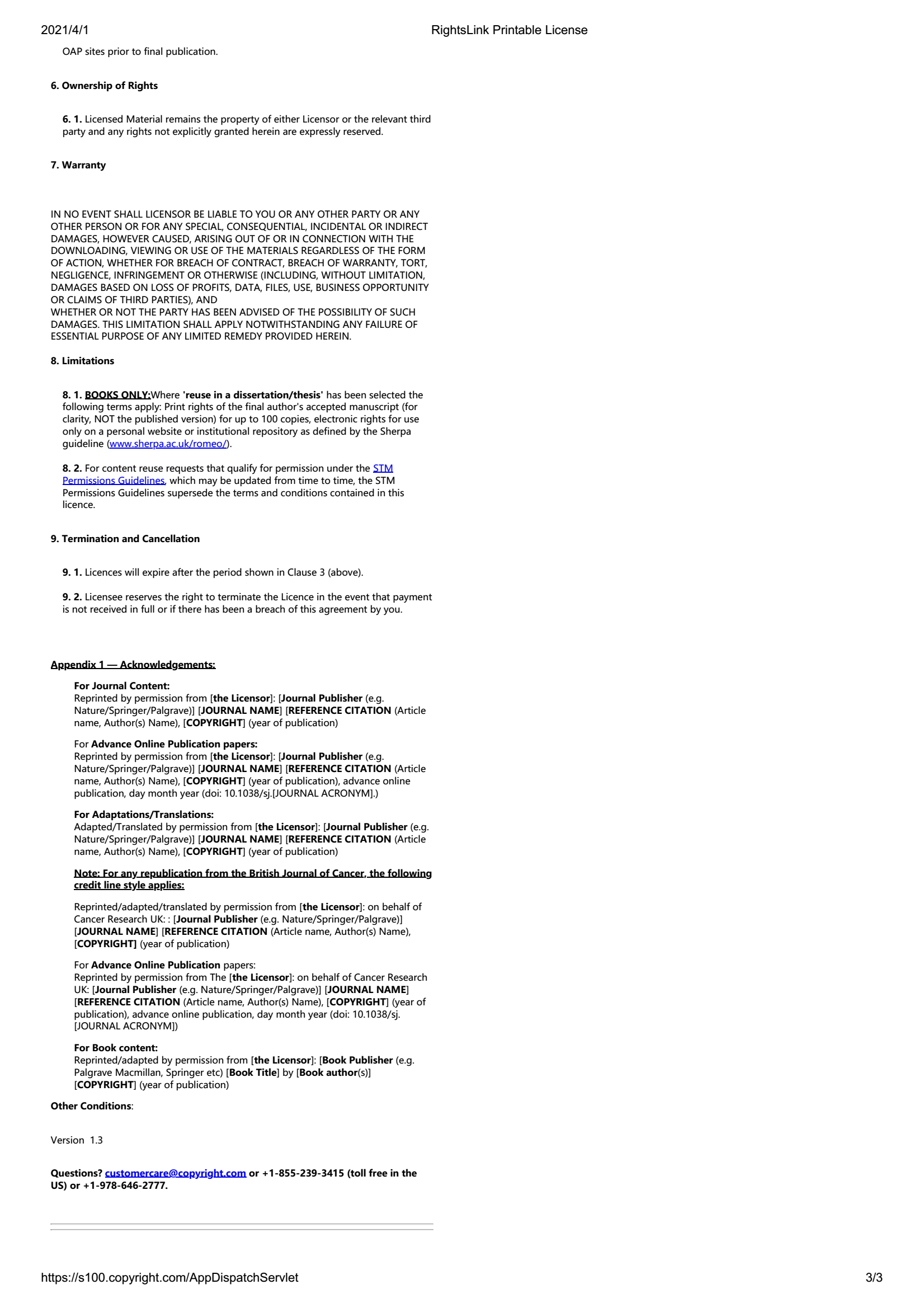


For Fig. 3g:


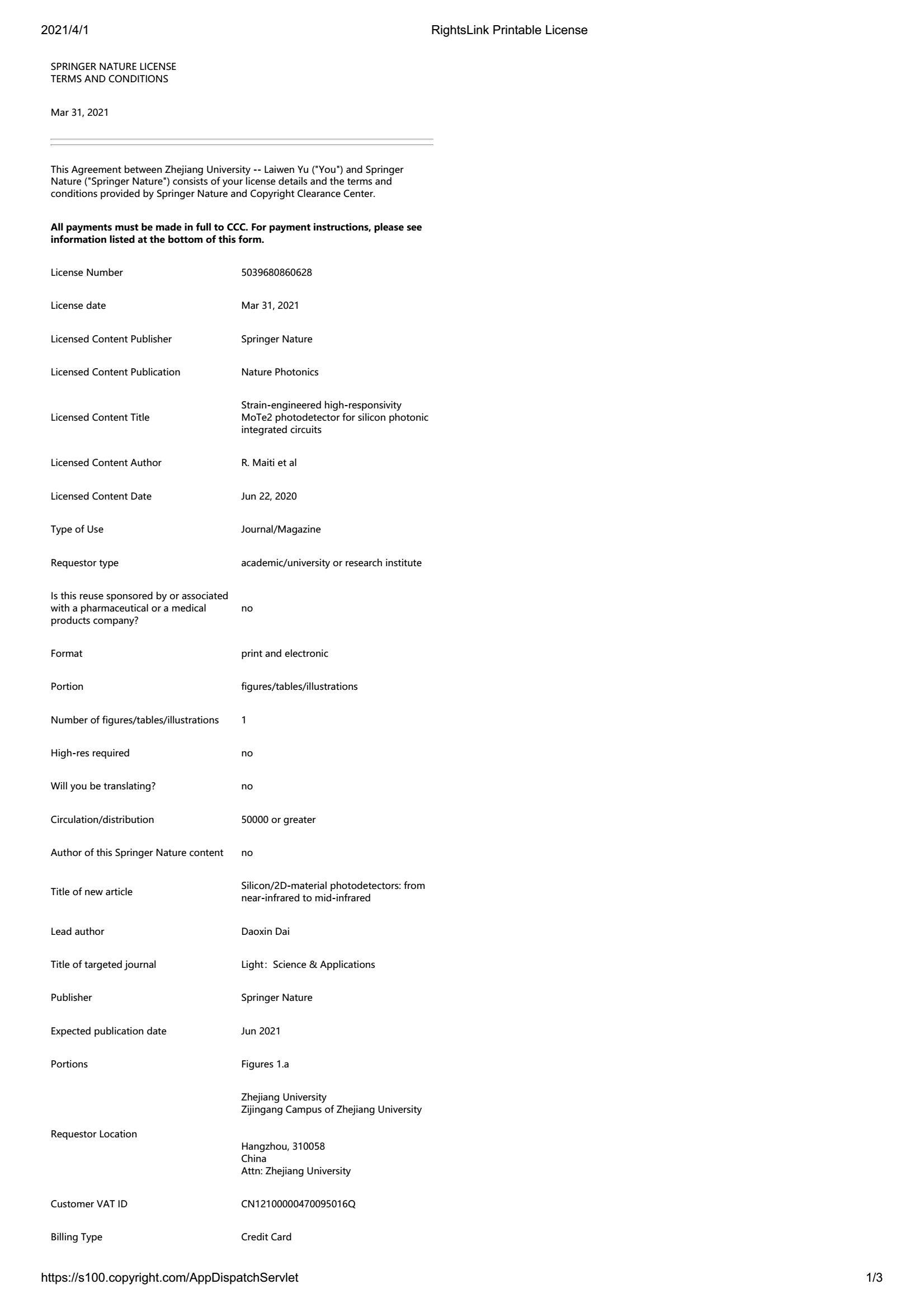

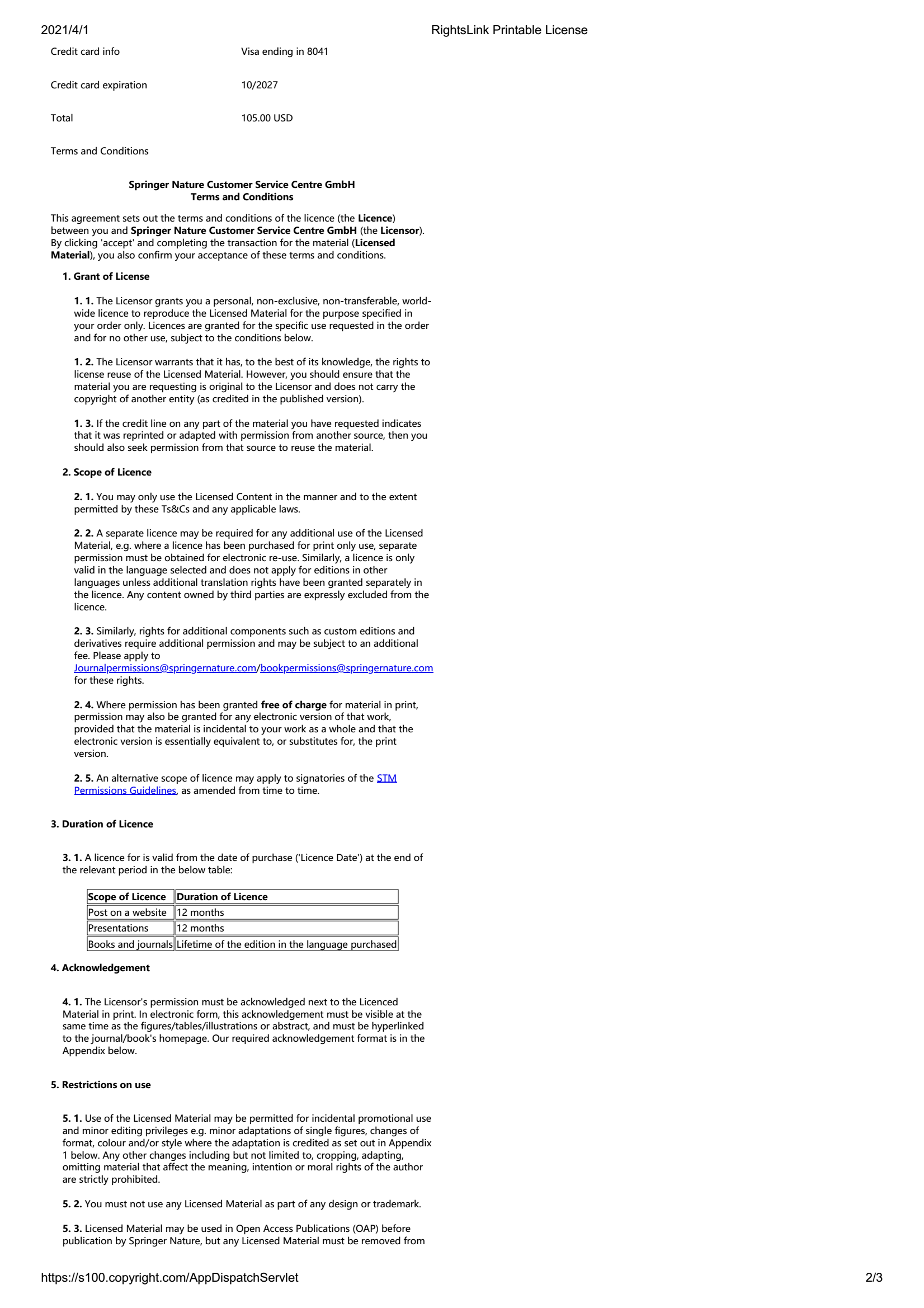


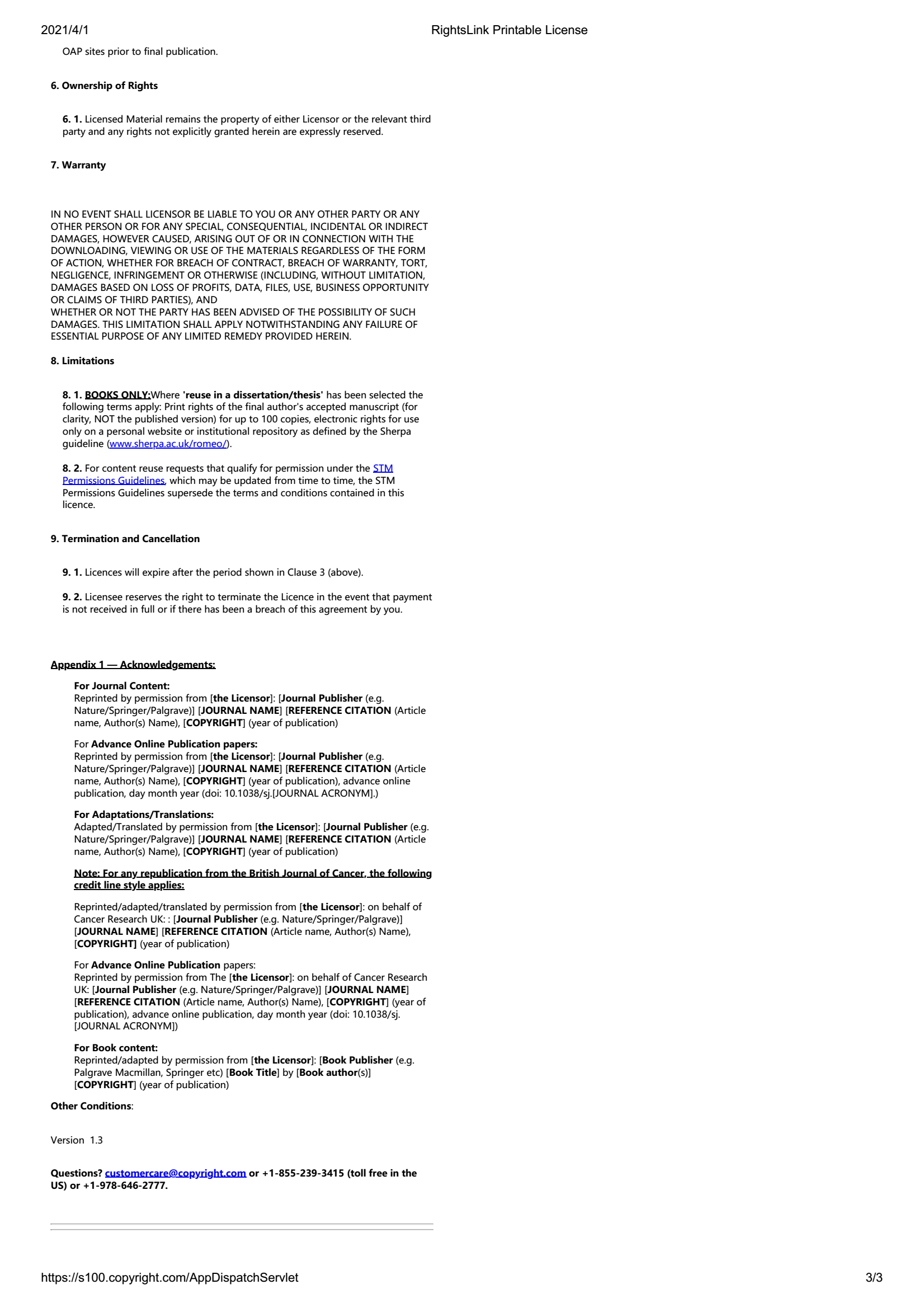


For Fig. 3h


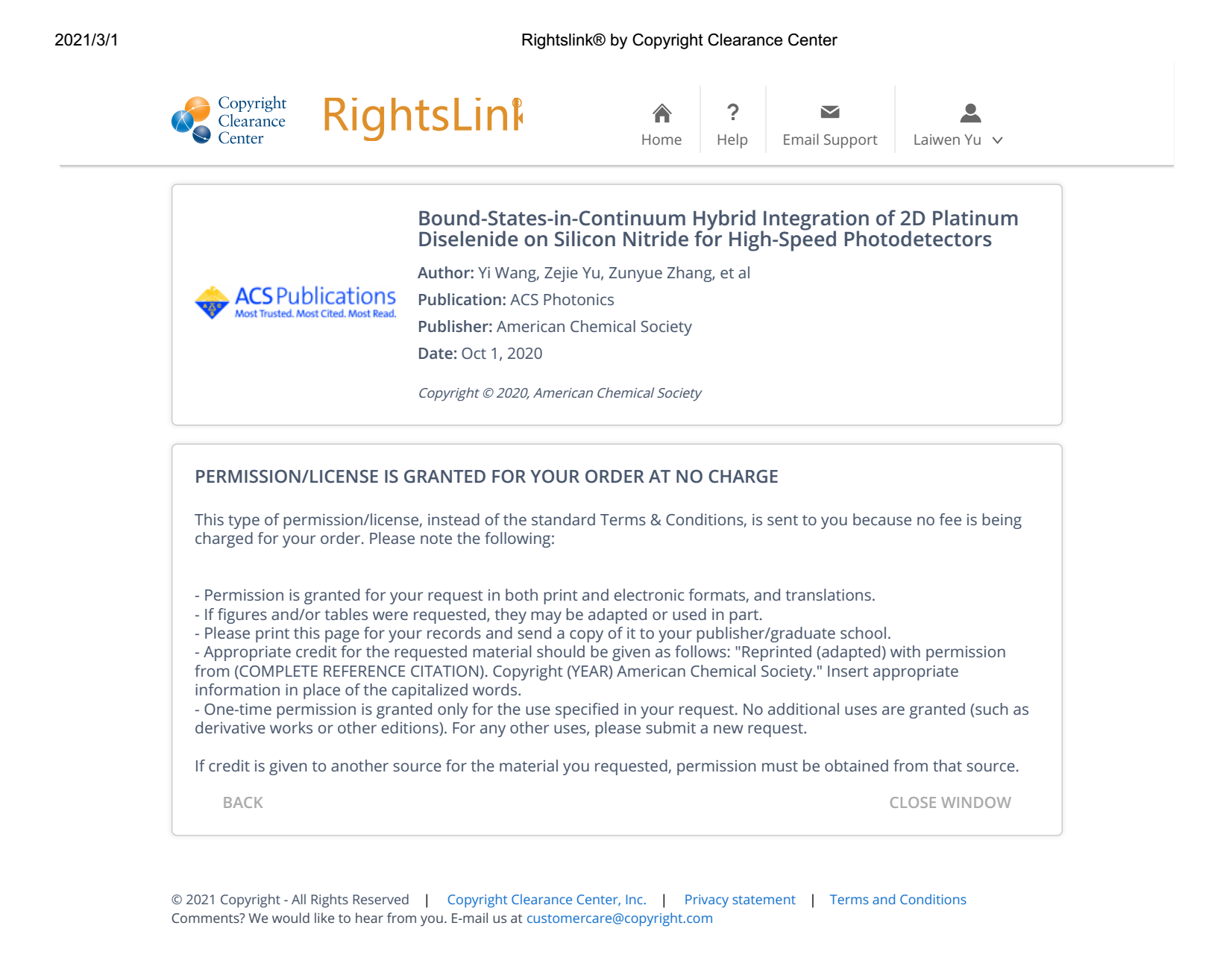


For Fig. 3i


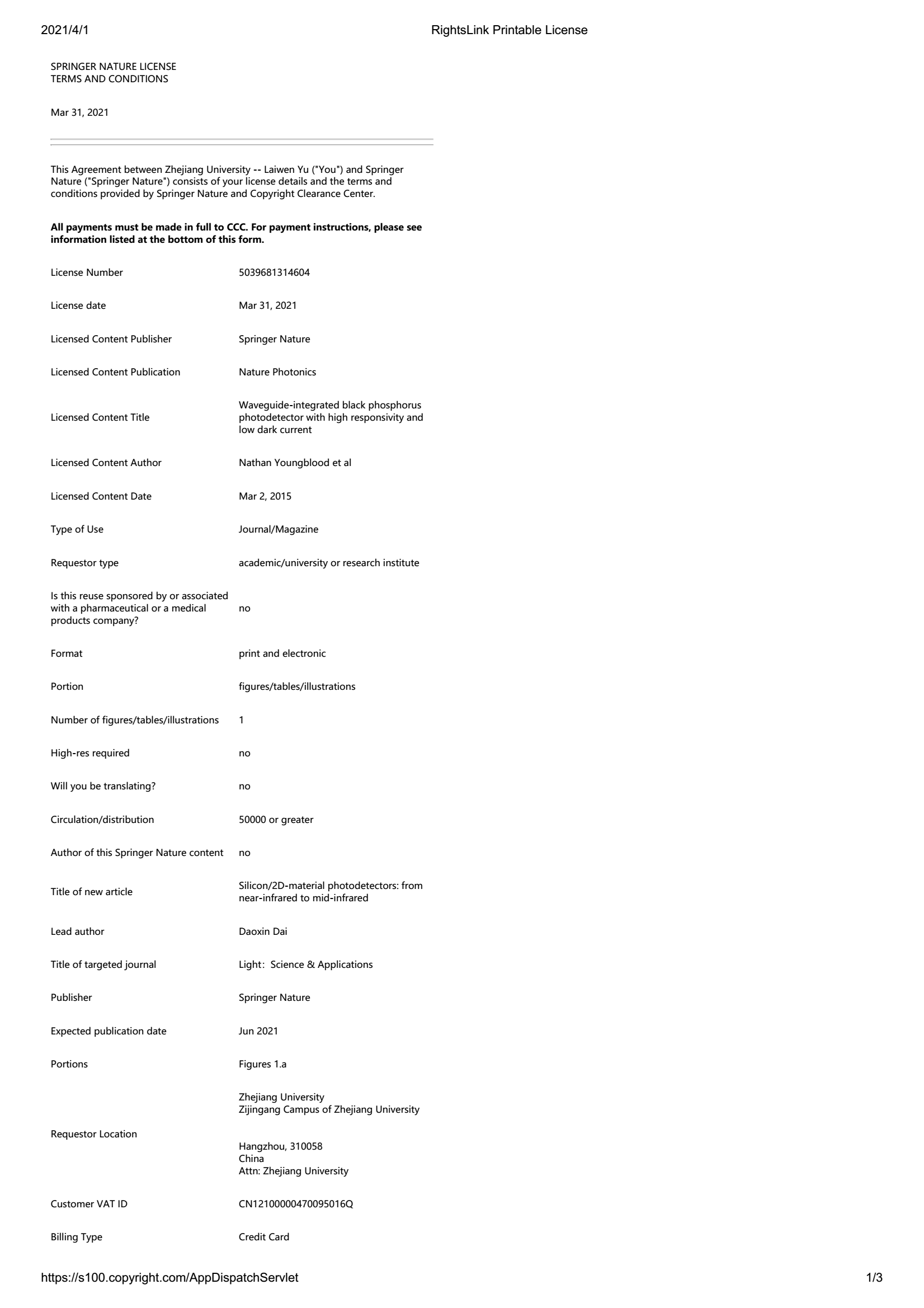

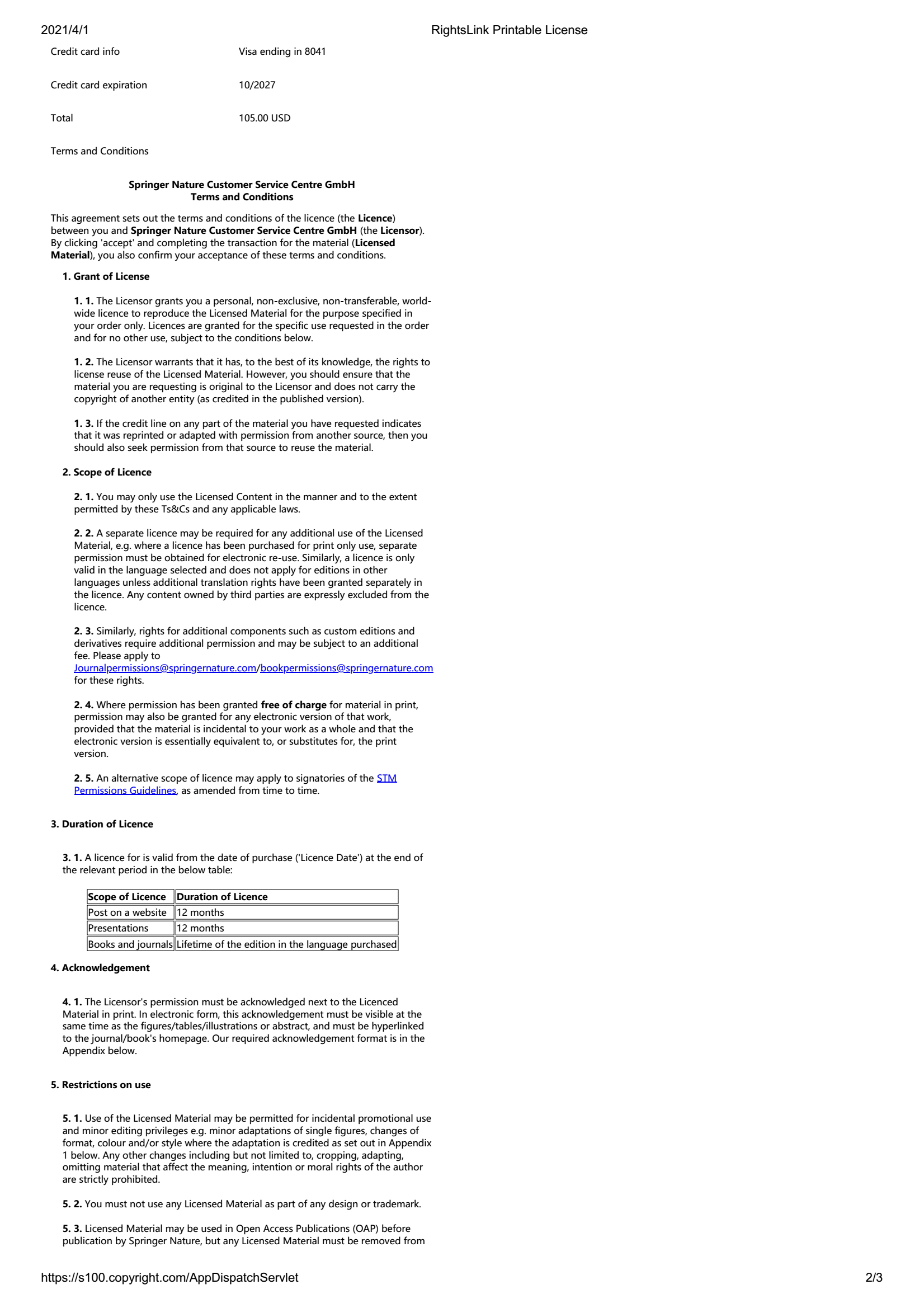

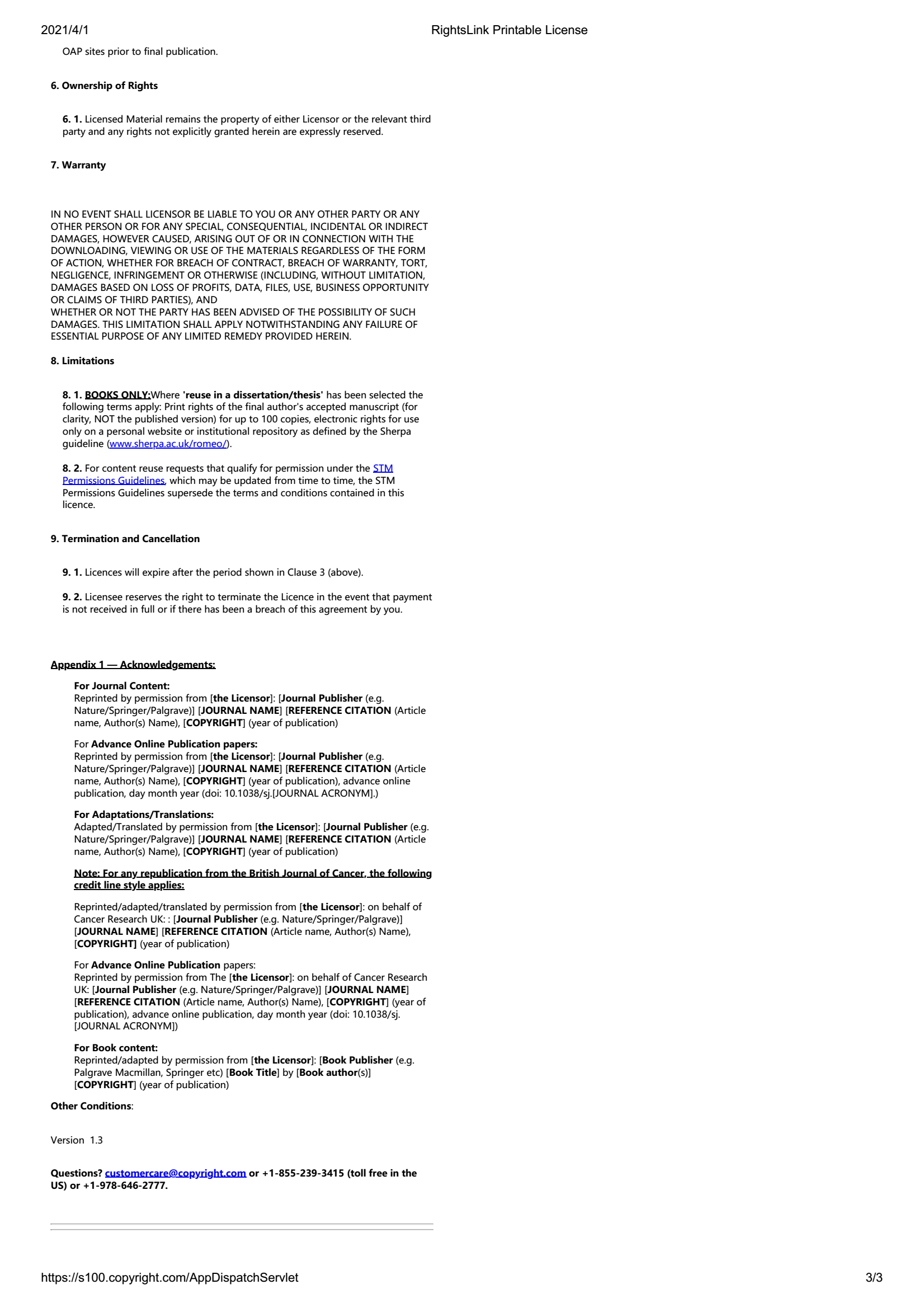


For Fig. 3j


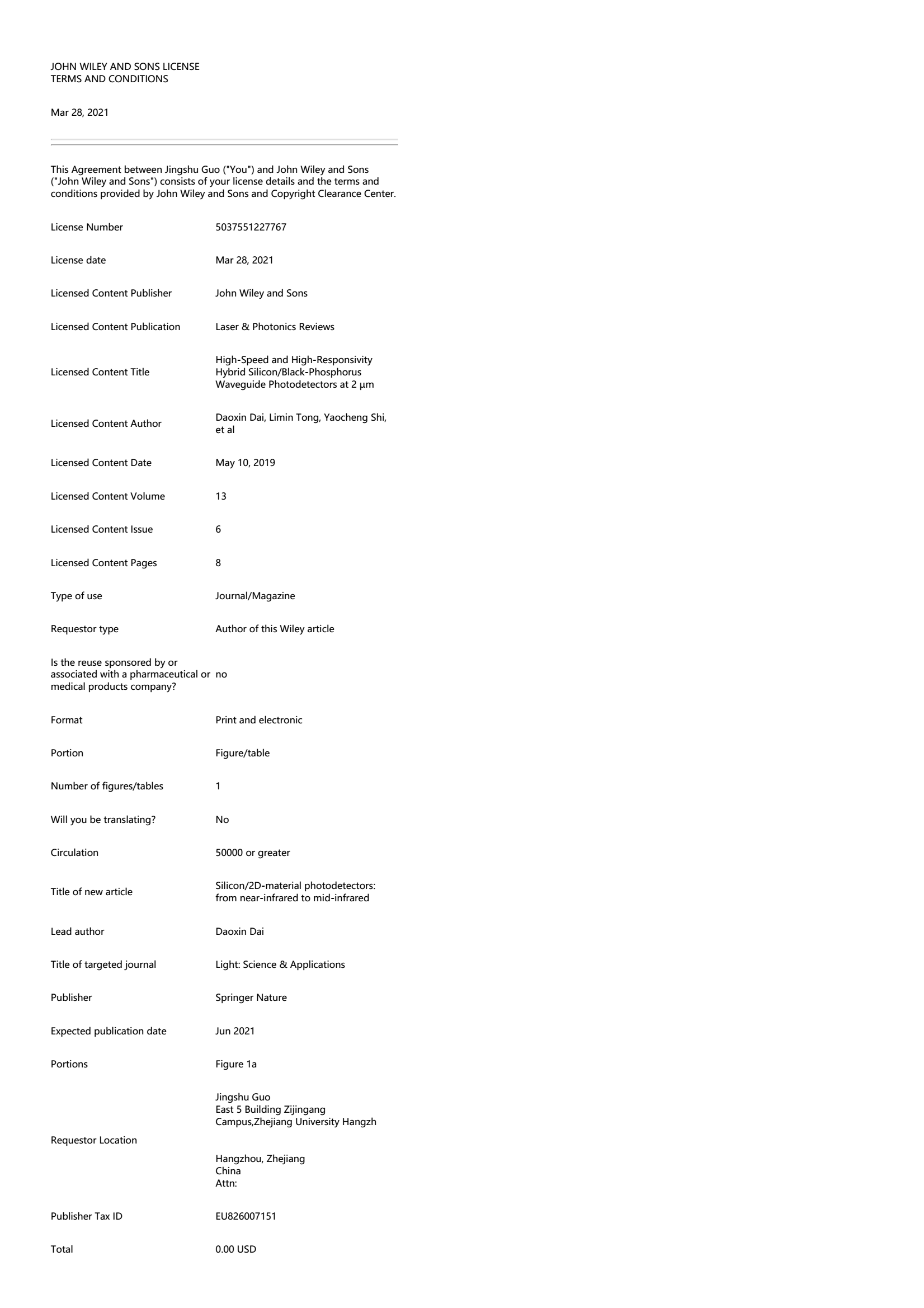

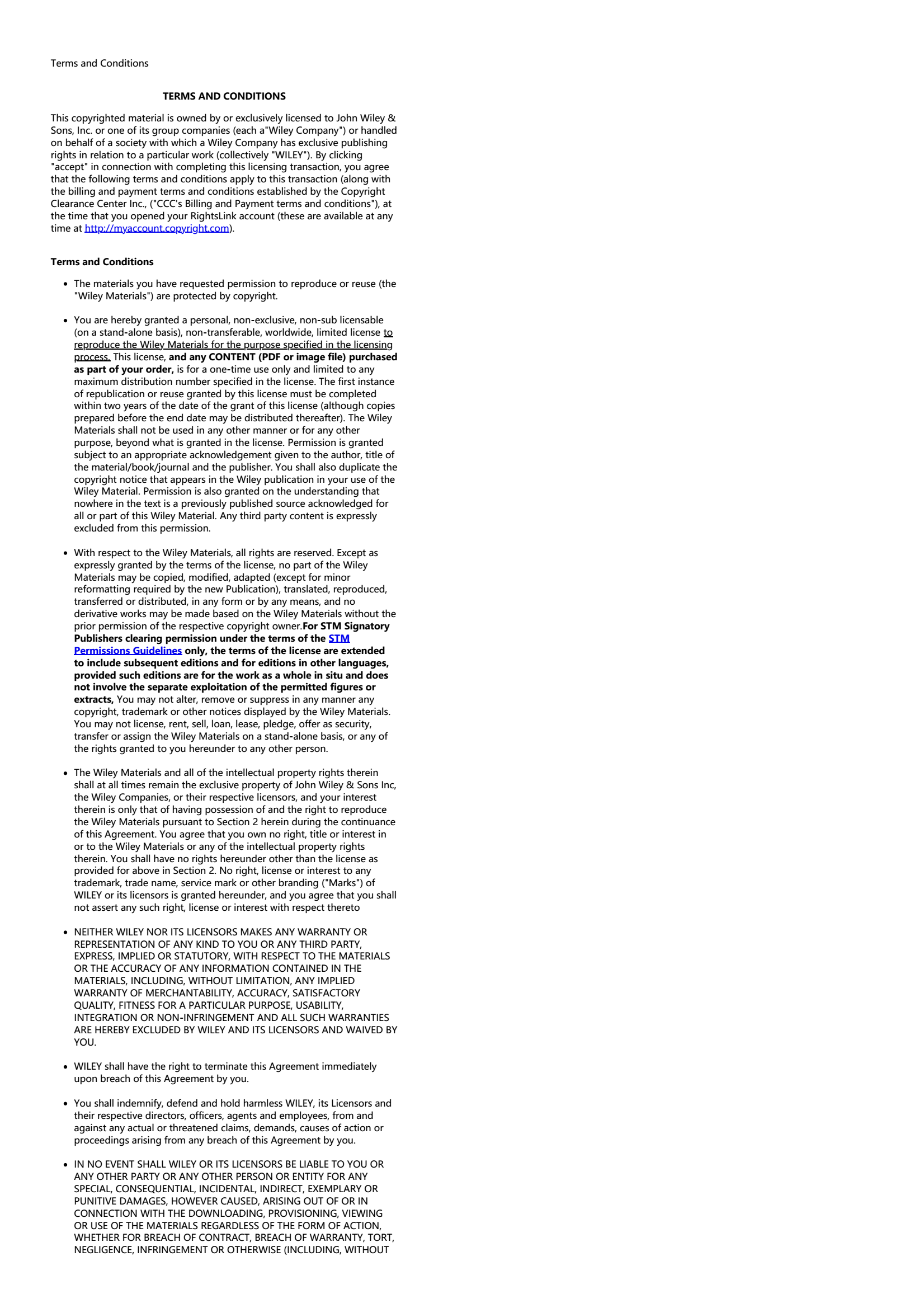


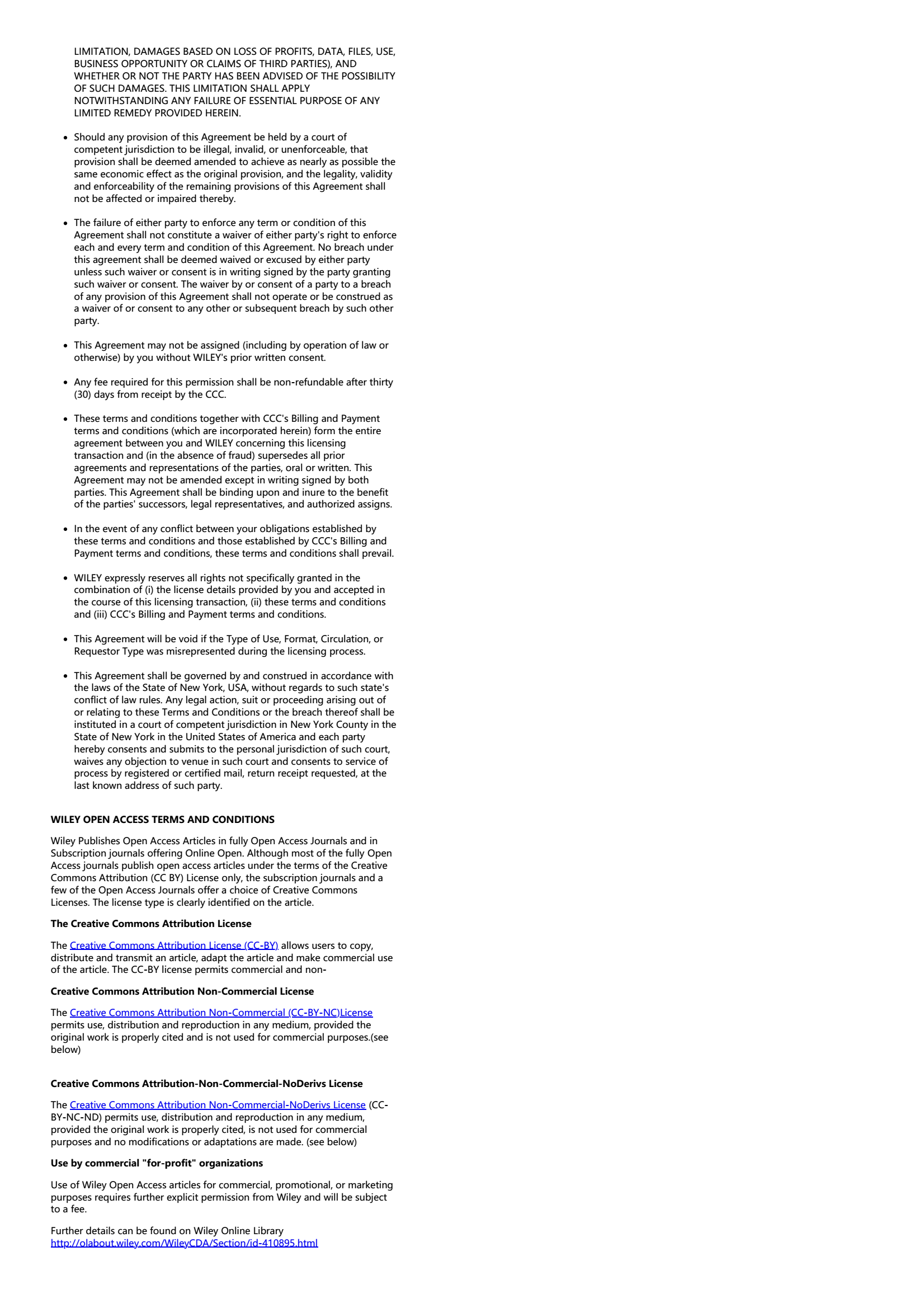

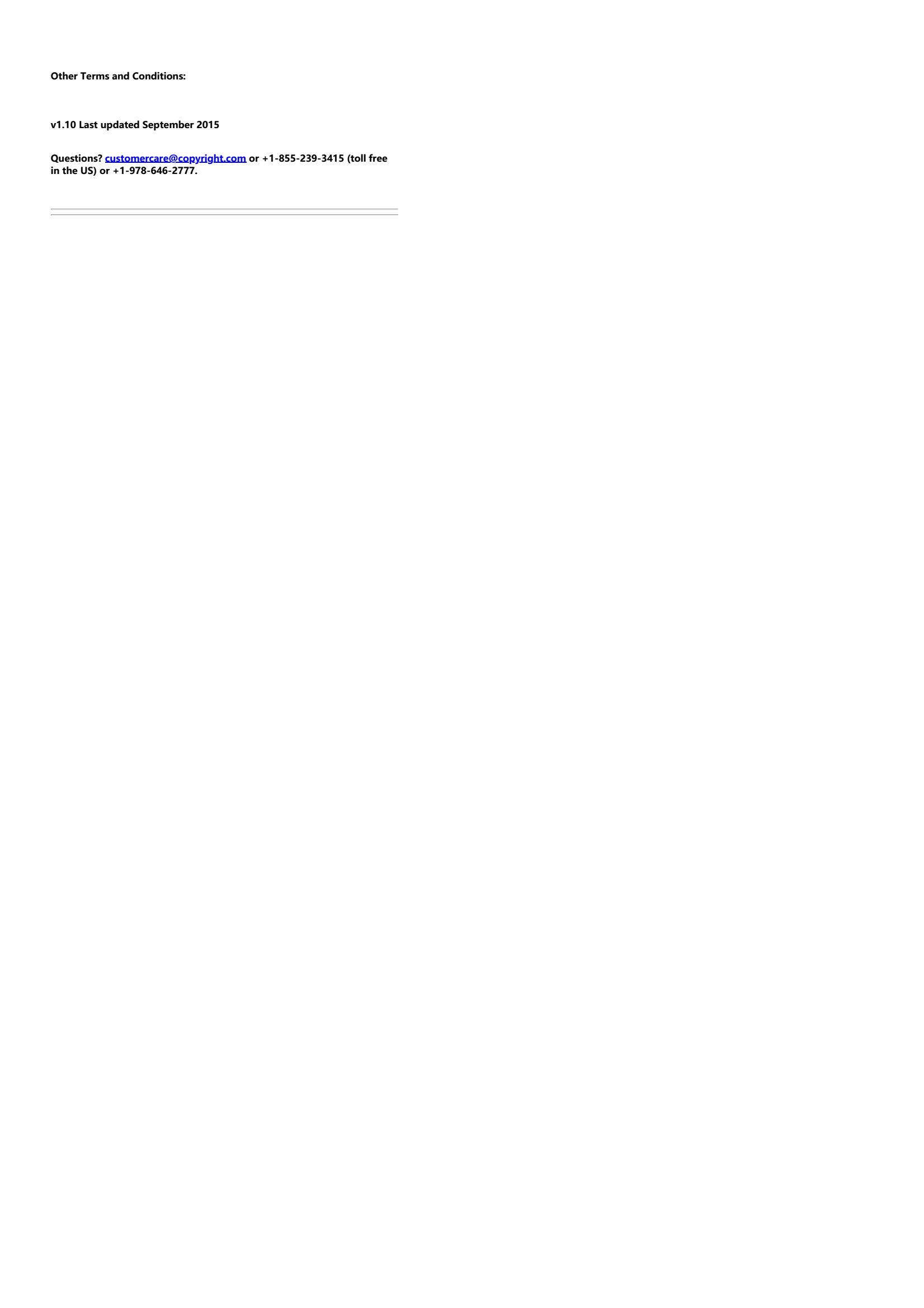


For Fig 3k:


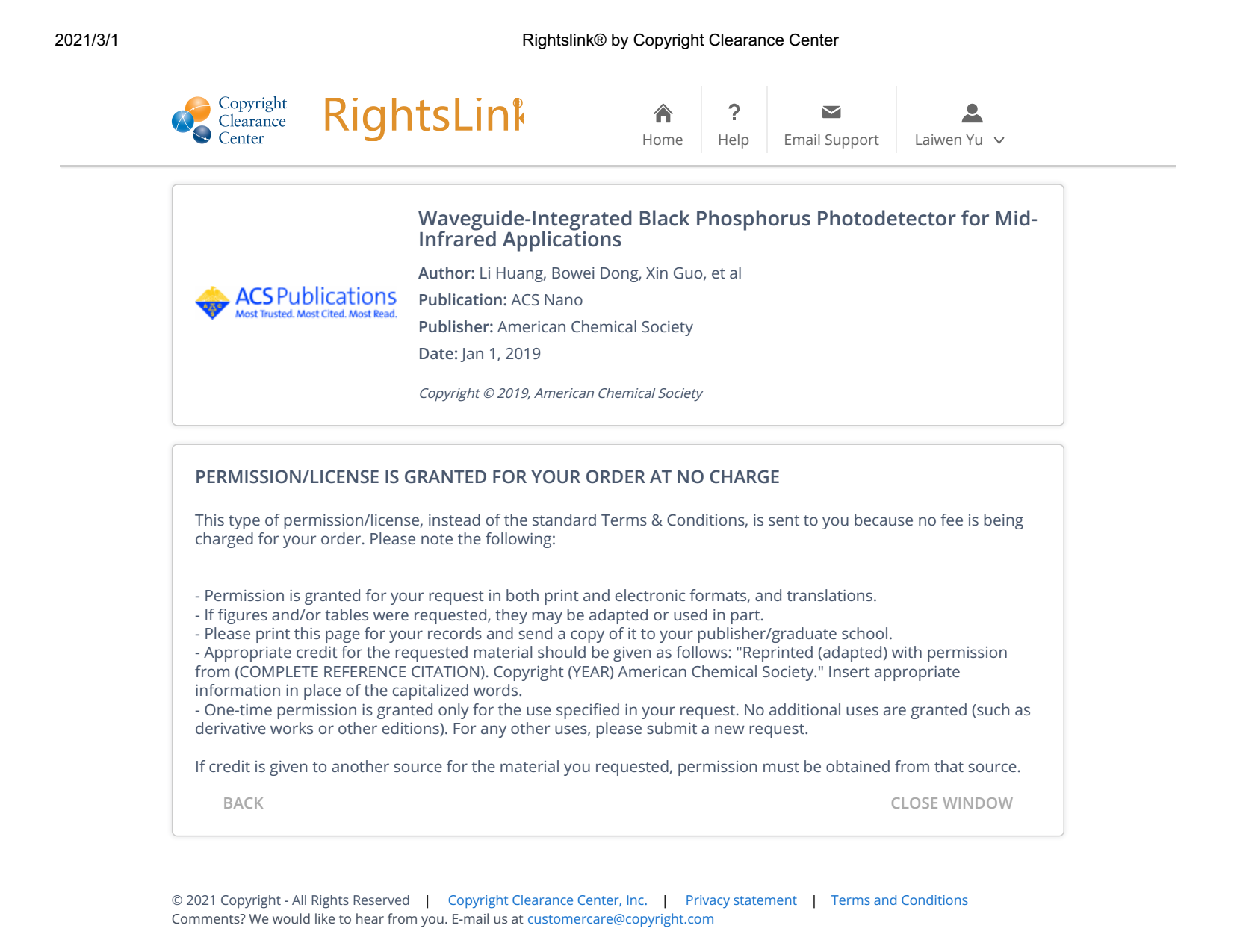


For Fig. 4c


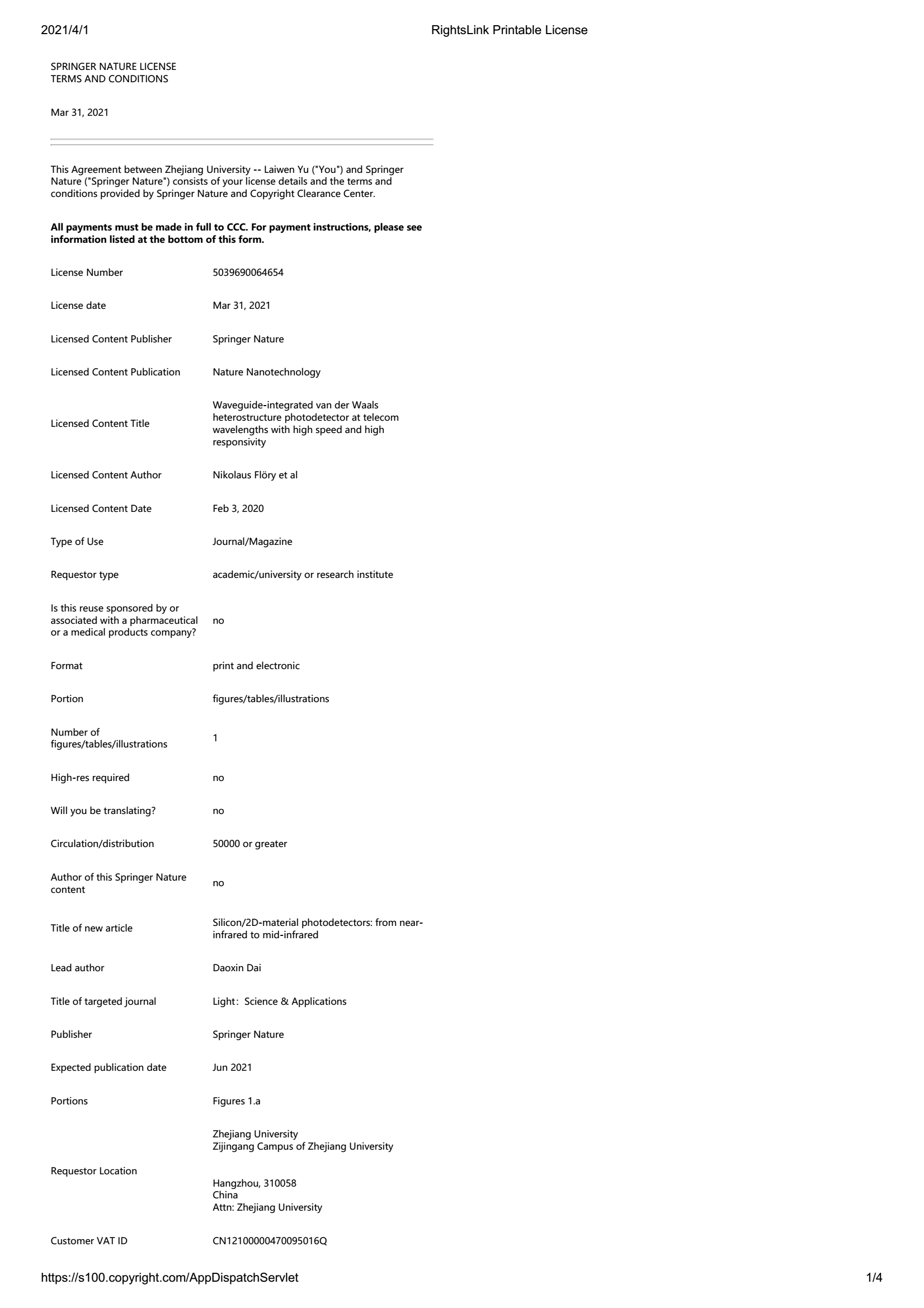

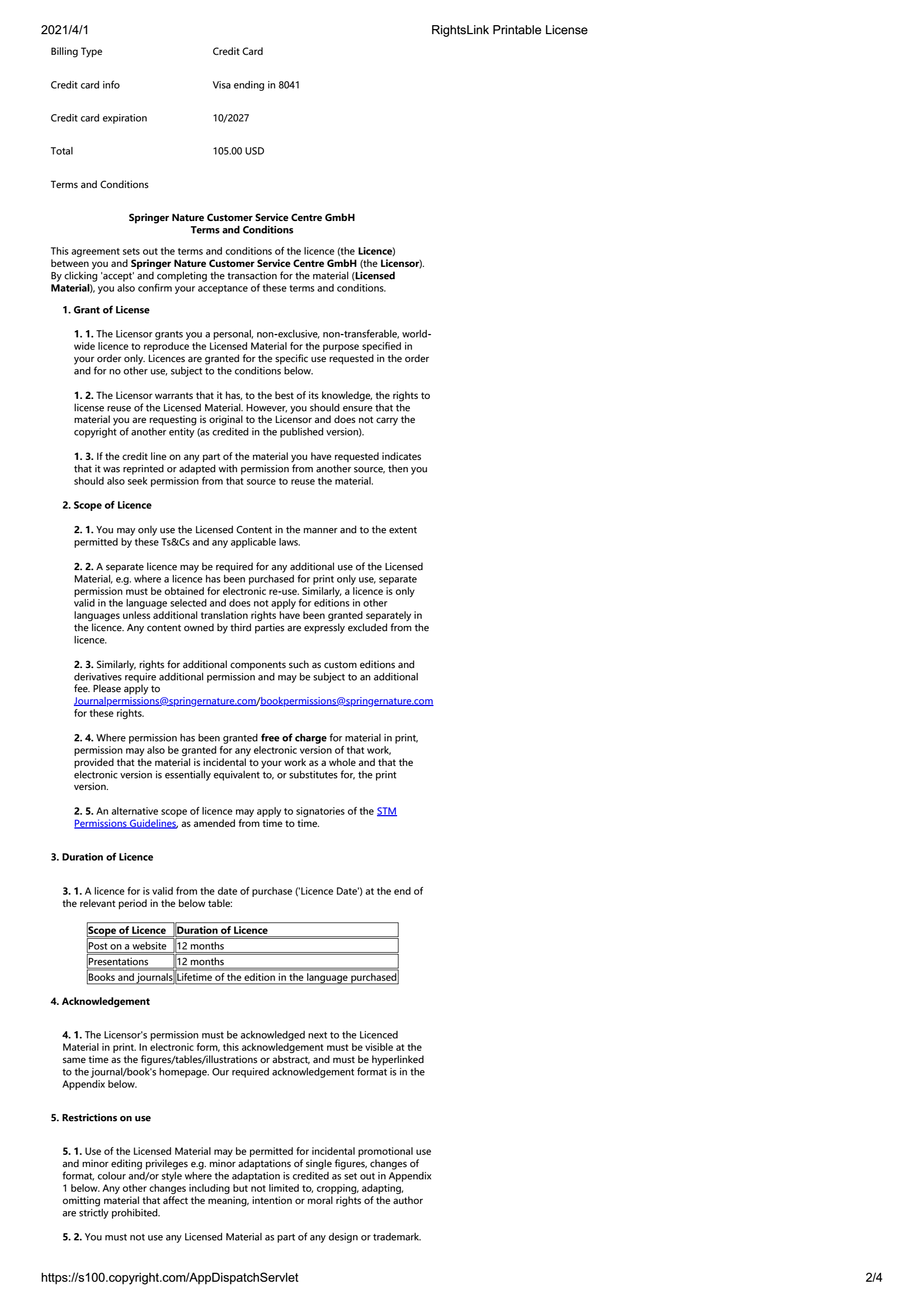

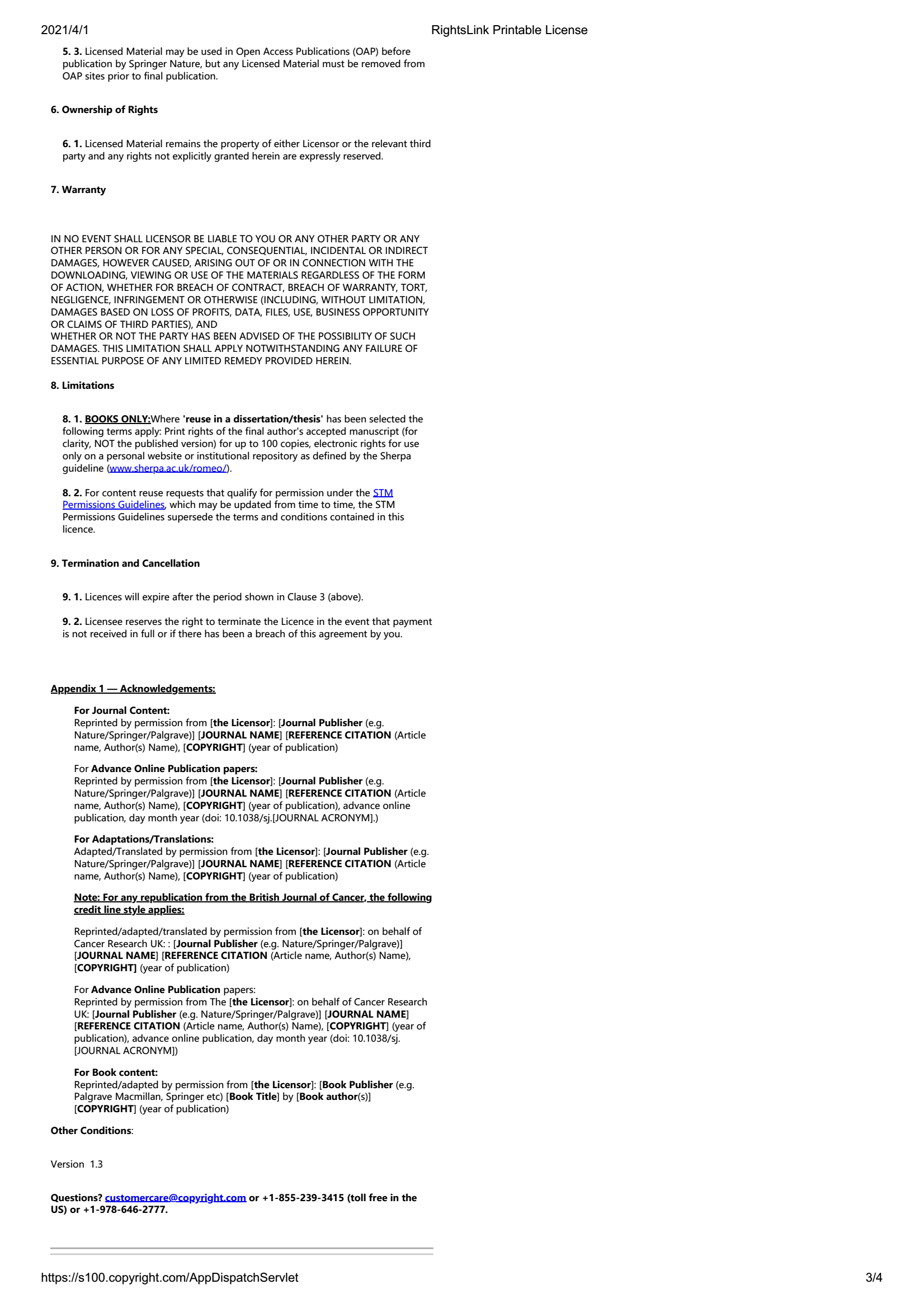


For Fig. 6b


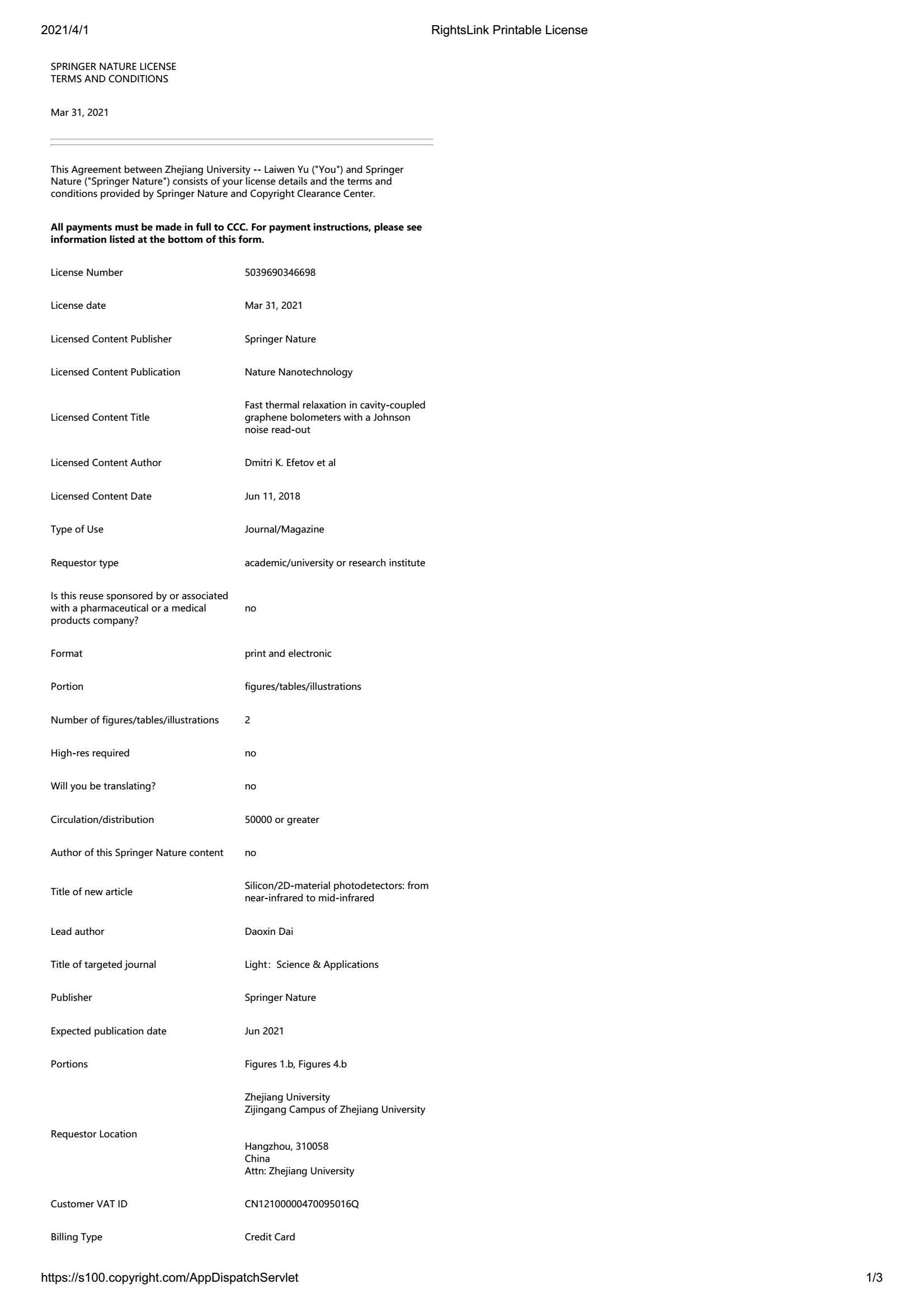

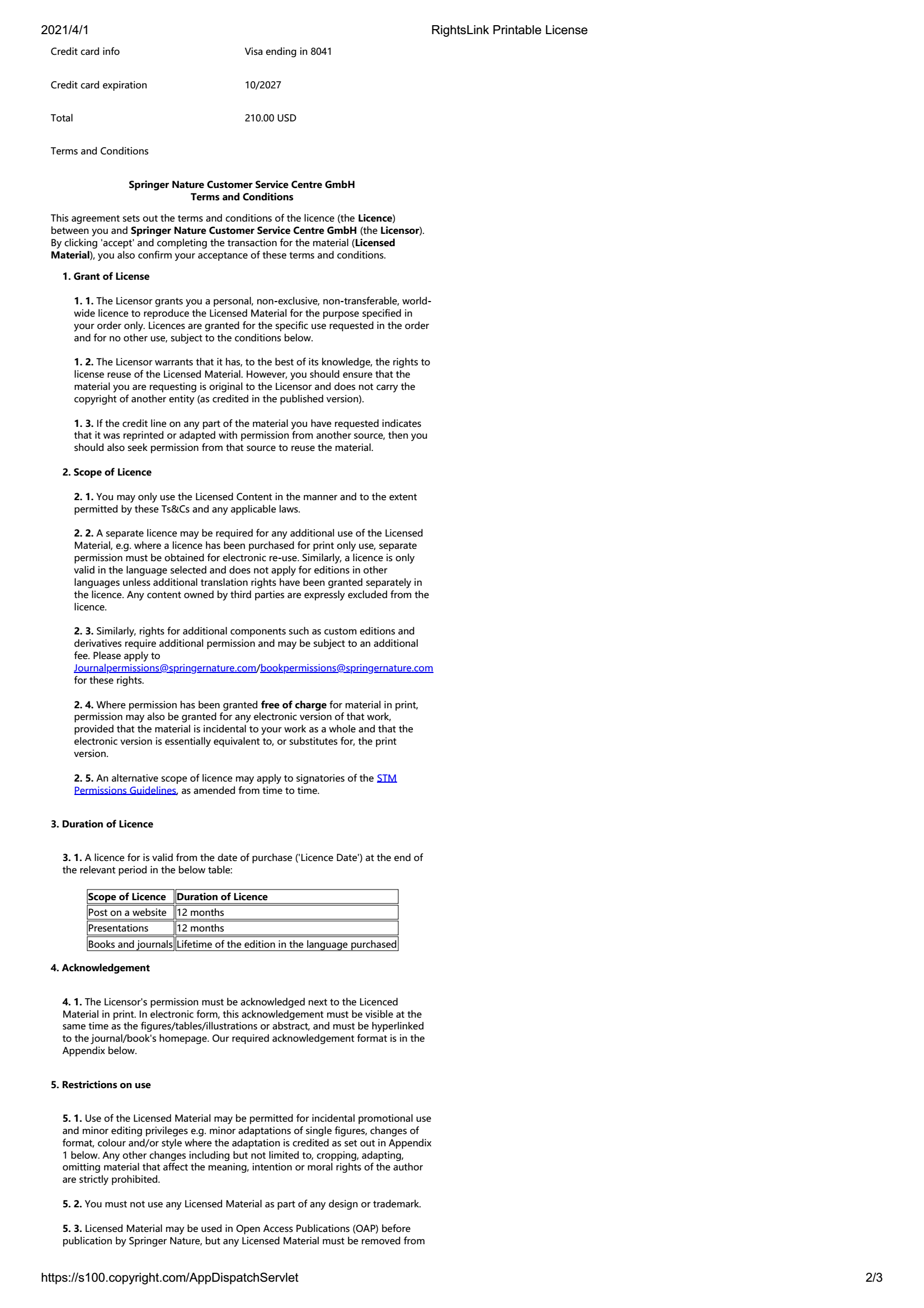


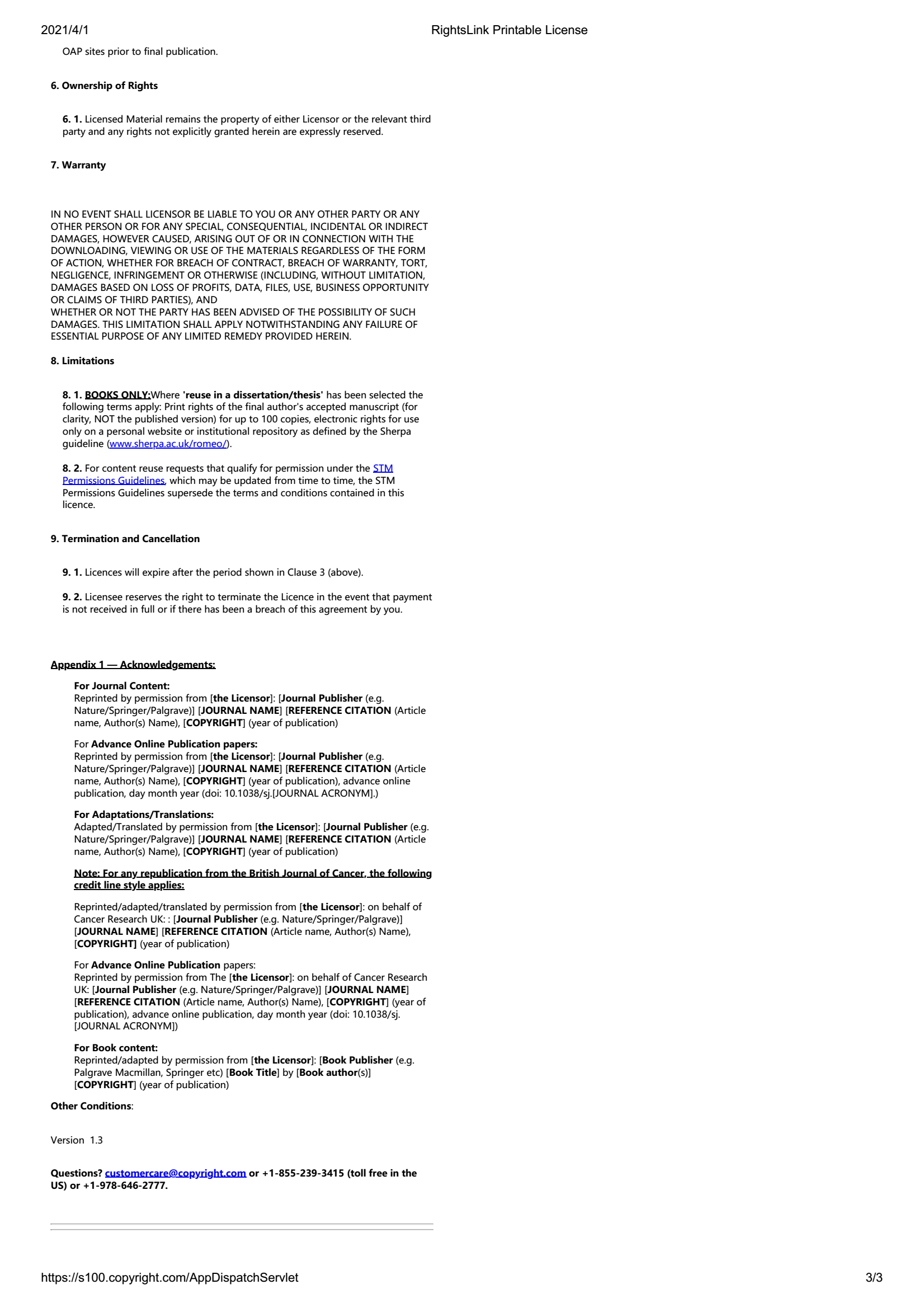


For Fig. 6d


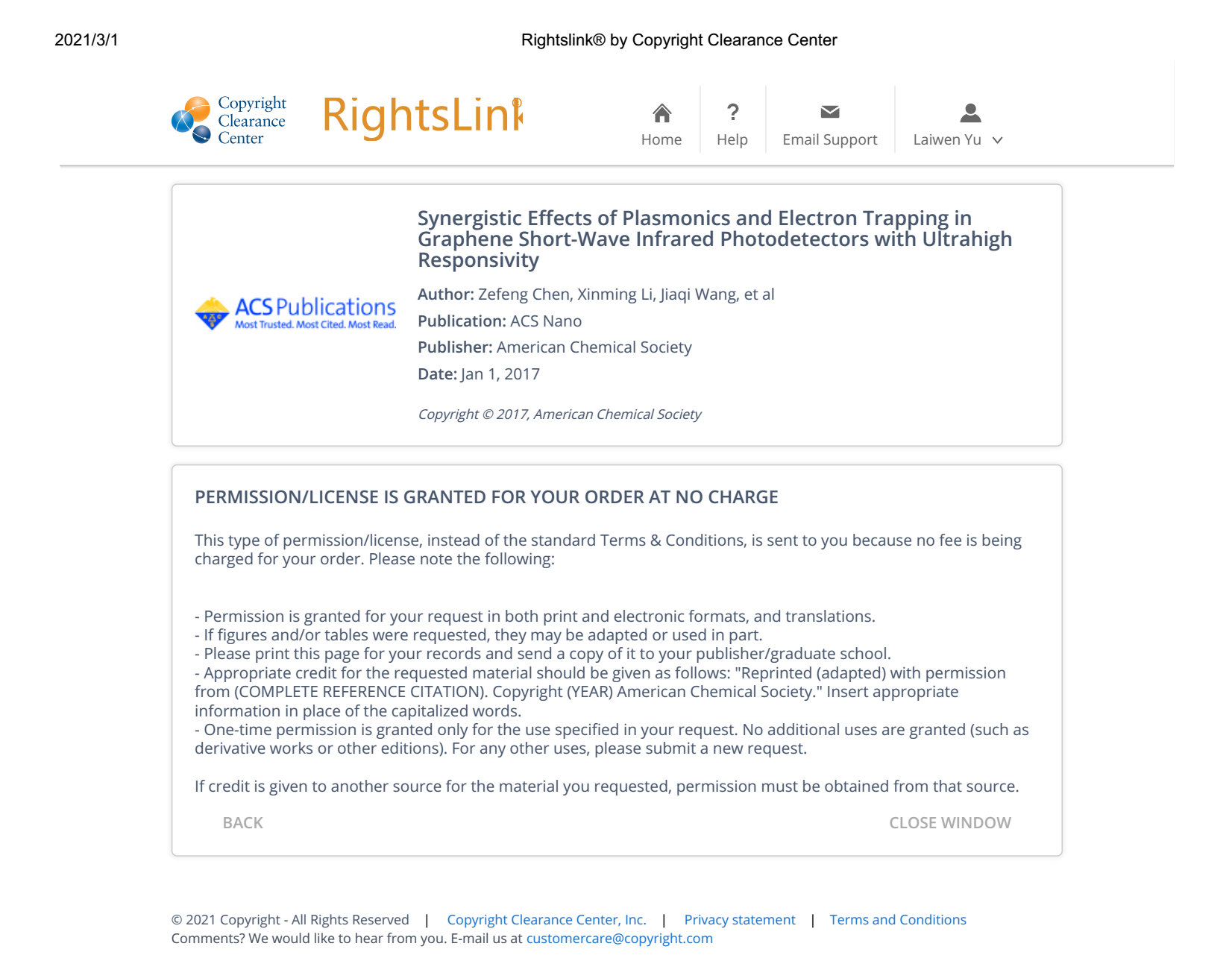


For Fig. 6e


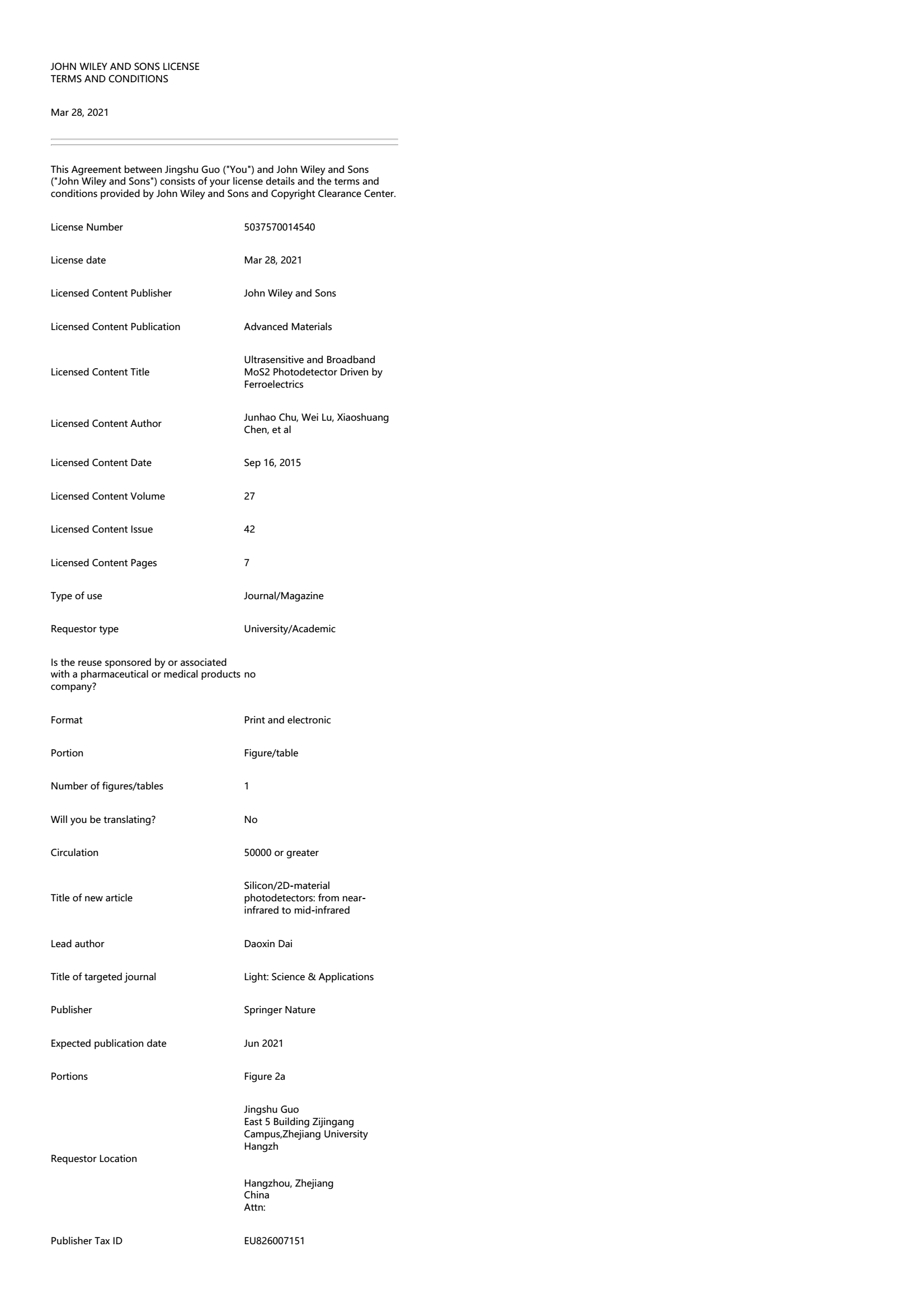

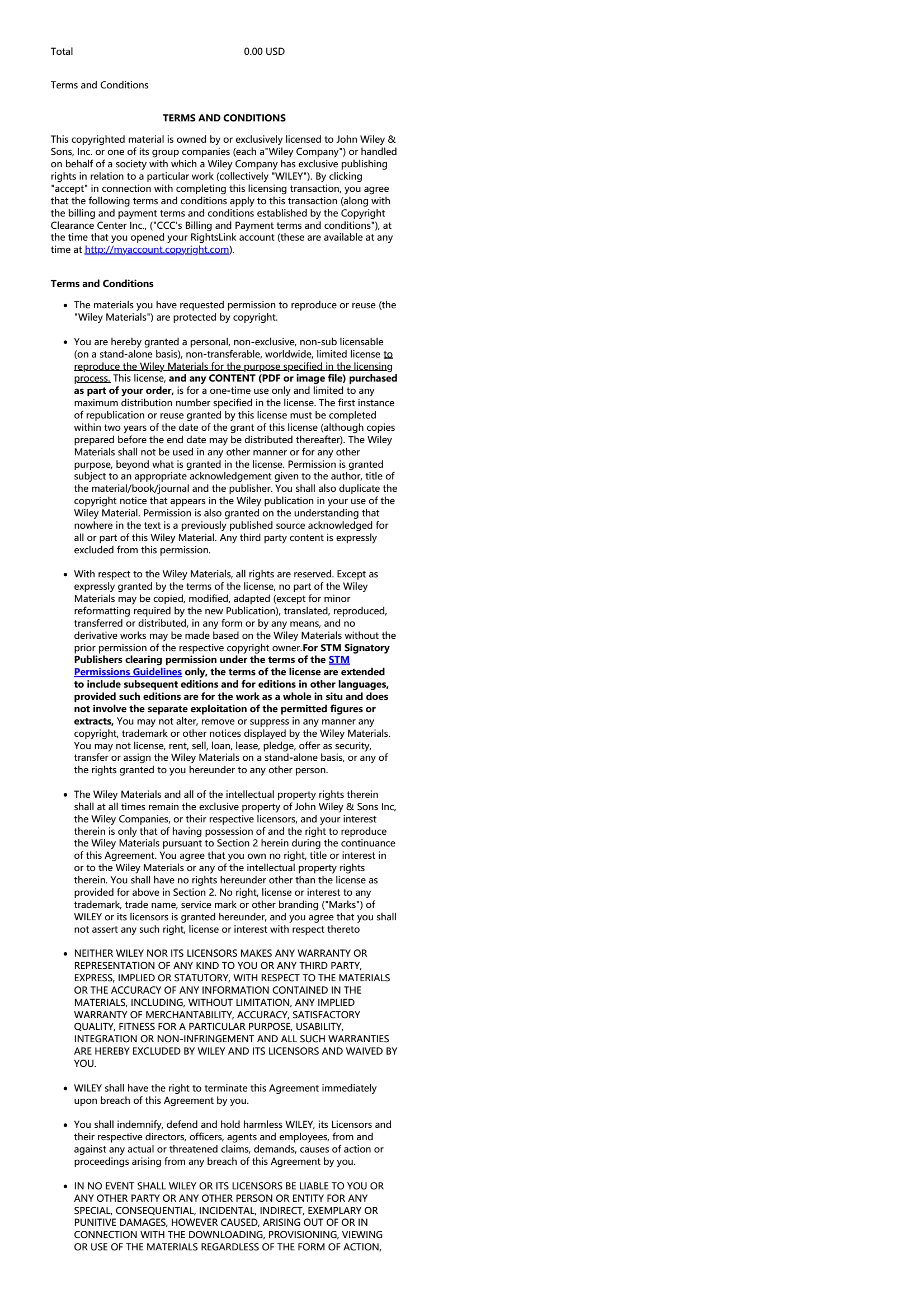


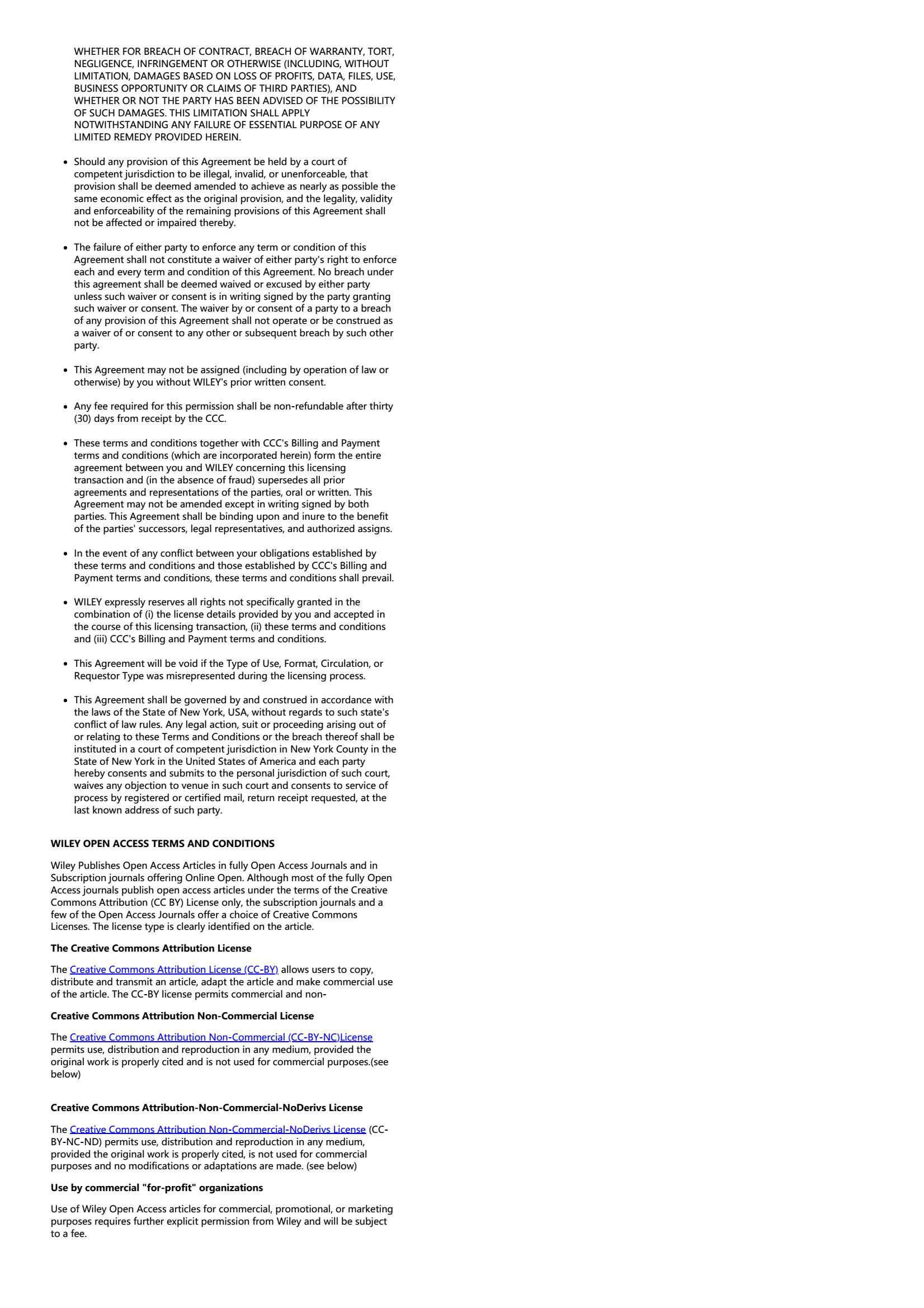

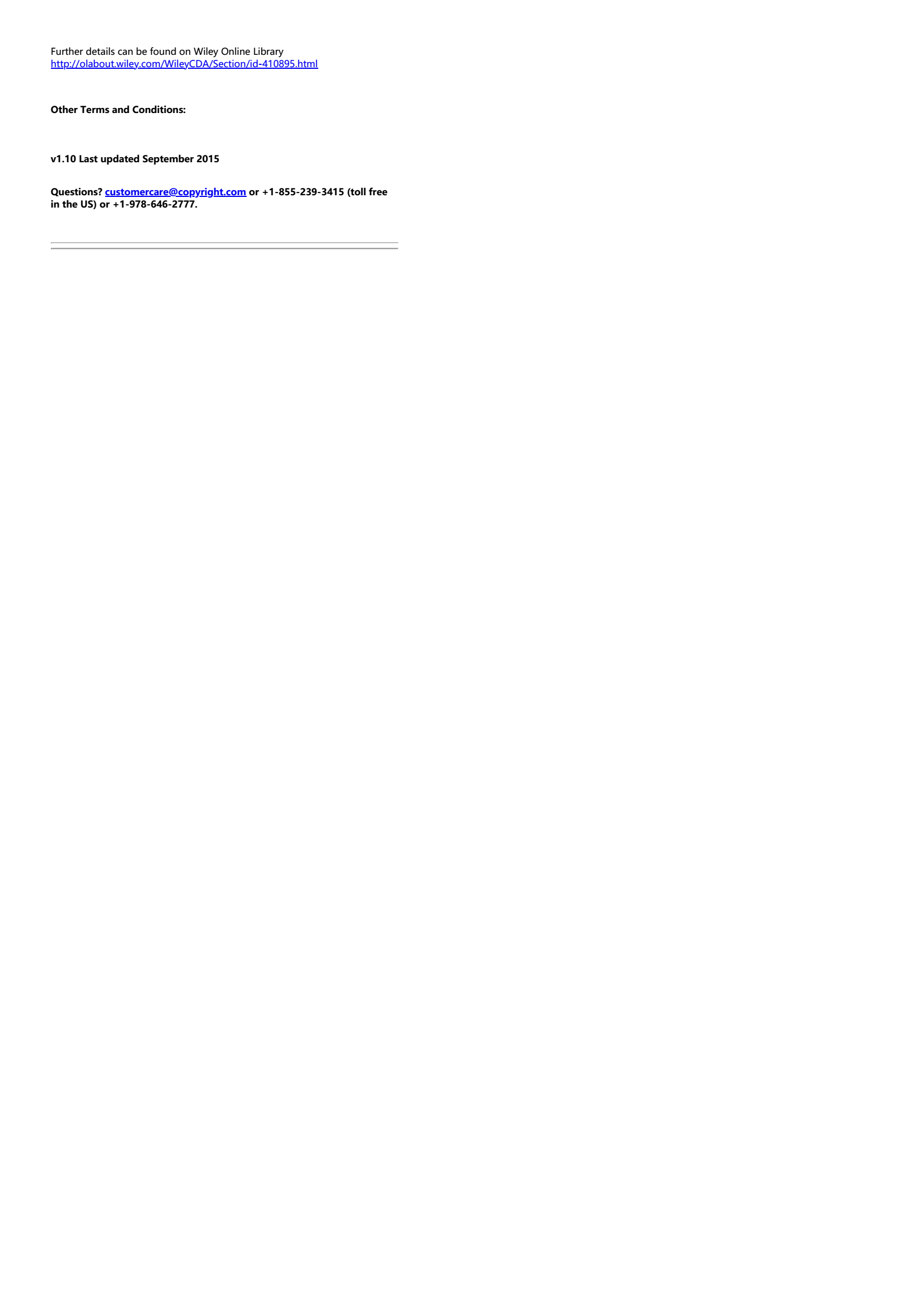


For Fig. 6f


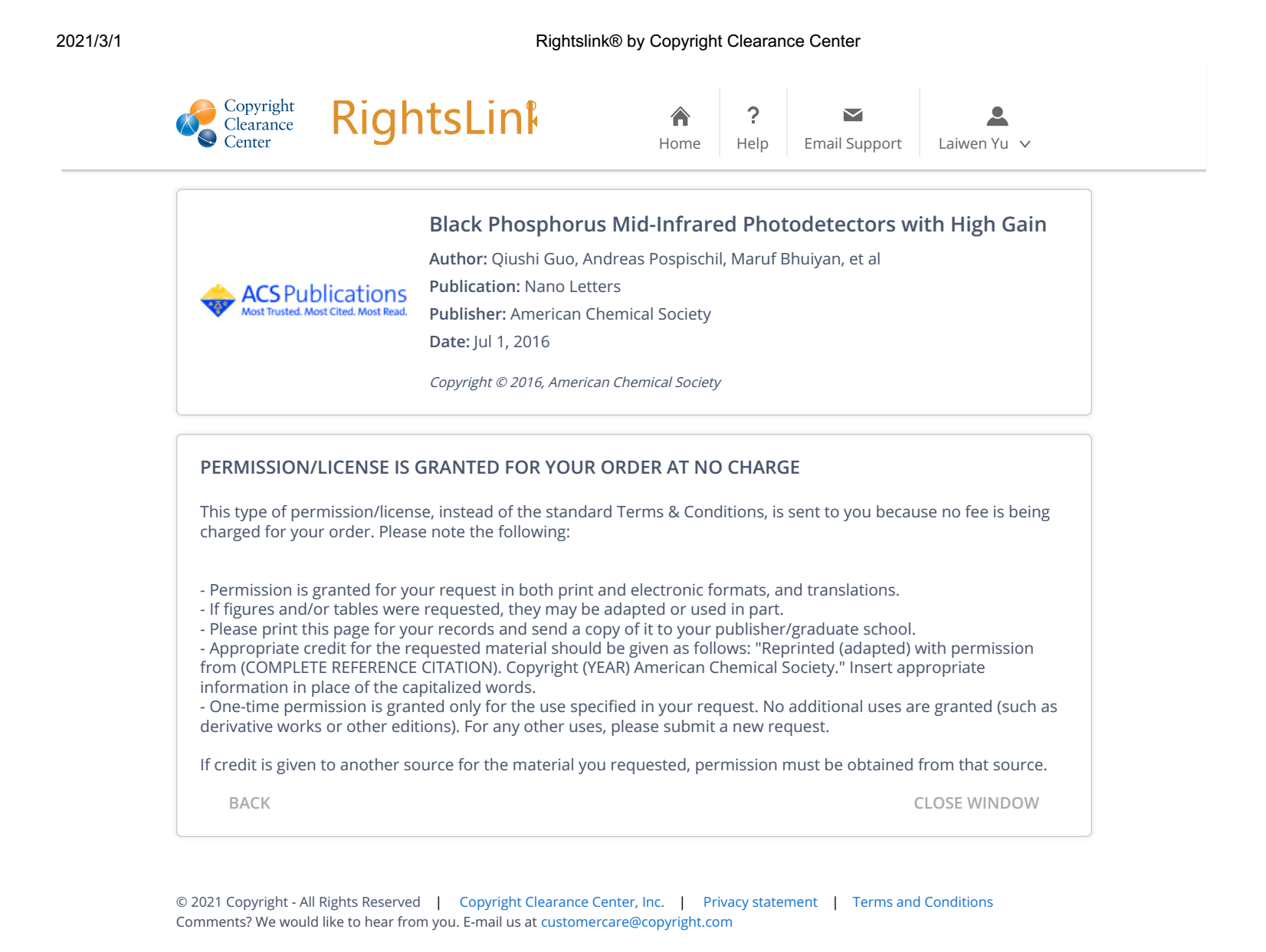


For Fig. 6h


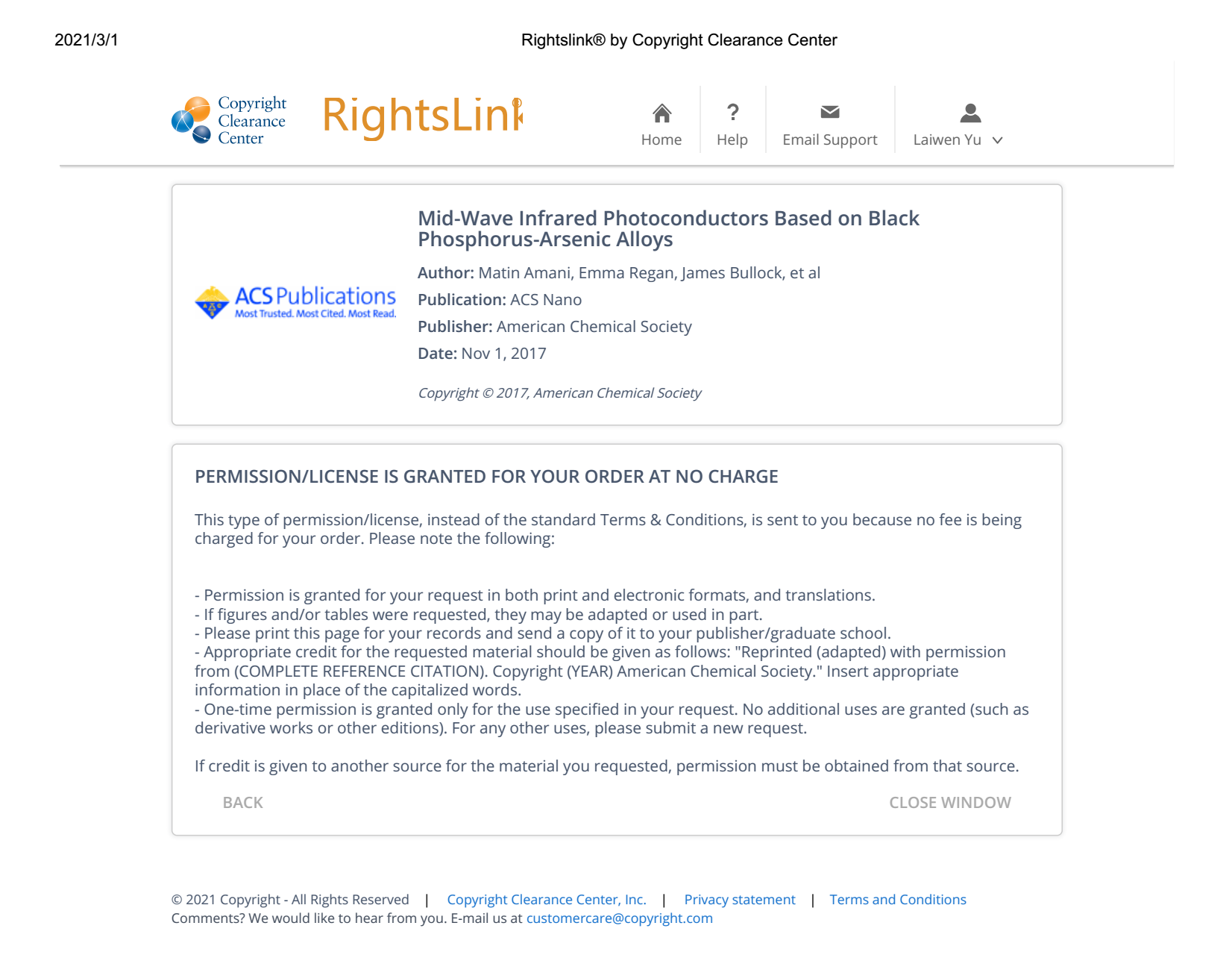


For Fig. 6i


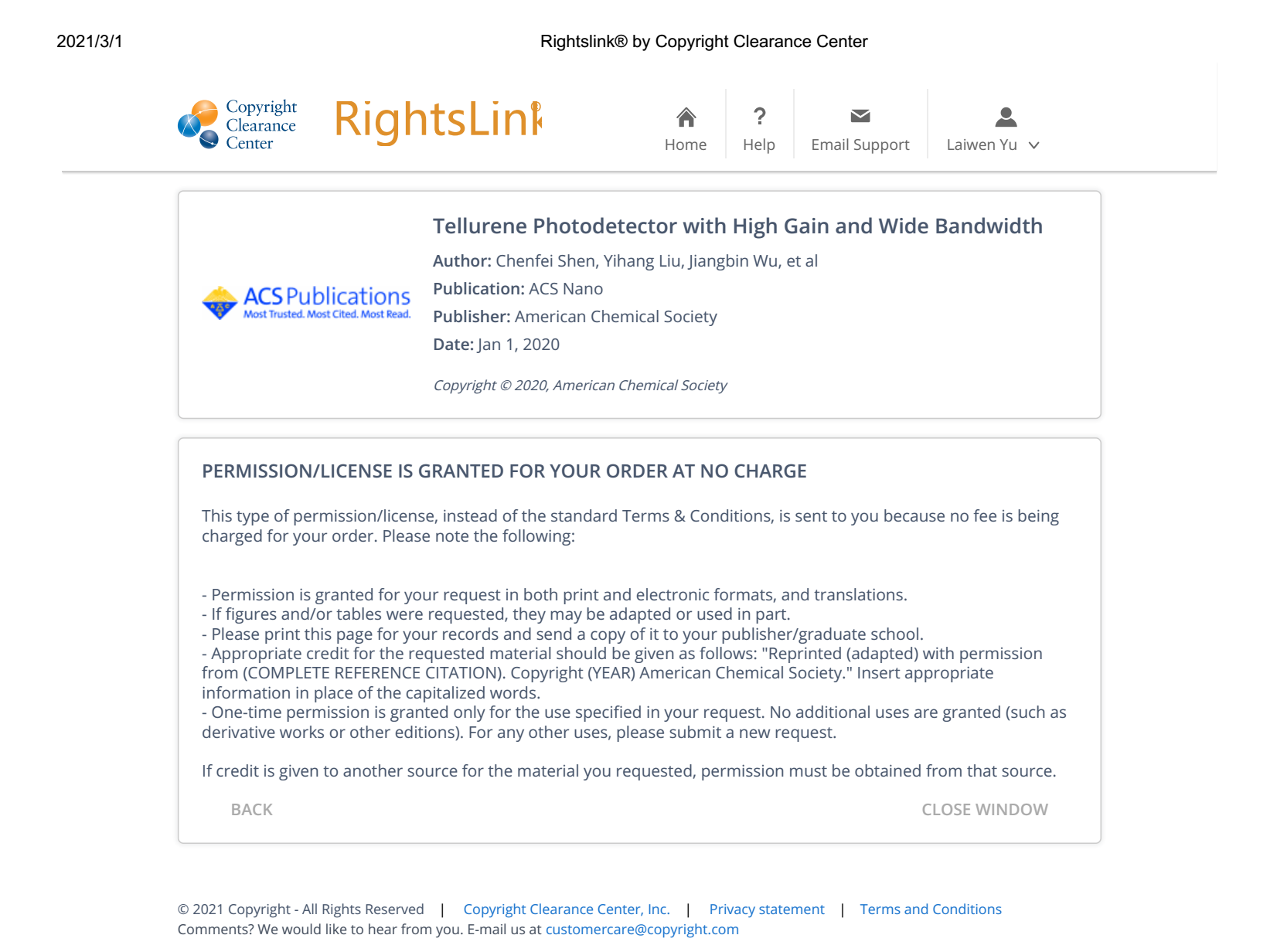


For Fig. 7a


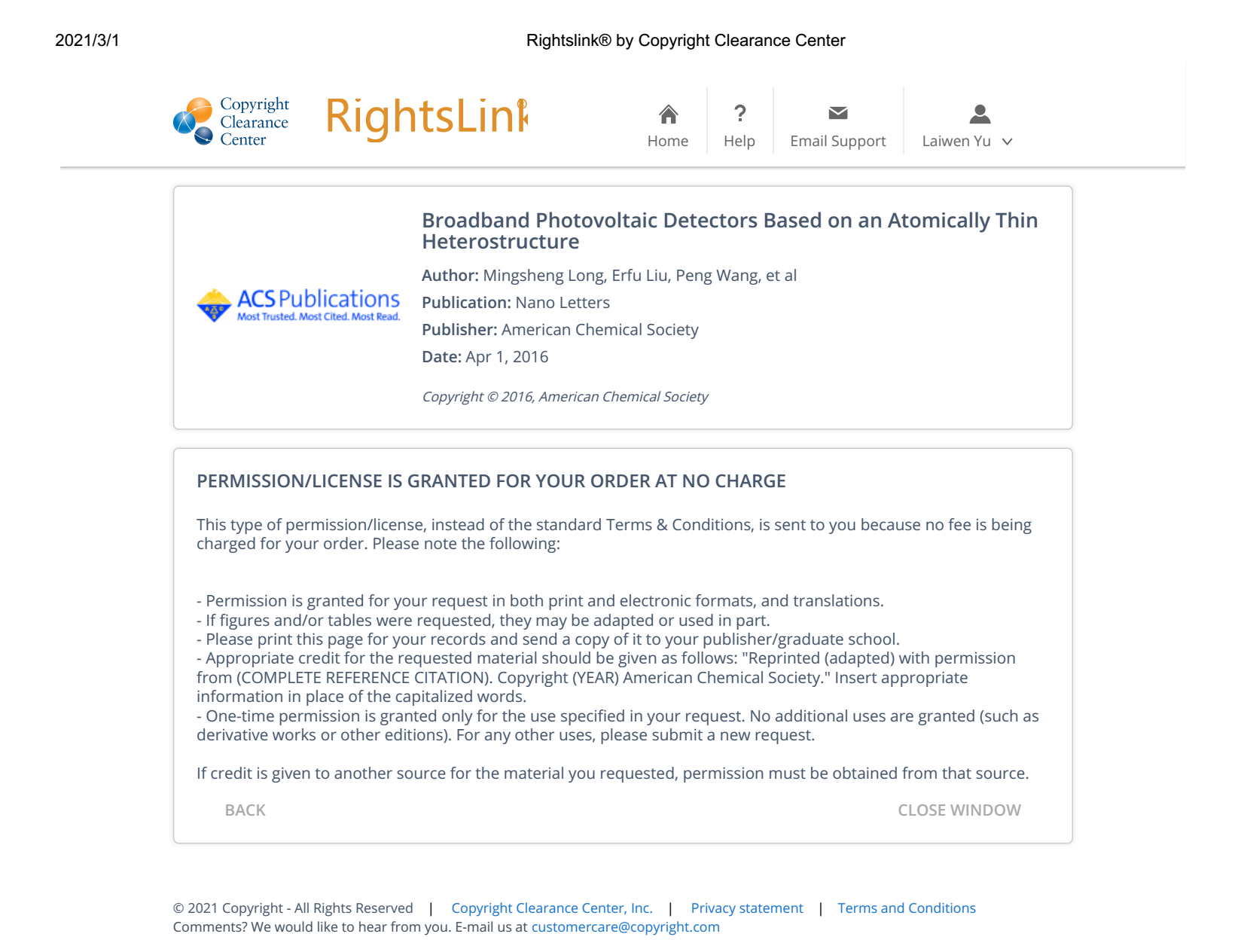


For Fig. 7c


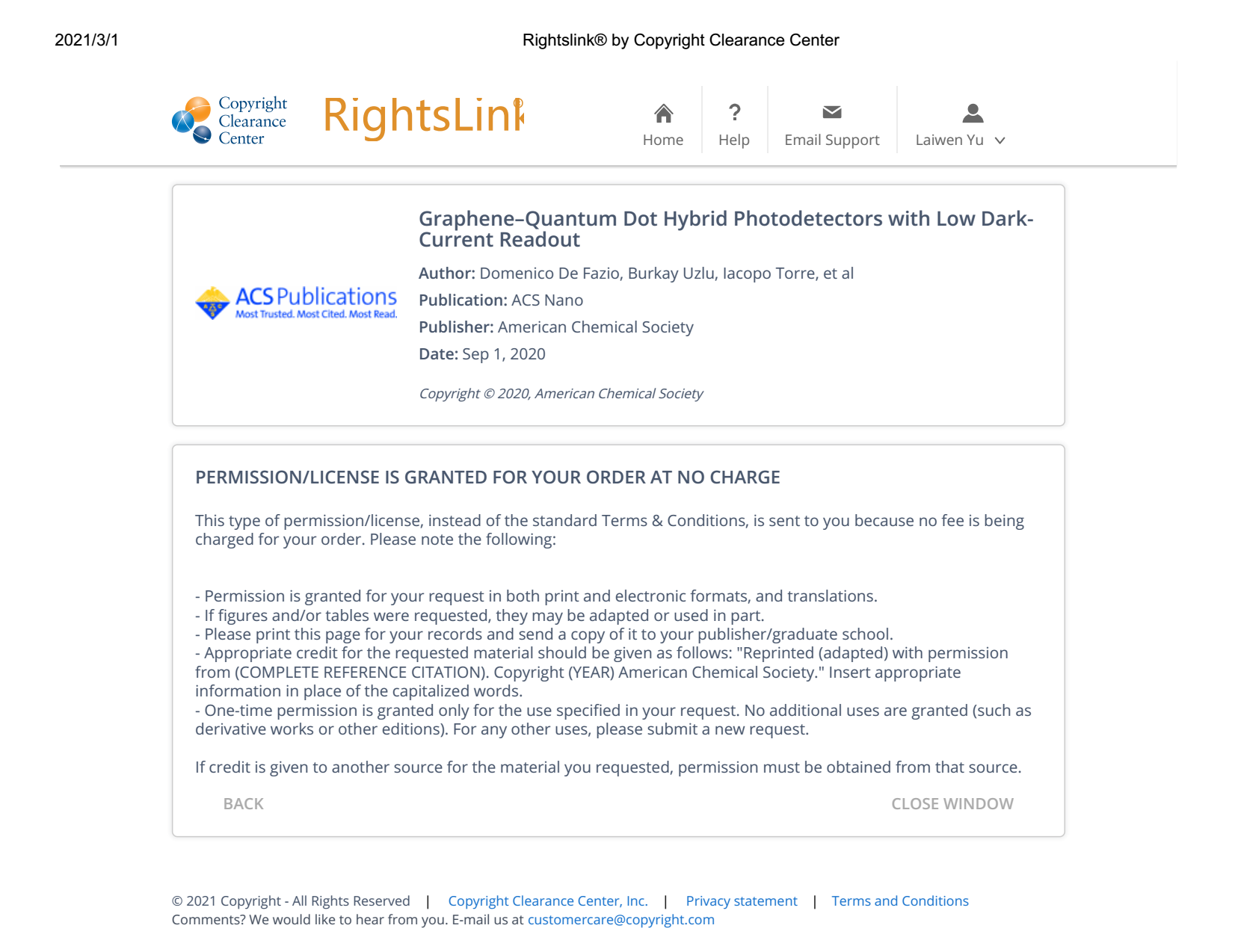


For Fig. 7e


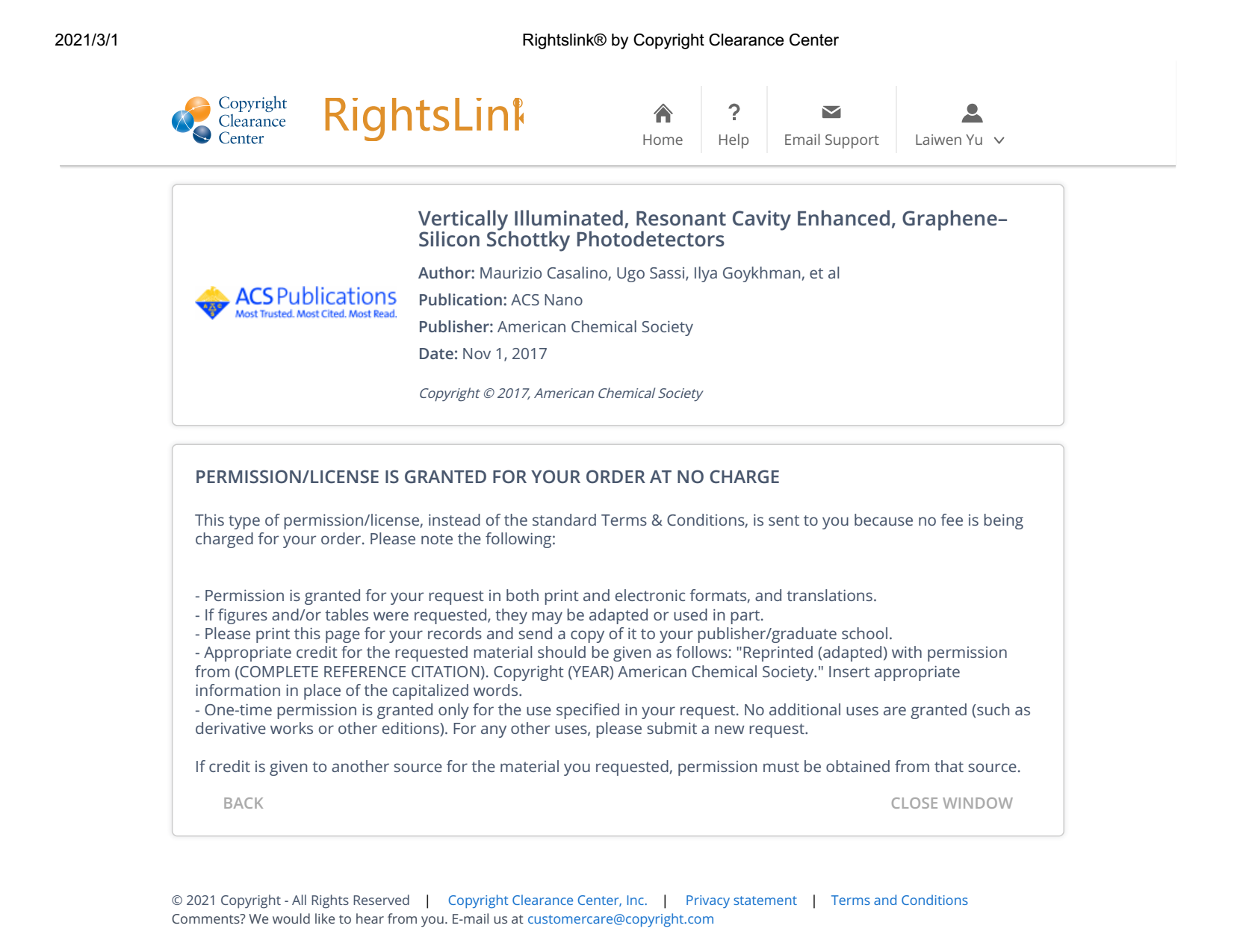


For Fig. 7g


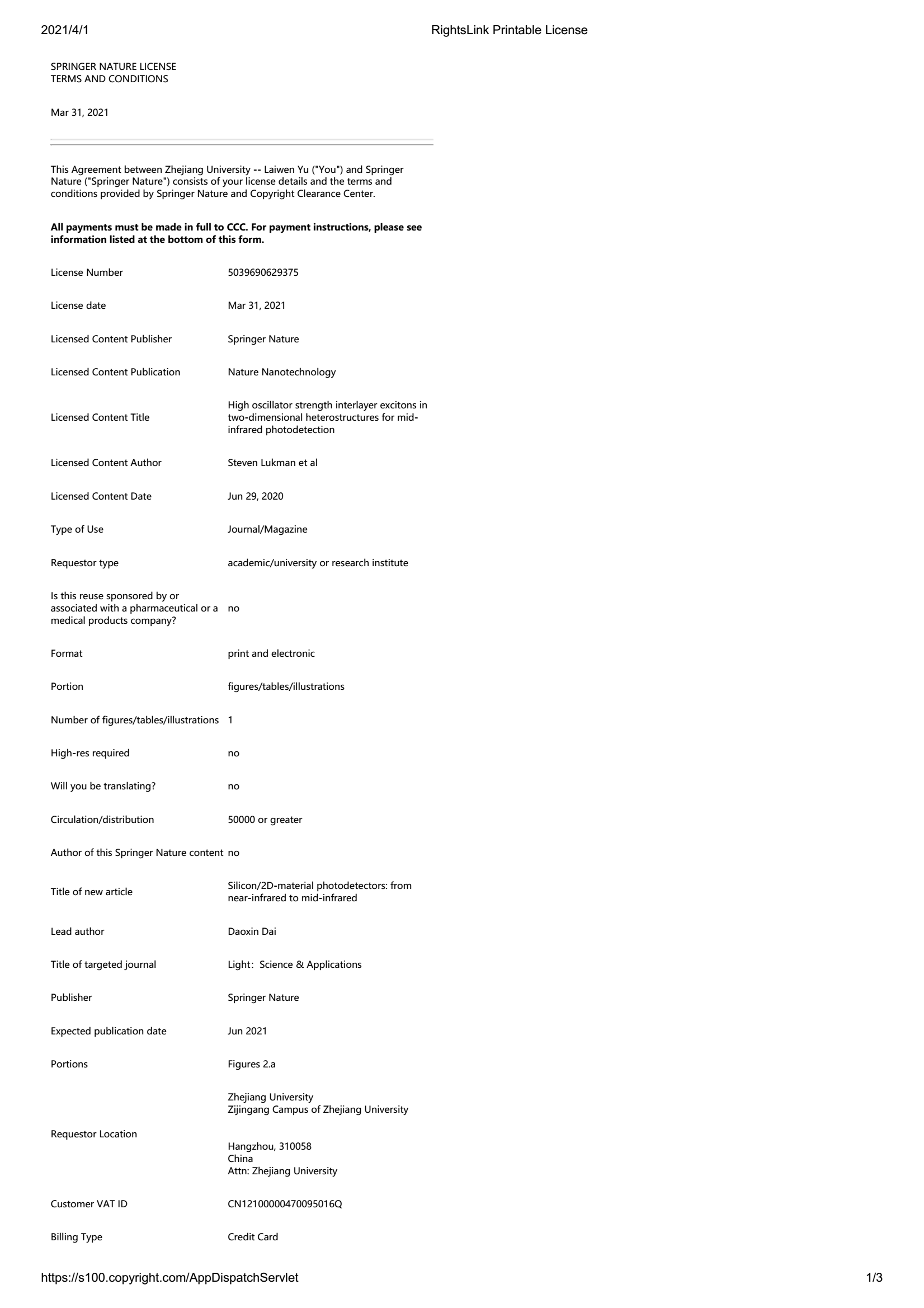

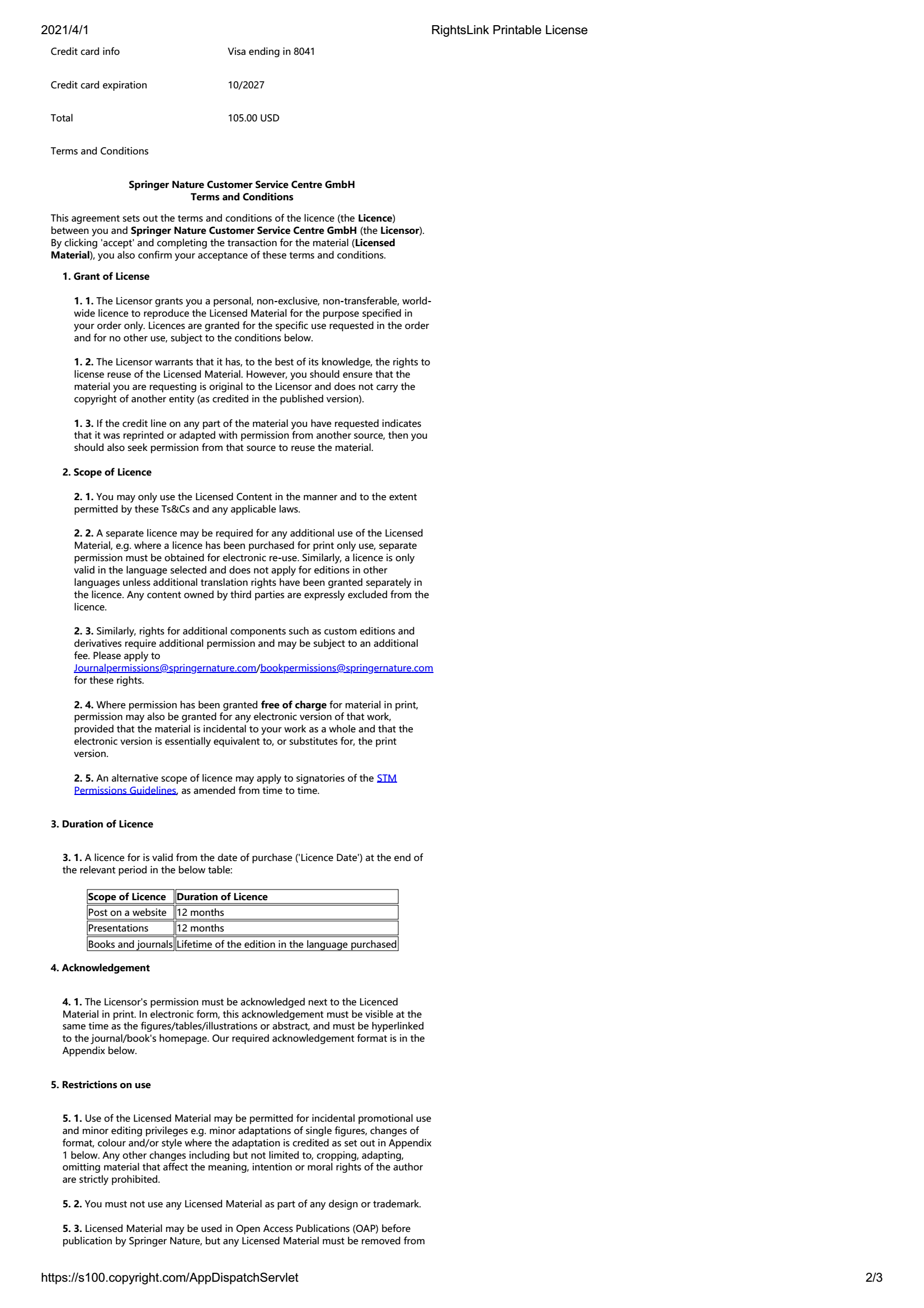

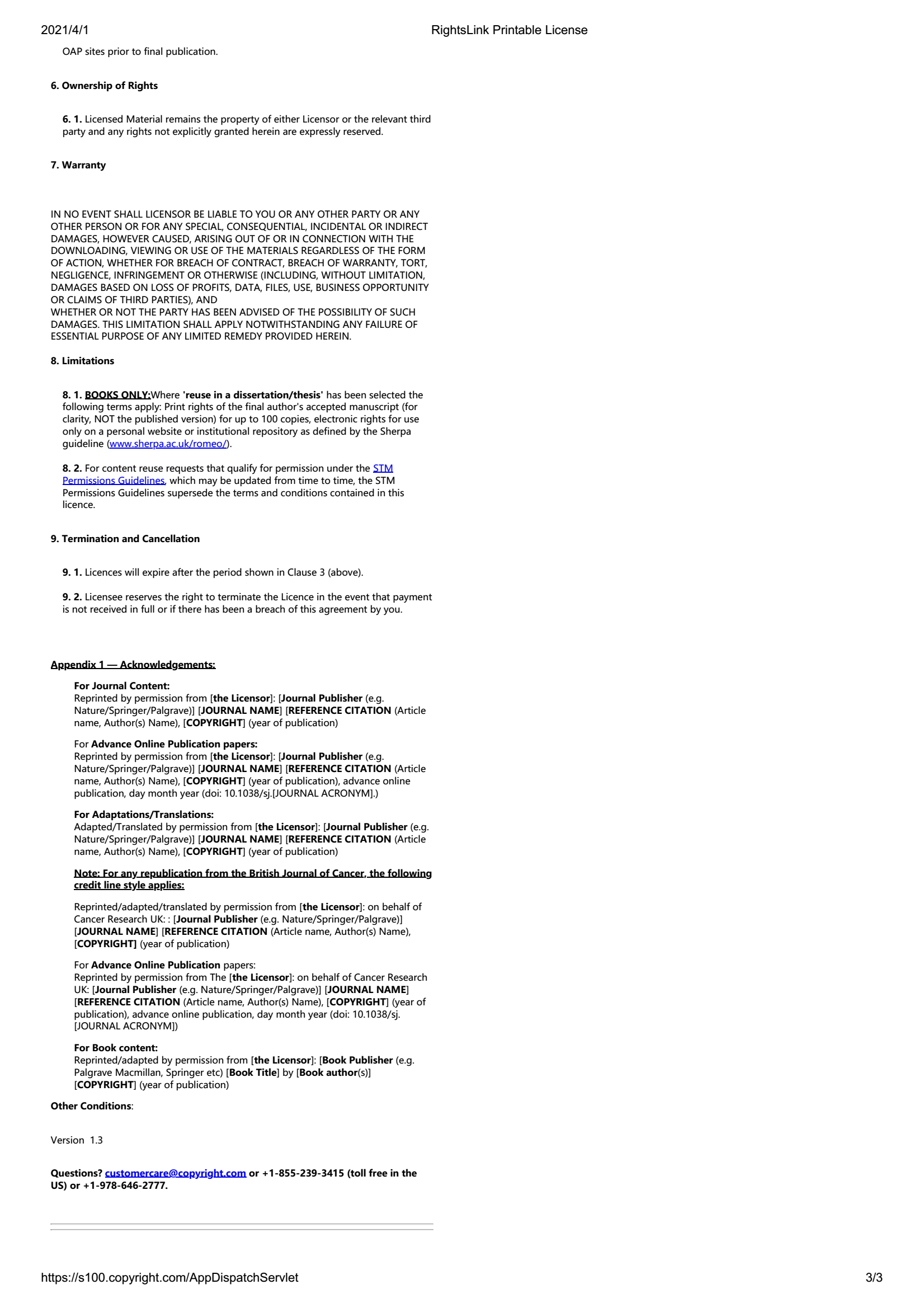


For Fig. 7h


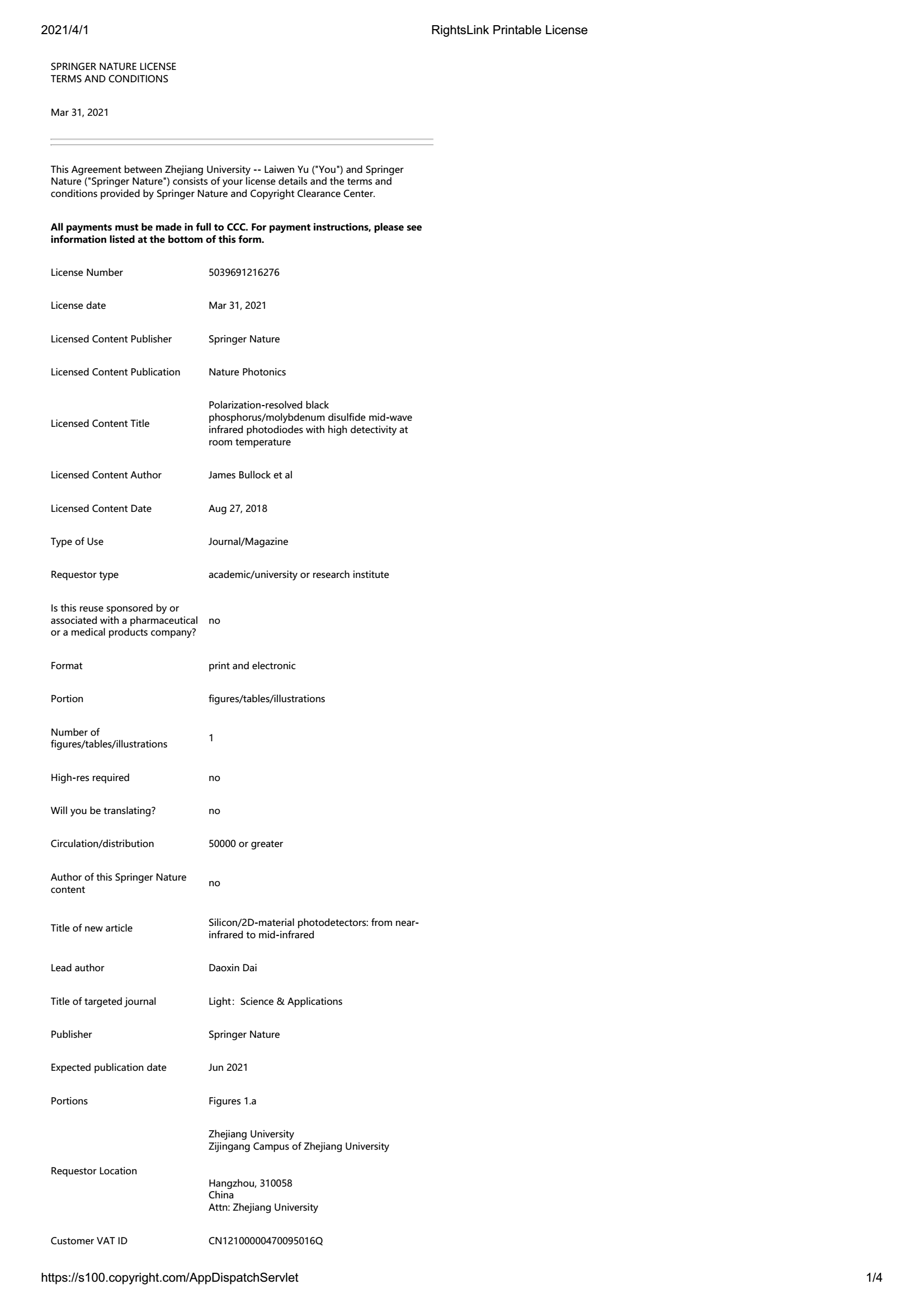

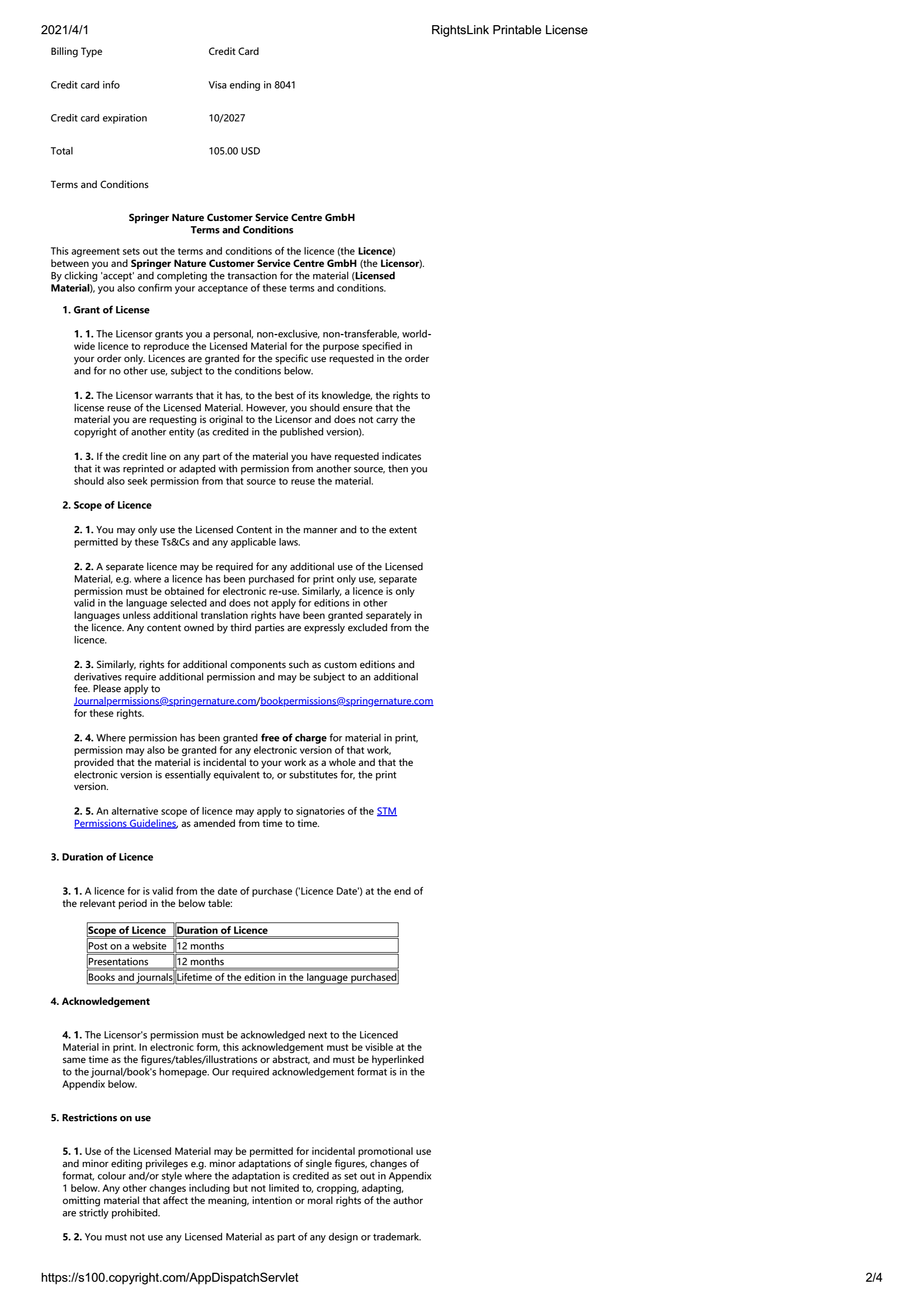


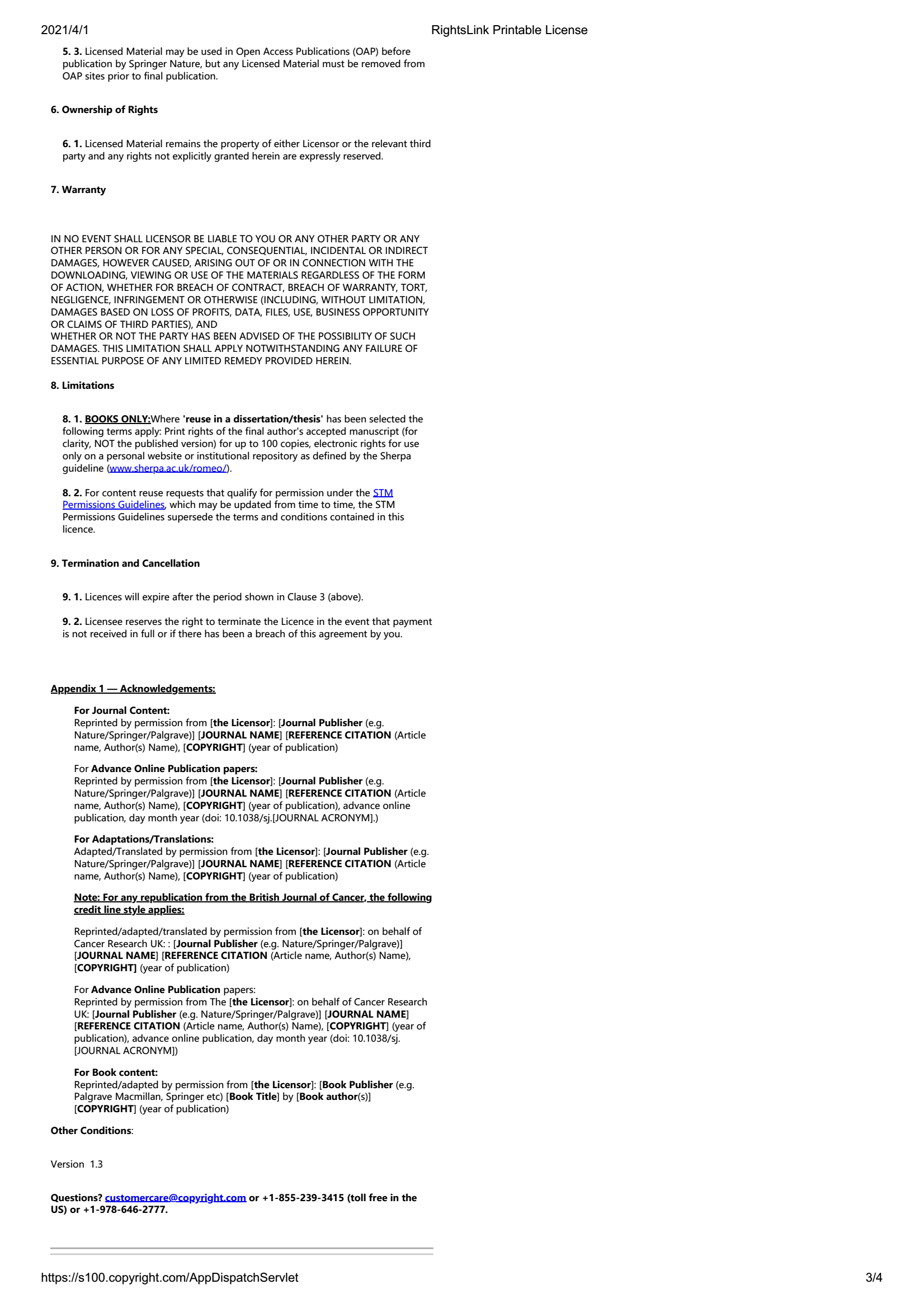


For Fig. 7i


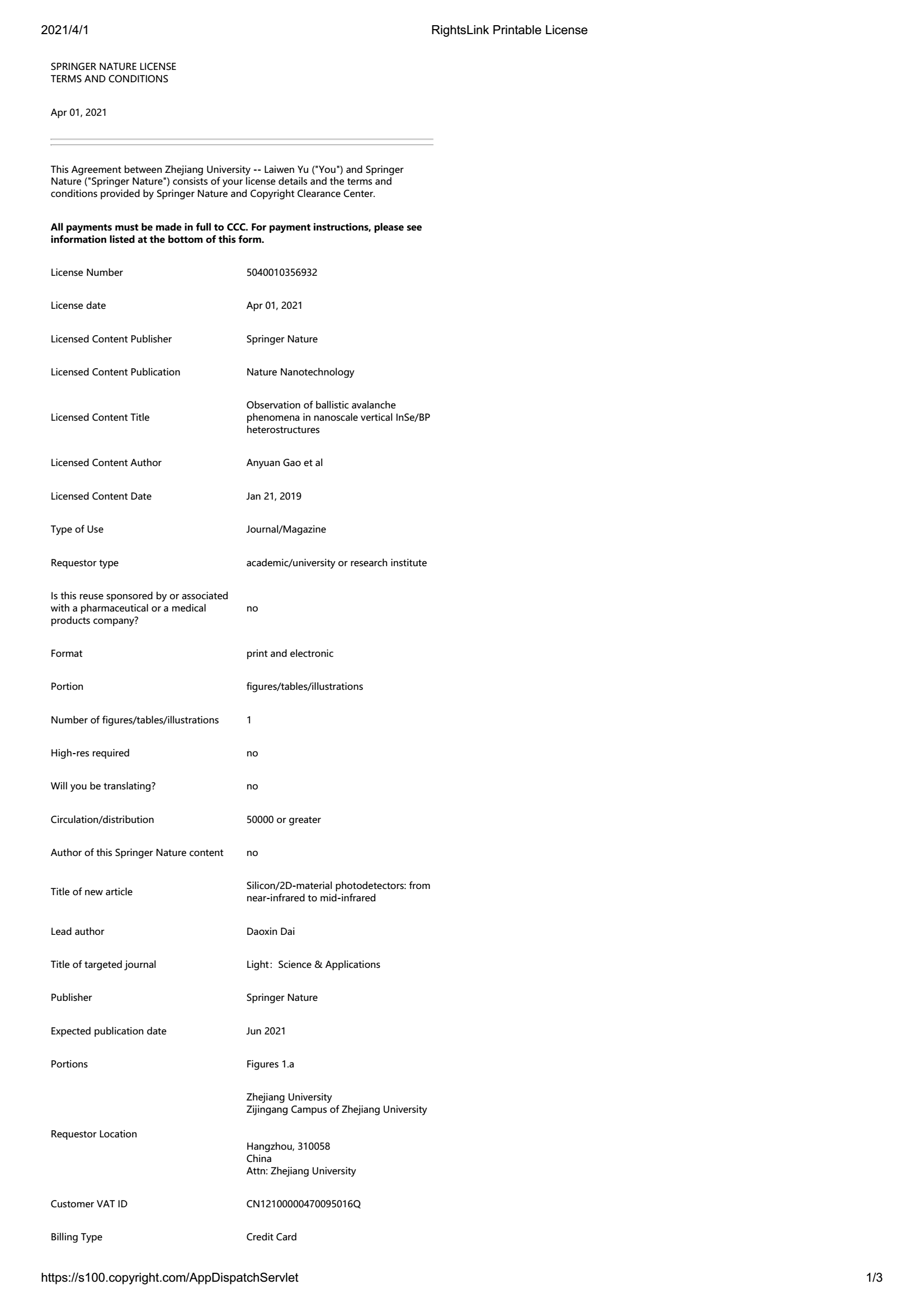

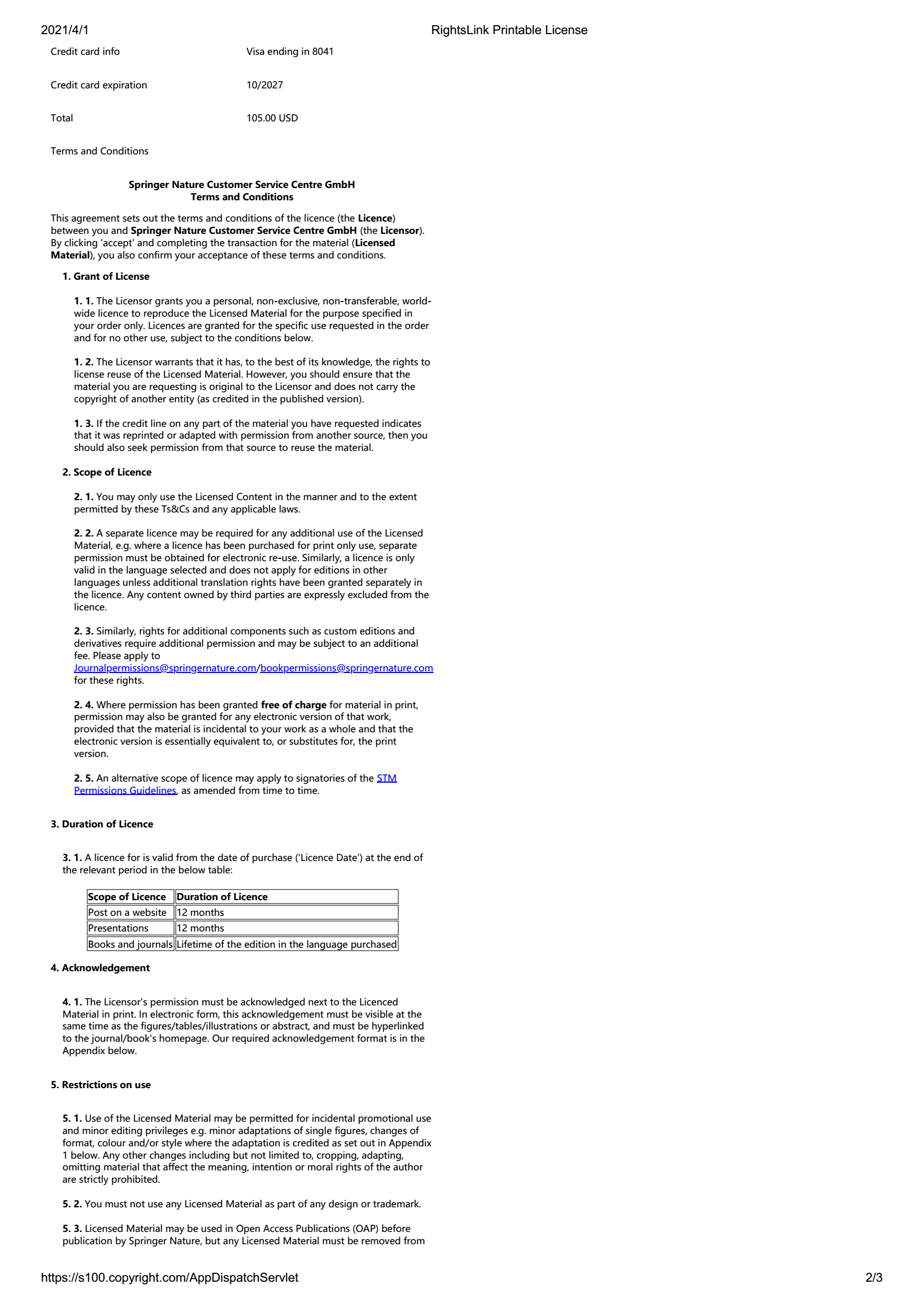


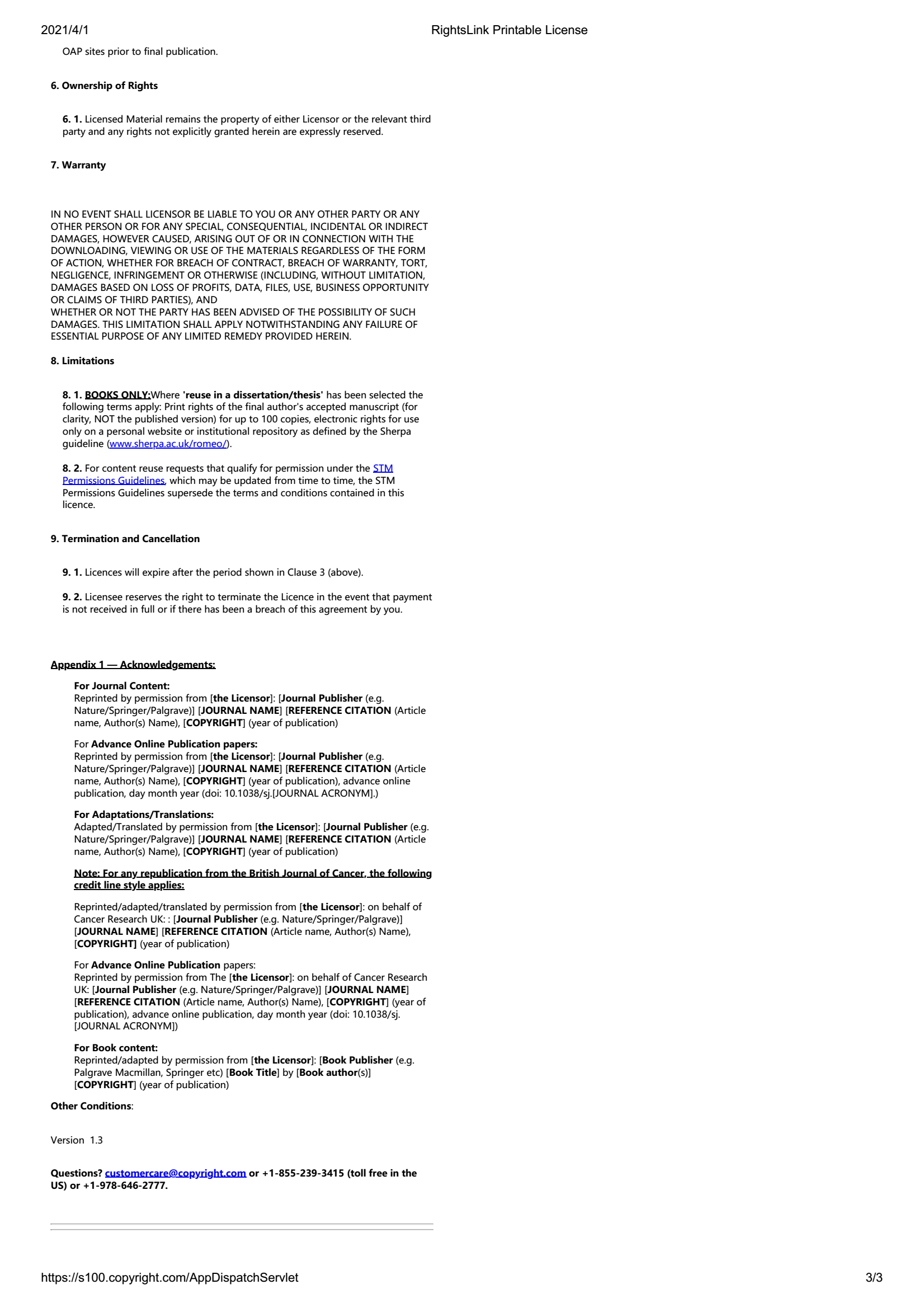


For Fig. 8a


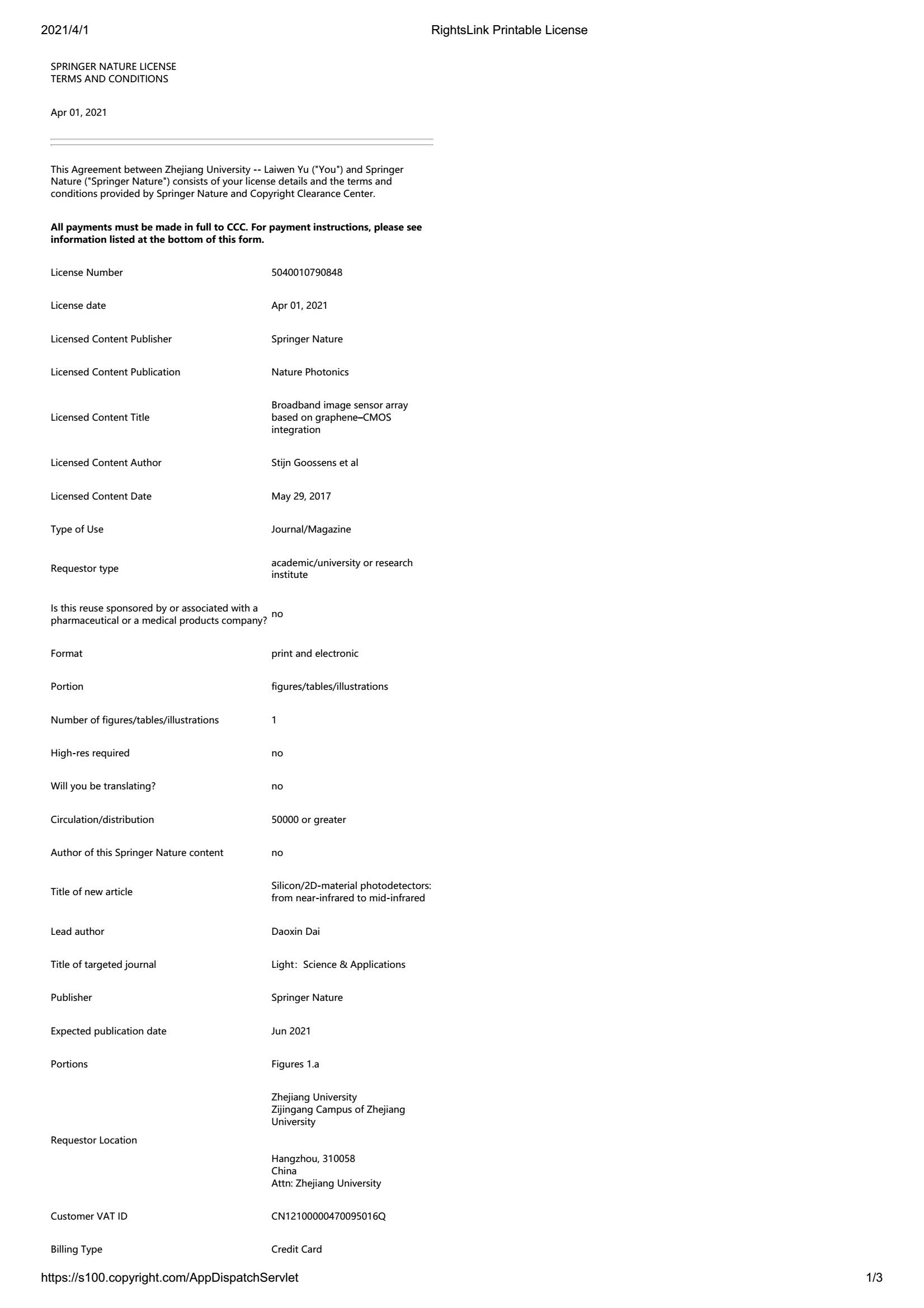

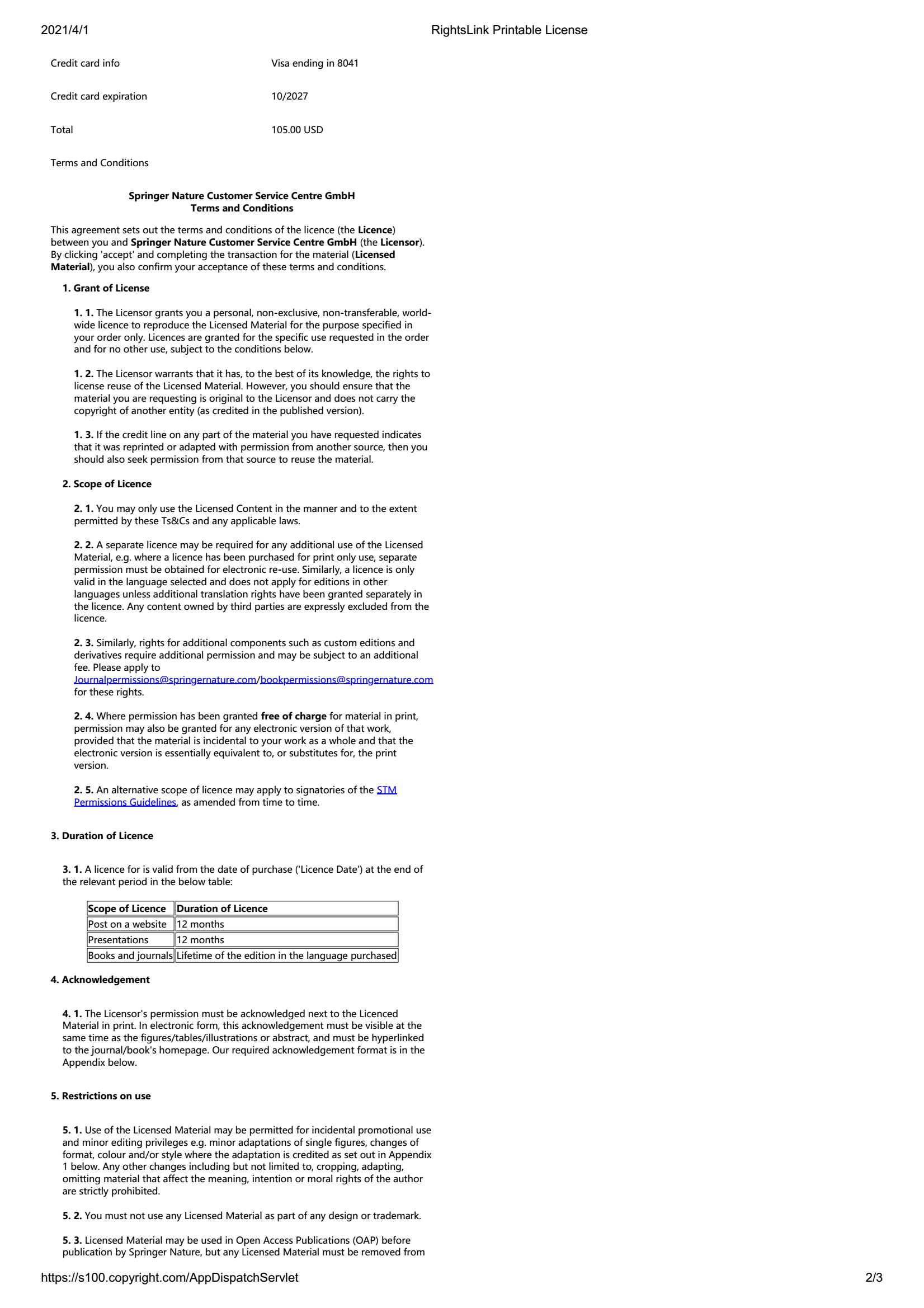


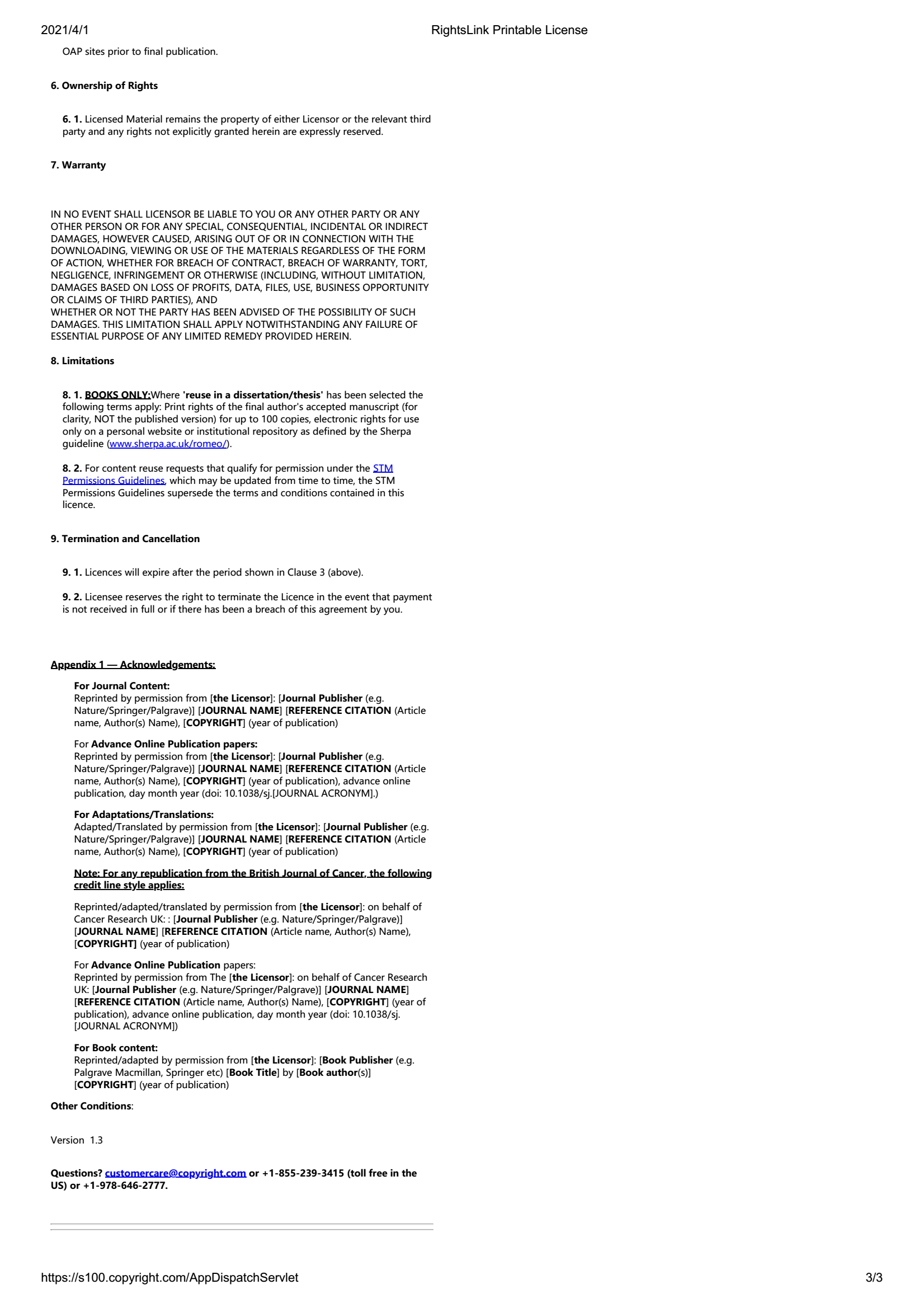


For Fig. 8b


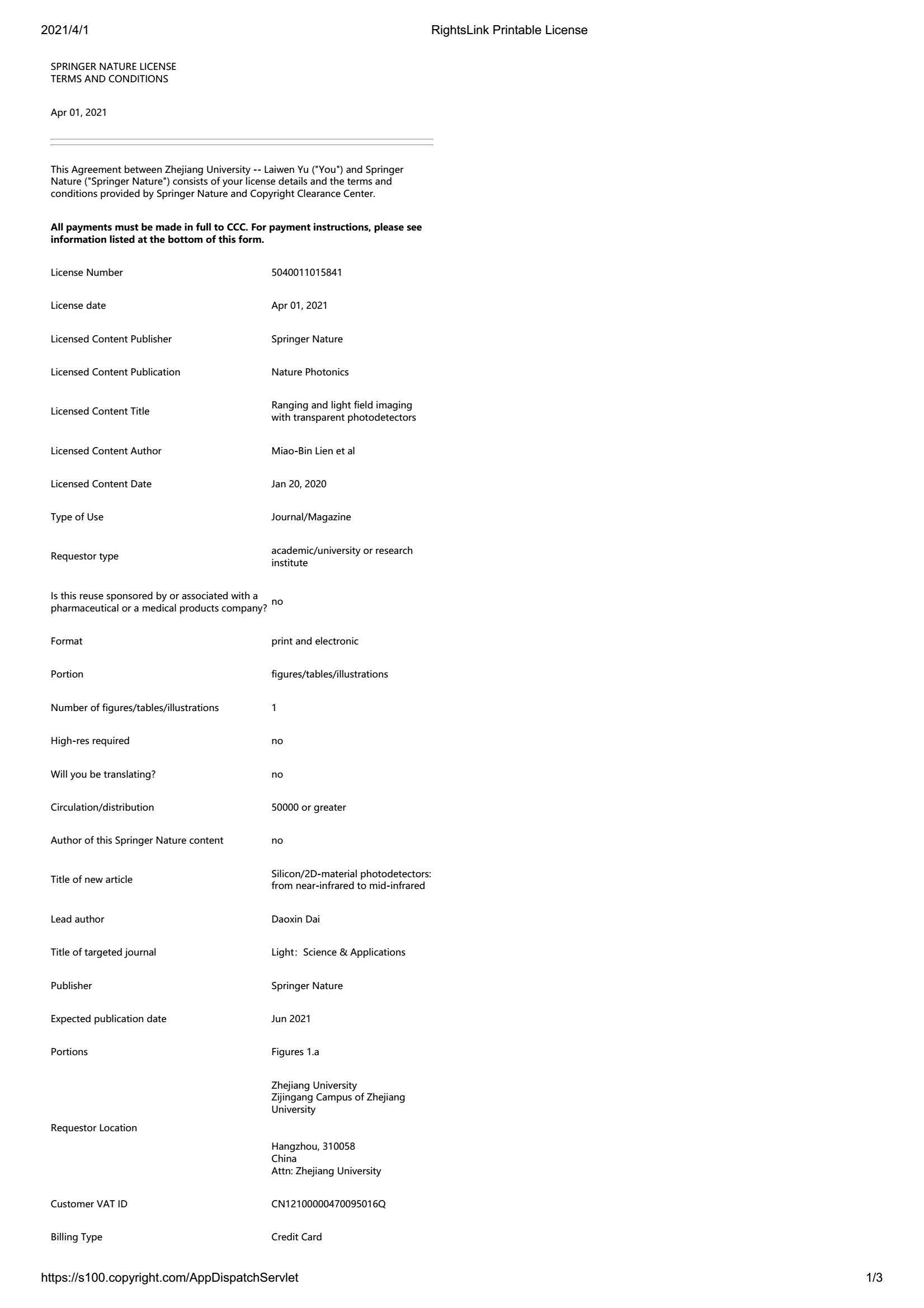

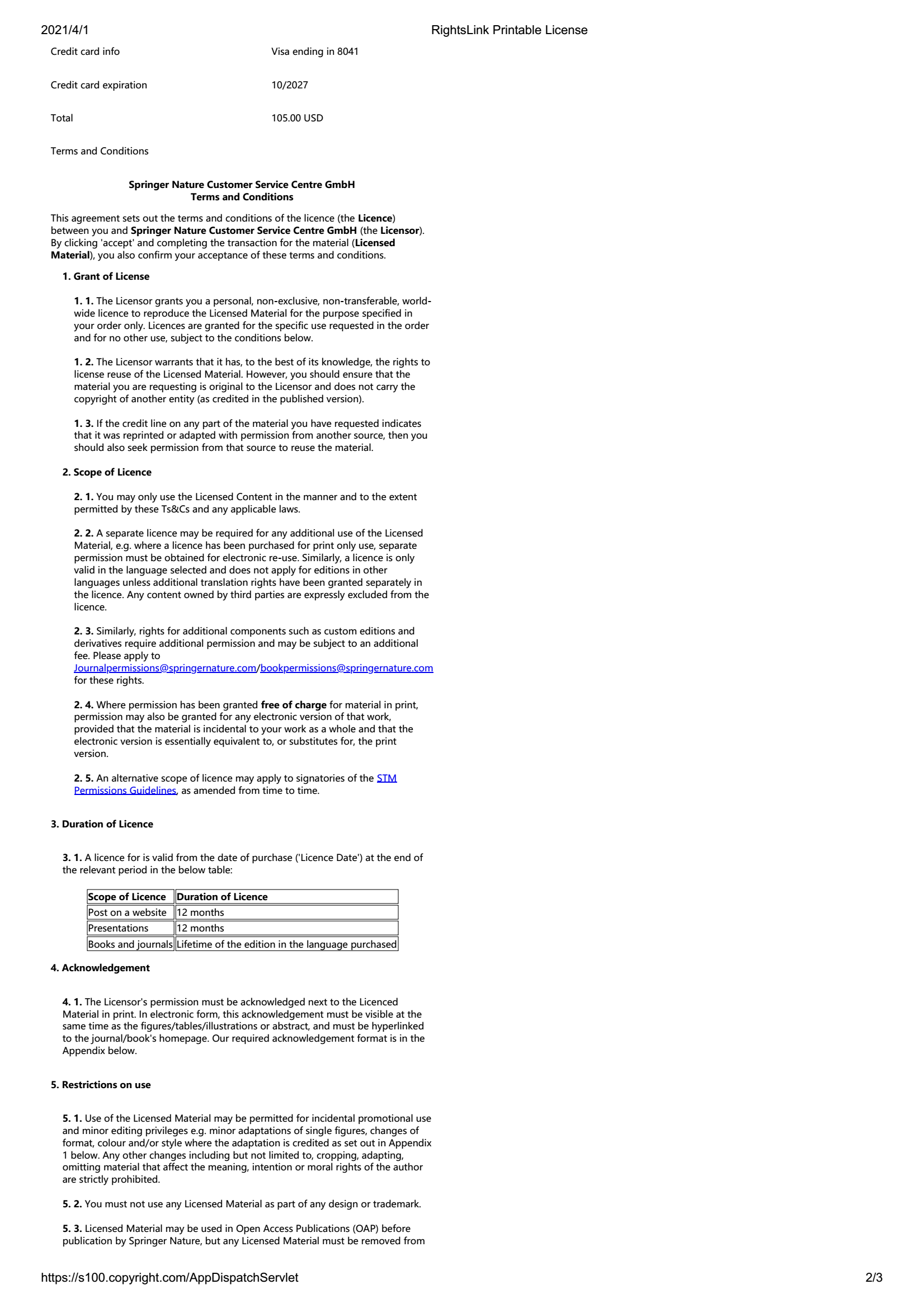


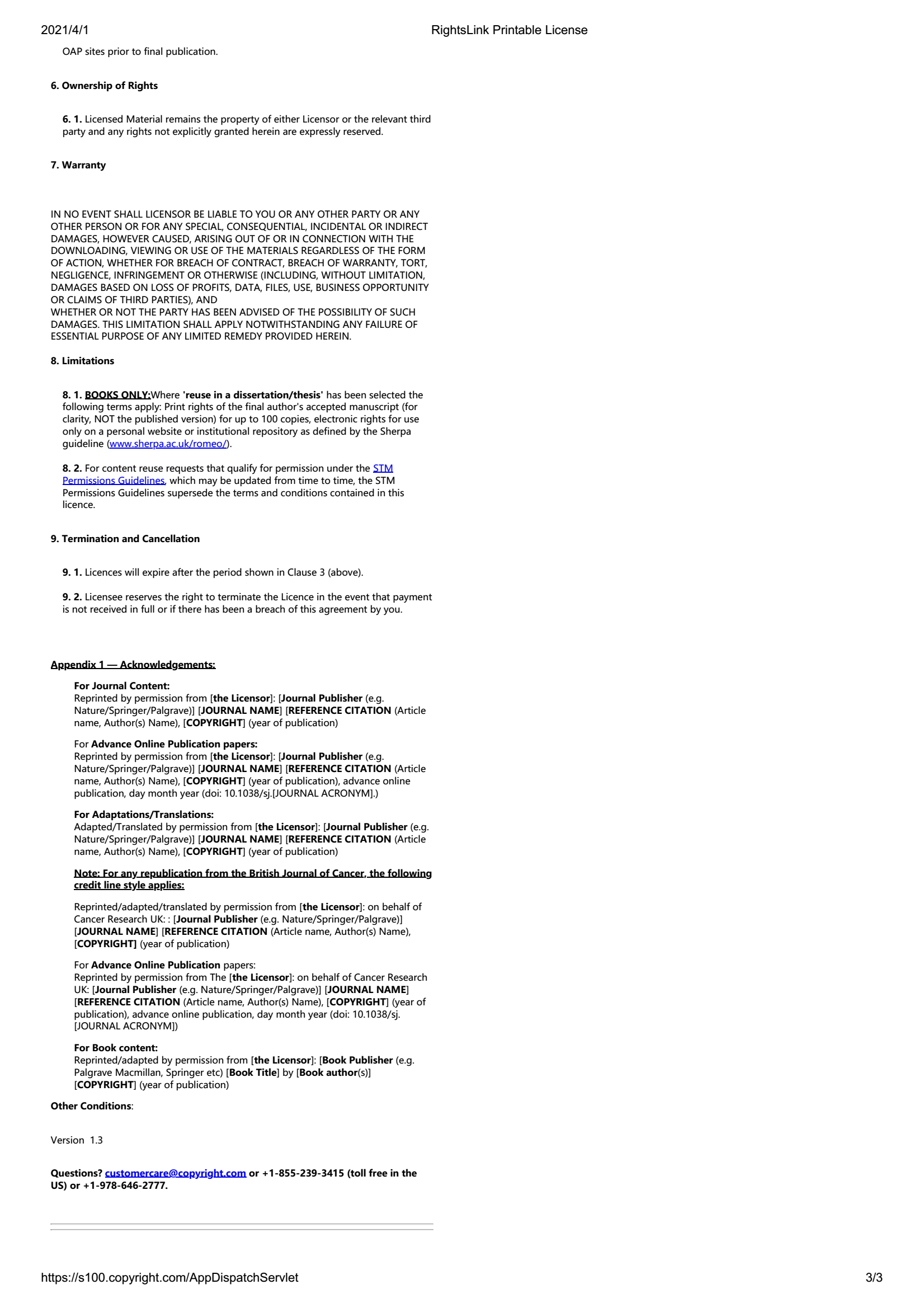


For Fig. 8c


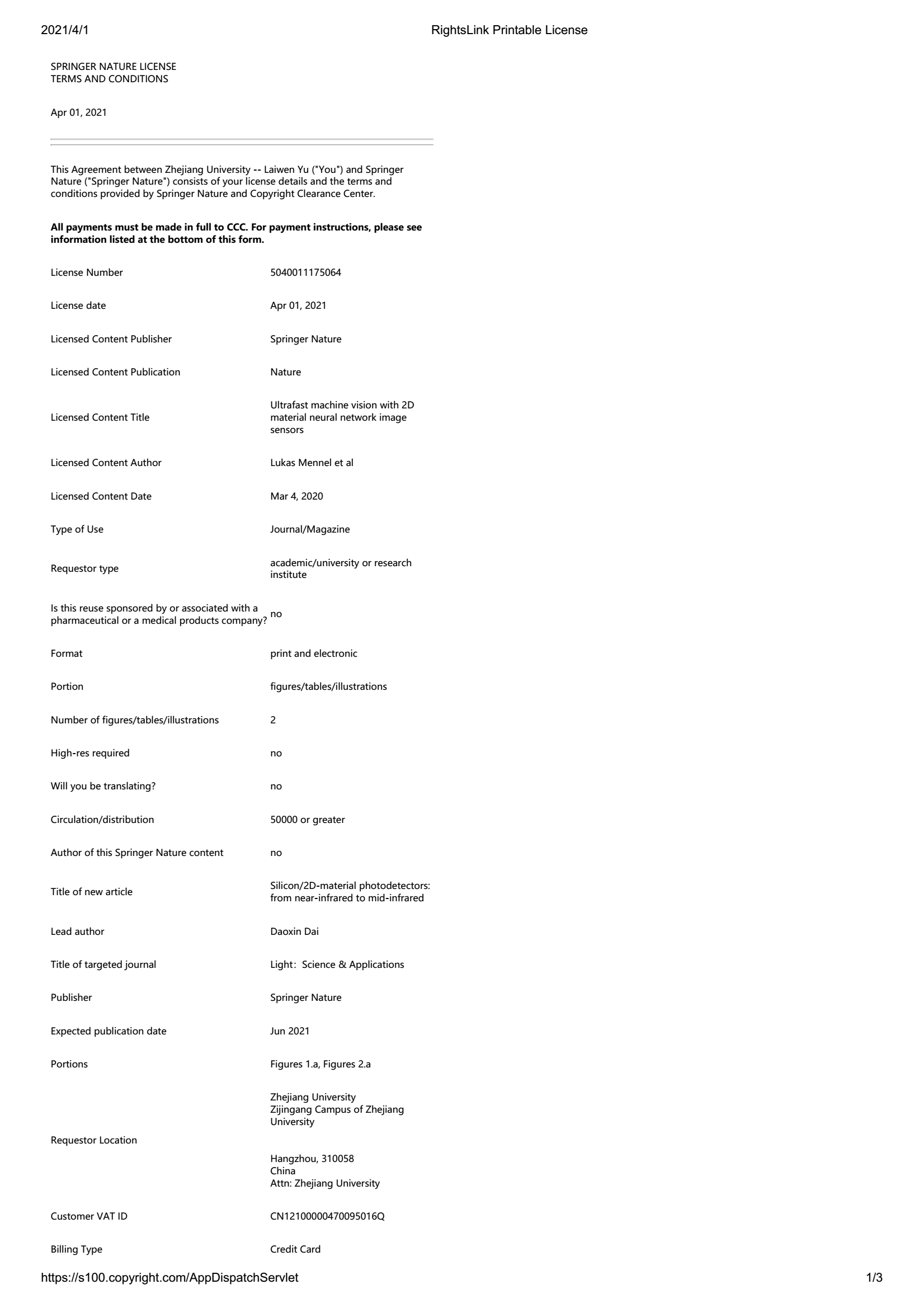

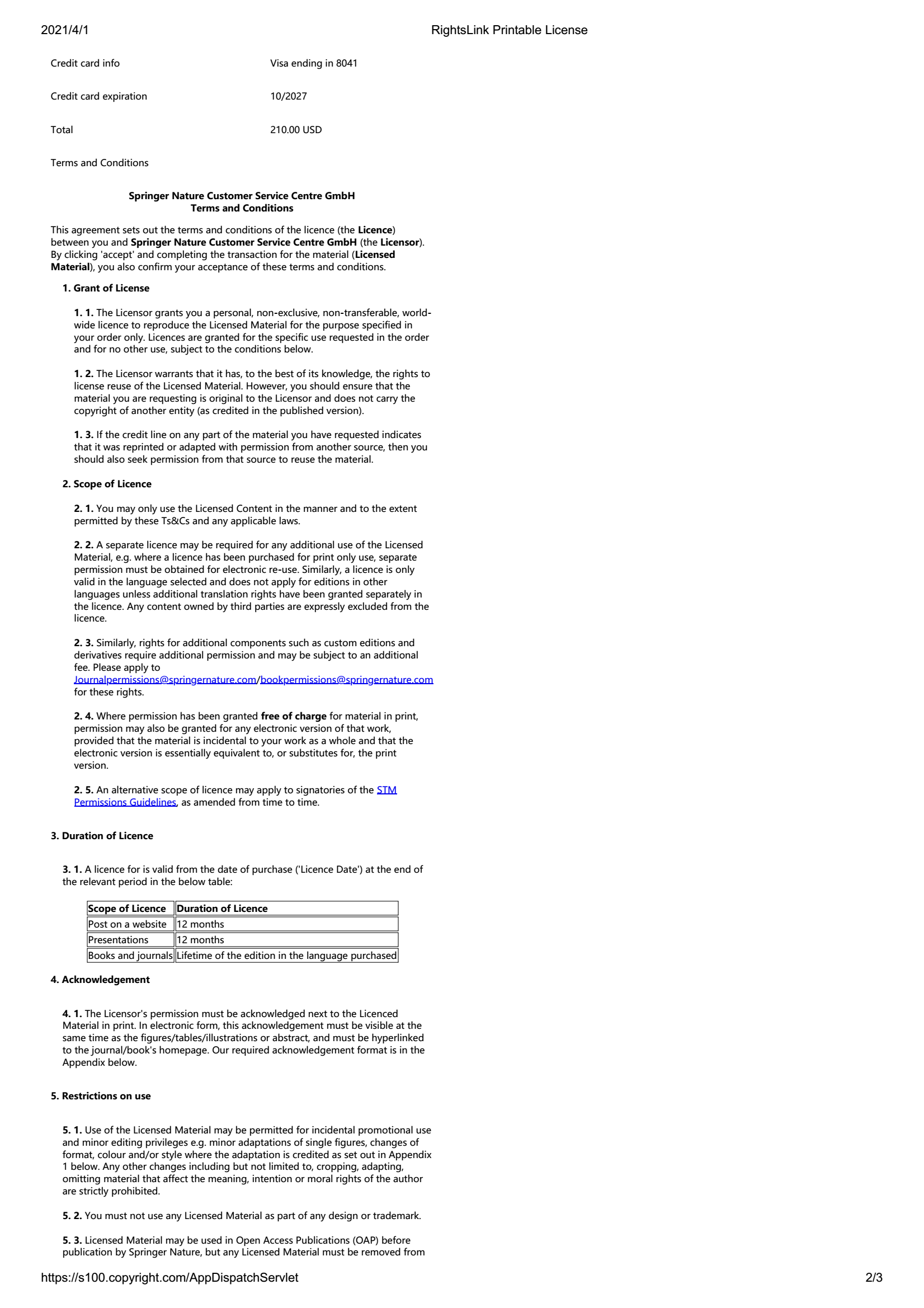


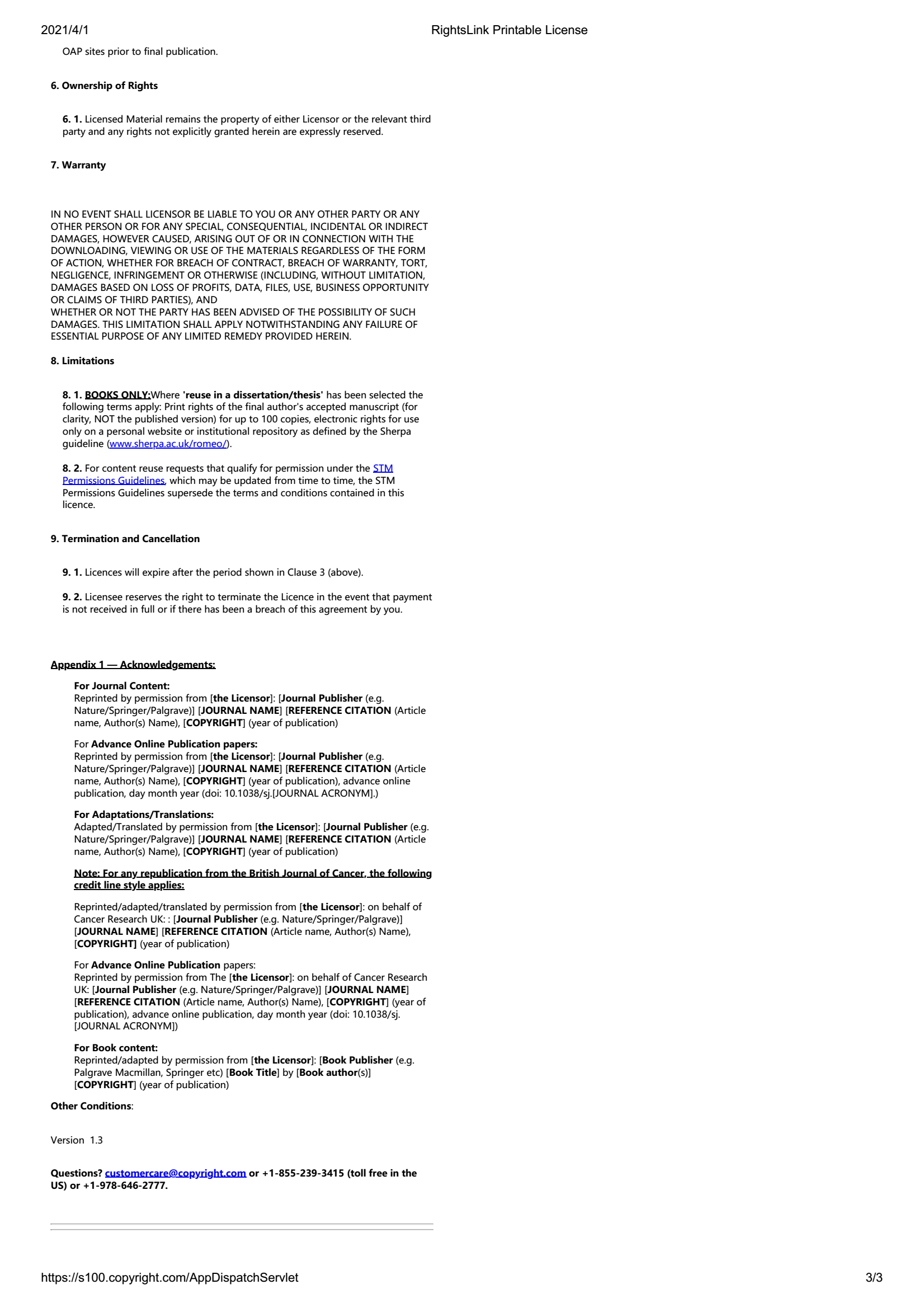


For Fig. 8d


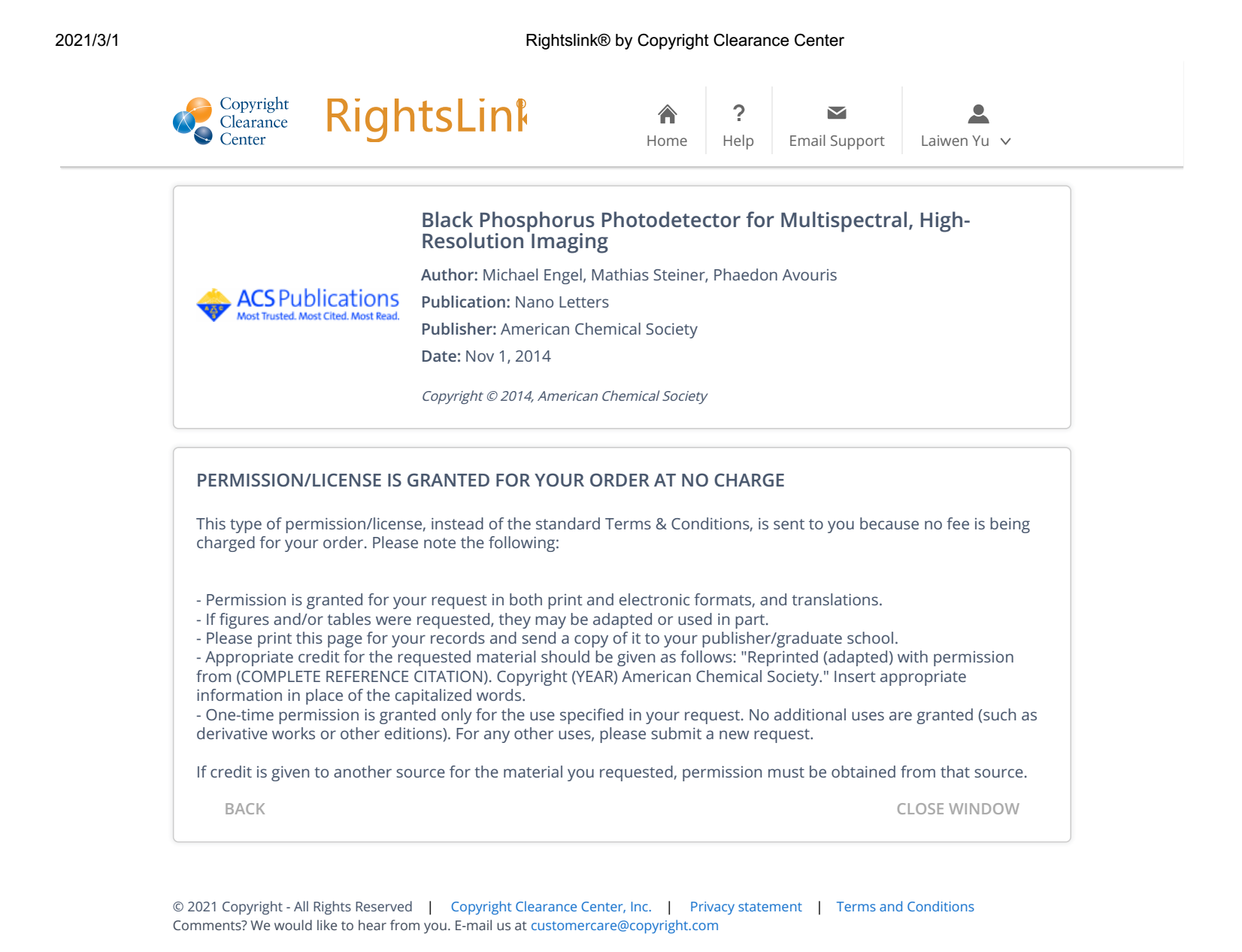

Supplement: Supplementary file 2 — Copyright permission files [file 41377_2021_551_MOESM2_ESM.docx]
